# Iodide/Nickel Co-Catalyzed Manganese-Mediated Denitrogenative Cross-Electrophile Coupling of Benzotriazinones with Alkyl Sulfonates

**DOI:** 10.3390/molecules30112397

**Published:** 2025-05-30

**Authors:** Yingying Hong, Xuanxuan Zhang, Gang Zou

**Affiliations:** School of Chemistry and Molecular Engineering, East China University of Science and Technology, 130 Meilong Rd, Shanghai 200237, China; y30220303@mail.ecust.edu.cn (Y.H.); y30220291@mail.ecust.edu.cn (X.Z.)

**Keywords:** cross-electrophile coupling, nickel catalysis, benzotriazinone, alkyl sulfonate

## Abstract

An efficient Ni-catalyzed, Mn-mediated denitrogenative cross-electrophile coupling of *N*-alkyl-1,2,3-benzotriazinones with alkyl tosylates and mesylates for access to *o*-alkyl secondary benzamides is reported. The method uses inexpensive non-anhydrous dimethyl acetamide (DMA) in combination with tetrabutyl ammonium iodide (TBAI) as a co-catalyst to convert sulfonates into iodides in situ. Scope and limitations of the protocol have been demonstrated by >30 examples with yields up to 91%, showing a large electronic effect from the *N*-substituent in benzotriazinones. An unexpected steric acceleration has been observed from the core of benzotriazinones, not only promising a highly efficient access to 2-alkyl-2,3-disubstituted benzamides but also shedding light on the rate-limiting steps in the catalytic cycle.

## 1. Introduction

Construction of a carboncarbon bond via transition-metal catalyzed cross-electrophile coupling (XEC) has become increasingly prevalent [1,2,3,4,5,6,7,8,9,10,11,12] since the breakthrough by Weix and Gong etc. just a decade ago [13,14,15,16]; although, the seminal work was reported by Durandetti et al. as early as 1996 [17,18]. Alkyl halides, in fact bromides, have proven to be the most useful alkyl source in XEC. Given that alcohol moieties are widely found in natural and synthetic chemicals, it is attractive to utilize alcohols or their non-halide derivatives. Although progress has recently been made in the direct use of alcohols as coupling partners in XEC by electrochemistry [19], co-catalysis [9,12,20,21,22,23,24], or in situ derivatization [9,12,25,26,27,28,29], the hydroxy group or its protic byproducts from in situ derivatization make alcohols incompatible to acidic proton sensitive processes. Alternatively, XEC using alcohol derivatives such as triflates [27,30], sulfonates [31,32,33], acetates [34,35], pivalates [36,37], and oxalates [38,39,40], etc., has been reported but is still relatively undeveloped, in particular, in coupling with counterparts derived from anilines [12,41]. We have recently reported Ni-catalyzed Mn-mediated denitrogenative XEC of benzotriazinones, an easily accessible anthranilic acid derivative [42], with alkyl halides to afford ortho-alkylated secondary benzamides both in air- and moisture-free solution [43] and under air-tolerant liquid-assisted grinding (LAG) conditions [44,45]. However, the air-tolerant LAG conditions were less effective for the corresponding alkyl tosylates, due to the protodenitrogenation of benzotriazinones as well as the slow radical formation vs. the fast hydrolysis of tosylates. Although preliminary experiments indicated that modest yields could possibly be obtained from solution reaction in DMA under rigorous air- and moisture-free conditions, unfortunately, the requirement of an expensive anhydrous strong polar solvent would hamper the potential applications of the XEC protocol using alkyl tosylates, the most economical and easy-to-handle alcohol derivative. It has been reported that the reactivities of benzotriazinones could be tuned by their *N*-substituents [42,46,47]. We envisioned that the protodenitrogenation of benzotriazinones could possibly be suppressed by tuning their *N*-substituents; meanwhile, the slow radical formation from alkyl tosylates could be circumvented by in situ conversion into the more reactive halides. Herein, we report an efficient Ni-catalyzed, Mn-mediated denitrogenative XEC of *N*-alkyl-1,2,3 benzotriazinones with alkyl tosylates and mesylates in inexpensive non-anhydrous DMA by using TBAI as co-catalyst.

## 2. Results and Discussion

**Scheme 1:**
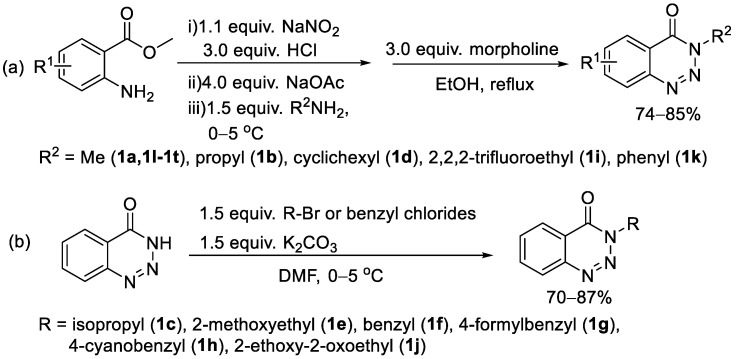
Synthesis of benzotriazinones via (**a**) diazotization/cyclization or (**b**) *N*-alkylation.

*N*-Substituted benzotriazinones were prepared by following the previously reported procedures [43,48] (see Supplementary Materials for details) (Scheme 1).

**Scheme 2:**
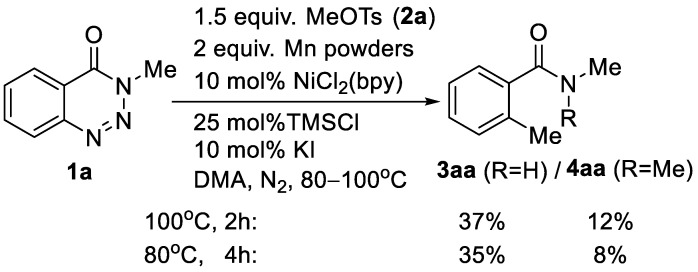
Preliminary results in denitrogenative XEC-based methylation of benzotriazinone **1a** with methyl tosylate **2a**.

Initially, a reaction of *N*-methyl benzotriazinone (**1a**) with methyl tosylate (**2a**) was selected as the model to develop an efficient denitrogenative XEC of benzotriazinones with alkyl sulfonates tolerant to non-anhydrous solvent based on the following considerations. (1) Benzotriazinones bearing an electron-neutral alkyl substituent at *N*(3) have shown a good balance between their stability and reactivity in XEC with alkyl/aryl halides [43,44,45,48]. (2) Selective and efficient installation of a methyl group at specific positions of an organic molecule is very important in medicinal chemistry, because of the so-called magic methyl effect [49,50,51]. (3) Methyl tosylate is more user-friendly than methyl halides (X = Cl, Br, I) under the most current conditions for XEC because of the toxicity and low boiling points of the latter compounds [52]. To minimize the deleterious effects of water residue, commercial non-anhydrous DMA was dried over a 4 Å molecular sieve (MS) overnight prior to use. Unfortunately, serious protodenitrogenation still took place in the MS-dried non-anhydrous DMA to give *N*-methylbenzamide in 26–29% yields using our previously developed 2,2′-bipyridine (bpy) supported nickel catalyst system [43], with 10 mol% NiCl_2_(bpy)/KI in the presence of 25 mol% trimethylsilyl chloride (TMSCl) as an activator for Mn powders at 80–100 °C (Scheme 2).

In addition, the ^1^H NMR spectrum showed that the XEC product **3aa** isolated by chromatography was still contaminated by the dimethylation side product, *N*, *N*, 2-trimethyl benzamide (**4aa**, 8–12%), which was confirmed by ^1^H NMR spectroscopy and GC-MS analyses of the crude products (See Supplementary Materials). The chromatographically inseparable side product **4aa** makes the reaction with MeOTs **2a** inconvenient as the model since the recrystallization after chromatography had to be used to obtain a spectroscopically pure product **3aa**. Fortunately, when ethyl tosylate **2b** was used in place of methyl **2a** under the otherwise identical conditions, the *N*-alkylation side-reaction was not observed. Therefore, ethyl tosylate (**2b**) was used in the following condition optimization.

A control experiment indicated both bromide and iodide salts could serve as the co-catalyst, although a significantly smaller promotion was observed from 10 mol% KBr than KI (Table 1, entries 1–4). We have reported that some quaternary ammonium halides could promote denitrogenative Sonogashira coupling of *N*-tosyl aryltriazenes [53], a sort of closely related noncyclic triazenes; therefore, we tested the effectiveness of quaternary ammonium co-catalyst, e.g., tetrabutyl ammonium bromide (TBAB) and iodide (TBAI). Although TBAB performed much less efficiently, to our delight, the yield of **3ab** increased to 86% with 10 mol% TBAI as the co-catalyst. The reaction could still proceed smoothly even with 3 mol% TBAI at a lower temperature, i.e., 60–80 °C (Table 1, entries 5–11). The loading of nickel catalyst NiCl_2_(bpy) could also be reduced to 5 mol% without decreasing **3ab** yield, although the reaction became slower to some extent (Table 1, entries 10 and 12). Direct combination of NiCl_2_ with bpy worked as effectively as NiCl_2_(bpy), making the catalyst system convenient to use. In fact, a combination of 5 mol% NiCl_2_(DME) with 5 mol% bpy gave the highest yield (89%) among the tested nickel precursors, e.g., NiCl_2_ (85%), Ni(OAc)_2_ (70%), and Ni(acac)_2_ (68%). 2,2-Bipyridine (bpy, 85% at 3 mol% catalyst loading) has proven to be the choice of supporting ligand for nickel catalyst since 1,10-phenanthroline (phen, 71%) and 4,4′-bis(tert-butyl)-2,2′-bipyridine (dtbbpy, 26%) performed less efficiently (Table 1, entries 19–21). The model reaction could also proceed in DMF, giving **3ab** in 74% yield while no reaction was observed in the other common solvents, e.g., DMSO, THF, DME, dioxane, and toluene, etc. The doses of TMSCl could be reduced to 5–10 mol%, while the reaction took place rather slowly in the absence of the activator (Table 1, entries 25–27). In sharp contrast, the use of 50 mol% TMSCl led to a lower yield (65%), possibly due to the formation of the less reactive ethyl chloride from tosylate **2b** via OTs/Cl exchange (vide infra). The yield of **3ab** could not be increased further with 2.0 equiv. tosylate **2b** while 1.0 equiv. led to a lower yield (72%) (Table 1, entries 28 and 29). Based on these experiments, the optimal conditions for the Ni-catalyzed, Mn-mediated denitrogenative XEC of benzotriazinones with alkyl tosylates were set as 1.5 equiv. tosylate, 2.0 equiv. Mn powders as the reductant, with 10 mol% TMSCl as activator, and 5 mol% NiCl_2_(DME)/bpy as the catalyst in the presence of 3 mol% TBAI co-catalyst at 60 °C under N_2_ in non-anhydrous DMA dried over 4 Å MS overnight prior to use.

Tolerance of the model reaction to H_2_O content in the DMA solvent was investigated under the optimal conditions (Table 1, entries 30–32). With direct use of commercial non-anhydrous DMA (up to 500 ppm H_2_O), the model reaction could still proceed smoothly to give the XEC product **3ab** in 86% yield. In fact, when the water content in the DMA was deliberately increased to 1000 ppm, the **3ab** yield just slightly decreased to 81% while no reaction could take place in DMA containing 5000 ppm water.

**Figure 1:**
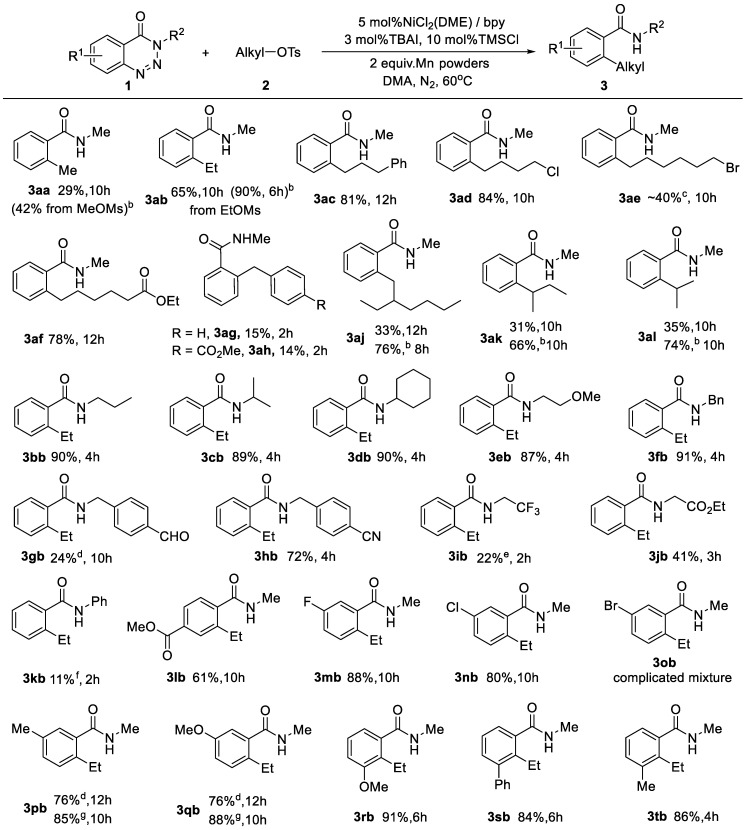
Scope and limitations. Reaction run at 1 mmol scale with respect to **1** with 1.5 equiv. **2** in 3 mL non-anhydrous DMA (dried over 4 Å MS). ^b^ At 80 °C and a small amount (~1.6%) of isomer in GC-MS (see Supplementary Materials). ^c^ Contaminated by halo-exchange. ^d^ Incomplete conversion of benzotriazinones. ^e^ Proto-denitrogenation product *N*-2,2,2-trifluoroethyl benzamide isolated in 42% yield. ^f^ *N*-phenyl benzamide isolated in 45% yield. ^g^ 5 mol% NiCl_2_(DME)/10 mol% bpy used.

Scope and limitations of the iodide/nickel co-catalyzed, Mn-mediated denitrogenative XEC of benzotriazinones with alkyl sulfonates were explored briefly (Figure 1). Besides the undesired *N*-methylation, methyl tosylate **2a** appeared to be less effective than its homologues. In fact, spectroscopically pure (NMR) **3aa**, as determined by ^1^H NMR spectroscopy, was obtained in only 29% yield by recrystallization in CH_2_Cl_2_/petroleum ether after column chromatography to remove the *N*-methylation side-product *N*, *N*, 2-trimethylbenzamide (**4aa**). Mesylates MeOMs (**2a’**) and EtOMs (**2b’**), albeit less reactive than tosylates, could still react effectively at a slightly higher temperature (80 °C), giving the methylation (**3aa**) and ethylation (**3ab**) products in 42% and 90% yields, respectively. The better result from the reaction of MeOMs (**2a’**) than MeOTs (**2a**) could possibly be attributed to its relatively lower reactivity in the undesired *N*-methylation. In fact, formation of **4aa** decreased to <3% (^1^H NMR) in the reaction with MeOMs from about 10% with MeOTs.

A high chemoselectivity of Csp^3^-OTs vs. Csp^3^-Cl could be achieved since the reaction of 3-chloropropyl tosylate **3d** with **1a** gave **3ad** in 84% yield while the Csp^3^-Br (**2e**) competed strongly. In addition, ^1^H NMR and GC-MS (see Supplementary Materials) showed Cl/Br/I scramble in the oily XEC product (**3ae**) from the reaction of **2e** due to halo-exchange. The denitrogenative XEC-based benzylation of **1a** gave **3ag** and **3ah** in very low yields (14–15%), although benzyl tosylates disappeared completely in about 2 h due to their serious dimerization. Allyl tosylate (**2i**) performed poorly under the optimal conditions since only trace XEC product could be detected in GC-MS analysis (see Supplementary Materials). Steric hindrance from alkyl tosylates could be overcome by slightly increasing the reaction temperature to 80 °C. For example, 2-ethylhexyl tosylate **2j** reacted to afford **3aj** in 33% (12 h) and 76% (8 h) yields at 60 °C and 80 °C, respectively. Similarly, secondary alkyl tosylates, e.g., isopropyl (**2k**) and sec-butyl (**2l**), also required 80 °C to react effectively.

Electronic and steric influences from the benzotriazinone counterpart were then investigated. The steric effects from the *N*-alkyl substituents appeared to be negligible since *N*-propyl (**1b**, 90%), *N*-isopropyl (**1c**, 89%), *N*-cyclichexyl (**1d**, 90%), *N*-2-methoxyethyl (**1e**, 87%), and *N*-benzyl (**1f**, 91%) benzotriazinones reacted as smoothly as the *N*-methyl one (**1a**). However, an electron-deficient alkyl group at *N*(3) in benzotriazinones, e.g., 2,2,2-trifluoroethyl (**1i**) and 2-ethoxy-2-oxoethyl (**1j**), led to serious proto-denitrogenation, thus resulting in low yields for the desired XEC product **3ib** (22%) and **3jb** (41%). In fact, *N*-(4-cyanobenzyl) benzotriazinone (**1h**, 72%, 4 h) gave a relatively lower yield than the corresponding benzyl one (**1f**, 91%, 4 h). The reaction of *N*-(4-formylbenzyl)benzotriazinone (**1g**, 24%, 10h) became sluggish, implying incompatibility of the CHO group with the catalyst system beside the electronic effects. Proto-denitrogenation became dominate for *N*-phenyl benzotriazinone **1k** most likely because, similar to an electron-deficient alkyl group at the *N*(3) atom, the *N*/aryl conjugation could also decrease the stability of the key azanickelacycle intermediates in the catalytic cycle (vide infra). A mild electron-withdrawing group, e.g., 7-CO_2_Me (**1l**, 61%, 10 h), 6-F (**1m**, 88%, 10 h), and 6-Cl (**1n**, 80%, 10 h), or electron-donating one, e.g., 6-Me (**1p**, 76%, 12 h) and 6-OMe (**1q**, 76%, 12 h), on the core of benzotriazinones could be tolerated to a large extent. The C_Ar_-Br bond competed seriously with the C_Ar_-*N* in the triazene moiety, giving a rather complicated mixture. Surprisingly, a steric acceleration was observed from benzotriazinones in the reaction with **2b**. For example, *o*-substituted **1r** (8-OMe) and **1t** (8-Me) reacted faster and afforded higher yields for **3rb** (91%, 6 h) and **3tb** (86%, 4 h) than their *para*-isomers **1p** (6-Me, 76%, 12 h) and **1q** (6-OMe, 76%, 12 h). Even 8-phenyl benzotriazinone (**1s**) could react smoothly to give the sterically demanding product **3sa** in 84% yield (6 h). To the best of our knowledge, this steric acceleration has not been previously reported in transition-metal catalyzed transformations of benzotriazinones and would promise highly efficient access to 2-alkyl-2,3-disubstited benzamides.

**Scheme 3:**
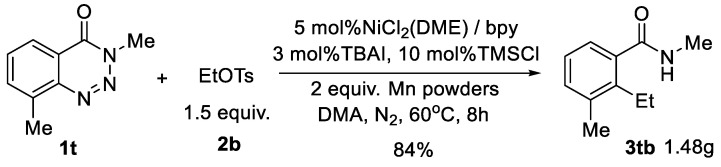
Gram-scale synthesis of 2-ethyl-*N*,3-dimethylbenzamide (**3tb**).

The practical utility of iodide/nickel co-catalyzed Mn-mediated denitrogenative XEC was demonstrated by the gram-scale synthesis of 2-ethyl-*N*,3-dimethylbenzamide (**3tb**) under the optimal conditions (Scheme 3). The reaction of 3,8-dimethylbenzotriazinone (**1t**) with ethyl tosylate **2b** (1.5 equiv.) at 10 mmol scale proceeded smoothly to afford **3tb** in 84% yield in 8 h.

**Scheme 4:**
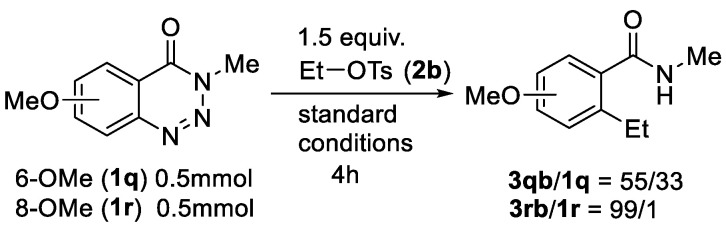
Competing reaction of the sterically different benzotriazinone isomers.

To unmistakably confirm the unique steric acceleration from benzotriazinones, a competing experiment using a 1:1 mixture of 6-OMe (**1q**) and 8-OMe (**1r**) isomers was carried out (Scheme 4). GC-MS analysis (see Supplementary Materials) of the reaction mixture at 4 h showed that almost all of the more steric isomer **1r** was converted into XEC product **3rb** (**1r**/**3rb** < 1/99) without detectable proto-denitrogenation while about one-third (33% GC area) of the less steric one **1q** remained in the solution, producing **3qb** in only 55% along with about 12% 3-methoxy-*N*-methylbenzamide from proto-denitrogenation. These results have not only confirmed the steric acceleration effect from the core of the benzotriazinone counterpart but also provide a clue to the rate-limiting steps in catalytic cycle (vide infra).

**Scheme 5:**
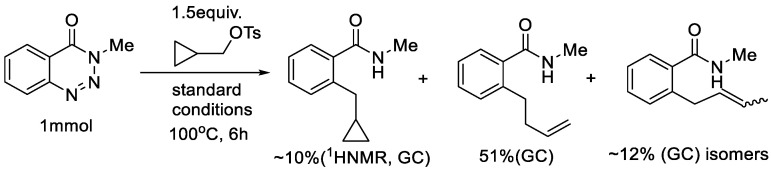
Product distribution in radical clock experiment.

A radical clock experiment using cyclopropylmethyl tosylate gave the XEC products as a mixture of 1-(but-3-enyl)-*N*-methylbenzamide (51%) and its internal isomers (in total 12%, GC) as major products along with ~10% (^1^H NMR and GC) normal coupling one 1-(cyclopropylmethyl)-*N*-methylbenzamide (see Supplementary Materials), consistent with a caged radical process although a combination of a free radical process with a minor non-radical one could not be excluded (Scheme 5).

**Scheme 6:**
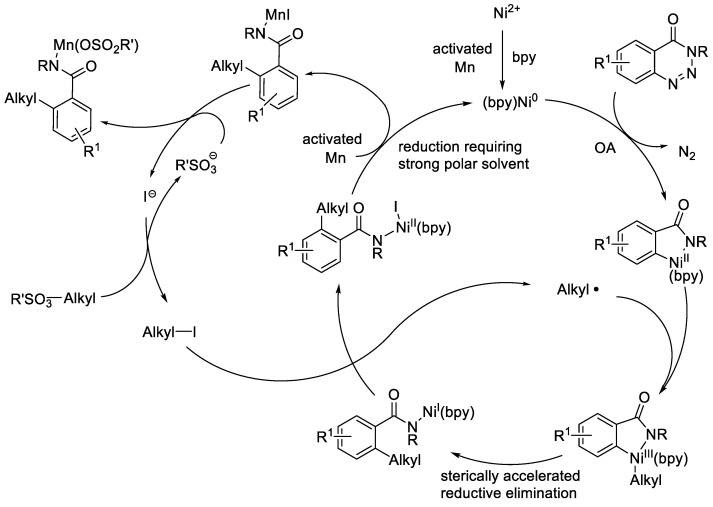
A plausible catalytic cycle for iodide/nickel co-catalyzed, manganese-mediated denitrogenative XEC of benzotriazinones with alkyl sulfonates.

Based on the experimental results and the literature on Ni-catalyzed cross-coupling [6,12,14,43,54,55], a plausible mechanism is proposed for the iodide/nickel co-catalyzed, manganese-assisted denitrogenative cross-coupling of benzotriazinones with alkyl sulfonates (Scheme 6). Given the low catalytic efficiency in the absence of iodide co-catalyst, it is reasonable to propose that alkyl sulfonates should be converted into iodides at first and then follow the Ni(0-II-III-I-II) catalytic cycle for Ni-catalyzed denitrogenative XEC of benzotriazinones with alkyl halides. Denitrogenative oxidative addition of Ni(0) to C_Ar_-*N* in benzotriazinones initiates the nickel catalysis to produce Ni(II) azanickelacycle; a subsequent single electron oxidation by alkyl radical generated from in situ-formed alkyl iodides leads to alkyl Ni(III) azanickelacycle. An aryl group or electron-deficient alkyl substituent at the *N*(3) atom of benzotriazinones decreases the stability of the subsequently formed Ni(II/III) azanickelacycle intermediates in the catalytic cycle, increasing the proto-denitrogenation by water residue in the system. Reductive elimination from the alkyl Ni(III) azanickelacycle could be sterically accelerated by 8-substitent in benzotriazinones because of the crowded environment around the Ni center [56,57,58], which should also improve the compatibility of the corresponding Ni(II/III) azanickelacycles to the protic source since the proto-denitrogenation was only observed from 6-OMe (**1q**) isomer in the competing reaction (vide supra). The observed steric acceleration from the 8-substituent at the core of benzotriazinones implies that there are sterically switchable rate-determining steps in the catalytic cycle.

## 3. Materials and Methods

### 3.1. General Information

All reactions were carried out under an N_2_ atmosphere unless otherwise stated. Chemicals obtained from commercial sources were used as received. The reaction progress was monitored by TLC. Column chromatography was performed on 300–400 mesh silica gel. ^1^H and ^13^C NMR spectra were recorded in CDCl_3_ at ambient temperature on a Bruker Avance Ⅲ (400 MHz) instrument (Bruker BioSpin GmbH, Rheinstetten, Germany). Coupling constants J are reported in Hz. Proton coupling patterns were described as singlet (s), doublet (d), triplet (t), quartet (q), and multiple (m). All new compounds were further characterized by high-resolution mass spectra (HRMS) using an Agilent mass spectrometer (HR-TOF-ESI). Melting points were determined on a digital melting point apparatus, and temperatures were uncorrected. All the known products were characterized by comparing their NMR with those reported in the literature, and the new compounds were further characterized by HRMS.

### 3.2. General Procedure for the Iodide/Nickel Co-Catalyzed Manganese-Mediated Denitrogenative Cross-Electrophile Coupling of Benzotriazinones with Alkyl Sulfonates

To a 25 mL dry flask, benzotriazinone (1.0 mmol, 1.0 equiv.), alkyl sulfonate (1.5 mmol, 1.5 equiv.), NiCl_2_(DME) (0.011 g, 0.05 mmol, 5 mol%), bpy (0.008 g, 0.05 mmol, 5 mol%), TBAI (0.011 g, 0.03 mmol, 3 mol%), and Mn powder (0.110 g, 2.0 mmol, 2.0 equiv.) were added. After replacement of the air in the flask with N_2_ using a standard Schlenk line, TMSCl (13 μL, 0.1 mmol, 10 mol%) and non-anhydrous DMA (dried over 4 Å MS, 3 mL) were added by syringe. The mixture was stirred at room temperature for 20 min and then heated at 60 °C (oil bath). The reaction progress was monitored by TLC until the consumption of **1** or for 12 h. Then, the reaction was quenched with 10% HCl (2 mL) and diluted and extracted with ethyl acetate (3 × 10 mL). The combined organic phase was dried over Na_2_SO_4_, filtered, and evaporated by rotavapor to give the crude product, which was purified by column chromatography on silica gel with petroleum ether/ethyl acetate eluent to afford product **3**.

*N,2-Dimethylbenzamide* (**3aa**) [59] White solid (43.5 mg, 29%; 62.7 mg, 42% from MeOMs at 80 °C); m.p. = 75–77 °C. ^1^H NMR (400 MHz, CDCl_3_) δ(ppm): 7.31–7.26 (m, 2H), 7.20–7.14 (m, 2H), 5.99 (s, 1H), 2.94 (d, *J* = 4.8 Hz, 3H), 2.41 (s, 3H). ^13^C NMR (100 MHz, CDCl_3_) δ(ppm): 170.9, 136.5, 135.9, 130.9, 129.7, 126.7, 125.6, 26.5, 19.7.

*2-Ethyl-N-methylbenzamide* (**3ab**) [60] White solid (144.2 mg, 88%; 105.9 mg, 65% from EtOMs at 60 °C; 146.3 mg, 90% from EtOMs at 80 °C); m.p. = 92–93 °C. ^1^H NMR (400 MHz, CDCl_3_) δ(ppm): 7.34–7.23 (m, 3H), 7.18–7.15 (t, *J* = 7.2 Hz, 1H), 5.96 (s, 1H), 2.94 (d, *J* = 4.8 Hz, 3H), 2.77 (q, *J* = 7.6 Hz, 2H), 1.21 (t, *J* = 7.6 Hz, 3H). ^13^C NMR (100 MHz, CDCl_3_) δ(ppm): 171.1, 142.1, 136.3, 129.7, 129.2, 126.7, 125.6, 26.4, 26.3, 15.7.

*N-Methyl-2-(3-phenylpropyl)benzamide* (**3ac**) [43] White solid (204.6 mg, 81%); m.p. = 80–82 °C. ^1^H NMR (400 MHz, CDCl_3_) δ(ppm): 7.32–7.24 (m, 4H), 7.21–7.14 (m, 5H), 5.80 (s, 1H), 2.89 (d, J = 5.2 Hz, 3H), 2.79 (t, J = 8.0 Hz, 2H), 2.65 (t, J = 7.6 Hz, 2H), 1.97–1.89 (m, 2H). ^13^C NMR (100 MHz, CDCl_3_) δ(ppm): 170.9, 142.2, 140.3, 136.5, 130.0, 129.7, 128.4, 128.3, 126.9, 125.8, 125.7, 35.7, 33.0, 32.8, 26.5.

*2-(4-Chlorobutyl)-N-methylbenzamide* (**3ad**) White solid (190.7 mg, 84%); m.p. = 36–38 °C. ^1^H NMR (400 MHz, CDCl_3_) δ(ppm): 7.34–7.27 (m, 2H), 7.24–7.16 (m, 2H), 5.94 (s, 1H), 3.53 (t, *J* = 6.0 Hz, 2H), 2.95 (d, *J* = 5.2 Hz, 3H), 2.78 (t, *J* = 7.6 Hz, 2H), 1.82–1.71 (m, 4H). ^13^C NMR (100 MHz, CDCl_3_) δ(ppm): 170.9, 140.2, 136.4, 130.0, 129.9, 126.8, 126.0, 44.9, 32.3, 32.3, 28.7, 26.6. HRMS (ESI) *m*/*z*: [M + Na]^+^ calcd for C_12_H_16_ClNONa 248.0818; found 248.0820.

*2-(6-Bromohexyl)-N-methylbenzamide* (**3ae**) Yellow oil (118.0 mg (**3ae** with inseparable halo-exchange products) ~40%). ^1^H NMR (400 MHz, CDCl_3_) δ(ppm): 7.34–7.29 (m, 2H), 7.23–7.16 (m, 2H), 5.82 (s, 1H), 3.39 (t, *J* = 6.8 Hz, 2H), 2.98 (d, *J* = 5.2 Hz, 2H), 2.76 (t, *J* = 8.0 Hz, 2H), 1.88–1.81 (m, 2H), 1.66–1.58 (m, 2H), 1.49–1.34 (m, 4H).

*Ethyl 6-(2-(methylcarbamoyl)phenyl)hexanoate* (**3af**) Yellow oil (216.0 mg, 78%). ^1^H NMR (400 MHz, CDCl_3_) δ(ppm): 7.33–7.28 (m, 2H), 7.22–7.15 (m, 2H), 5.95 (s, 1H), 4.09 (q, *J* = 7.2 Hz, 2H), 2.96 (d, *J* = 4.8 Hz, 3H), 2.76 (t, *J* = 7.6 Hz, 2H), 2.27 (t, *J* = 7.6 Hz, 2H), 1.67–1.58 (m, 4H), 1.39–1.32 (m, 2H), 1.23 (t, *J* = 7.2 Hz, 3H). ^13^C NMR (100 MHz, CDCl_3_) δ(ppm): 173.9, 171.0, 140.5, 136.4, 129.9, 129.6, 126.8, 125.7, 60.2, 34.2, 32.9, 31.0, 28.8, 26.5, 24.7, 14.2. HRMS (ESI) *m*/*z*: [M + Na]^+^ calcd for C_16_H_23_NO_3_Na 300.1576; found 300.1577.

*2-Benzyl-N-methylbenzamide* (**3ag**) [45] White solid (34.3 mg, 15%); m.p. = 108–110 °C. ^1^H NMR (400 MHz, CDCl_3_) δ(ppm): 7.33–7.30 (m, 2H), 7.26–7.20 (m, 4H), 7.18–7.14 (m, 3H), 5.69 (s, 1H), 4.15 (s, 2H), 2.81 (d, *J* = 4.8 Hz, 3H). ^13^C NMR (100 MHz, CDCl_3_) δ(ppm): 170.8, 140.9, 138.9, 136.8, 130.9, 129.9, 129.0, 128.4, 127.1, 126.3, 126.1, 38.9, 26.5.

*Methyl 2-(4-(methylcarbamoyl)benzyl)benzoate* (**3ah**) [45] White solid (40.1 mg, 14%); m.p. = 139–141 °C. ^1^H NMR (400 MHz, CDCl_3_) δ(ppm): 7.91 (d, *J* = 8.4 Hz, 2H), 7.35–7.32 (m, 2H), 7.24–7.19 (m, 4H), 5.69 (s, 1H), 4.22 (s, 2H), 3.87 (s, 3H), 2.84 (d, *J* = 4.8 Hz, 3H). ^13^C NMR (100 MHz, CDCl_3_) δ(ppm): 170.6, 167.2, 146.5, 138.4, 136.7, 131.1, 130.2, 129.8, 129.1, 128.1, 127.1, 126.7, 52.1, 39.0, 26.7.

*2-(2-Ethylhexyl)-N-methylbenzamide* (**3aj**) [43] Colorless oil (81.3 mg, 33%; 189.0 mg, 76% at 80 °C). ^1^H NMR (400 MHz, CDCl_3_) δ(ppm): 7.32–7.27 (m, 2H), 7.20–7.15 (m, 2H), 5.81 (s, 1H), 2.95 (d, *J* = 4.8 Hz, 3H), 2.76–2.67 (m, 2H), 1.57–1.51 (m, 1H), 1.29–1.19 (m, 8H), 0.88–0.81 (m, 6H). ^13^C NMR (100 MHz, CDCl_3_) δ(ppm): 171.2, 139.9, 137.1, 130.8, 129.3, 126.8, 125.6, 40.9, 37.2, 32.5, 28.8, 26.5, 25.5, 23.0, 14.1, 10.7.

*2-(sec-Butyl)-N-methylbenzamide* (**3ak**) [43] White solid (59.7 mg, 31%; 126.0 mg, 66% at 80 °C); m.p. = 77–79 °C. ^1^H NMR (400 MHz, CDCl_3_) δ(ppm): 7.37–7.33 (m, 1H), 7.30–7.23 (m, 2H), 7.17–7.13 (m, 1H), 5.88 (s, 1H), 3.06–2.97 (m, 1H), 2.94 (d, *J* = 4.8 Hz, 3H), 1.67–1.54 (m, 2H), 1.22 (d, *J* = 6.8 Hz, 3H), 0.81 (t, *J* = 7.2 Hz, 3H). ^13^C NMR (100 MHz, CDCl_3_) δ(ppm): 171.3, 145.5, 137.0, 129.8, 126.5, 126.3, 125.5, 37.0, 31.0, 26.6, 22.2, 12.3.

*2-iso-Propyl-N-methylbenzamide* (**3al**) [61] White solid (62.1 mg, 35%; 130.8 mg, 74% at 80 °C); m.p. = 91–93 °C. ^1^H NMR (400 MHz, CDCl_3_) δ(ppm): 7.38–7.33 (m, 2H), 7.27–7.23 (m, 1H), 7.18–7.14 (m, 1H), 5.88 (s, 1H), 3.34–3.27(m, 1H), 2.95 (d, *J* = 4.8 Hz, 3H), 1.24 (d, *J* = 6.8 Hz, 6H). ^13^C NMR (100 MHz, CDCl_3_) δ(ppm): 171.3, 146.5, 136.2, 129.8, 126.5, 125.9, 125.5, 29.9, 26.5, 24.1.

*2-Ethyl-N-propylbenzamide* (**3bb**) [62] Colorless oil (171.5 mg, 90%). ^1^H NMR (400 MHz, CDCl_3_) δ(ppm): 7.34–7.28 (m, 2H), 7.24 (d, *J* = 7.2 Hz, 1H), 7.19–7.15 (m, 1H), 5.91 (s, 1H), 3.39–3.34 (m, 2H), 2.78 (q, *J* = 7.6 Hz, 2H), 1.66–1.56 (m, 2H), 1.23 (t, *J* = 7.6 Hz, 3H), 0.97 (t, *J* = 7.2 Hz, 3H). ^13^C NMR (100 MHz, CDCl_3_) δ(ppm): 170.4, 142.0, 136.6, 129.7, 129.2, 126.6, 125.6, 41.5, 26.2, 22.8, 15.8, 11.4.

*2-Ethyl-N-isopropylbenzamide* (**3cb**) [63] White solid (170.3 mg, 89%); m.p. = 74–75 °C. ^1^H NMR (400 MHz, CDCl_3_) δ(ppm): 7.34–7.28 (m, 2H), 7.24 (d, *J* = 7.2 Hz, 1H), 7.18 (t, *J* = 7.2 Hz, 1H), 5.63 (s, 1H), 4.31–4.22 (m, 1H), 2.79 (q, *J* = 7.6 Hz, 2H), 1.25–1.21 (m, 9H). ^13^C NMR (100 MHz, CDCl_3_) δ(ppm): 169.5, 142.0, 136.7, 129.7, 129.3, 126.6, 125.6, 41.7, 26.3, 22.7, 15.8.

*N-Cyclohexyl-2-ethylbenzamide* (**3db**) [62] White solid (208.2 mg, 90%); m.p. = 134–136 °C. ^1^H NMR (400 MHz, CDCl_3_) δ(ppm): 7.34–7.28 (m, 2H), 7.24 (d, *J* = 7.6 Hz, 1H), 7.18 (t, *J* = 7.2 Hz, 1H), 5.67 (d, *J* = 6.0 Hz, 1H), 4.01–3.92 (m, 1H), 2.79 (q, *J* = 7.6 Hz, 2H), 2.05–2.01 (m, 2H), 1.76–1.71 (m, 2H), 1.67–1.63 (m, 1H), 1.48–1.37 (m, 2H), 1.25–1.16 (m, 6H). ^13^C NMR (100 MHz, CDCl_3_) δ(ppm): 169.4, 142.0, 136.8, 129.7, 129.3, 126.6, 125.6, 48.5, 33.1, 26.3, 25.5, 24.9, 15.8.

*2-Ethyl-N-(2-methoxyethyl)benzamide* (**3eb**) Yellow oil (181.0 mg, 87%). ^1^H NMR (400 MHz, CDCl_3_) δ(ppm): 7.35–7.32 (m, 2H), 7.27–7.24 (m, 1H), 7.19 (t, *J* = 7.6 Hz, 1H), 6.22 (s, 1H), 3.63–3.59 (m, 2H), 3.56–3.53 (m, 2H), 3.36 (s, 3H), 2.80 (q, *J* = 7.6 Hz, 2H), 1.24 (t, *J* = 7.6 Hz, 3H). ^13^C NMR (100 MHz, CDCl_3_) δ(ppm): 170.3, 142.0, 136.2, 129.8, 129.3, 126.7, 125.6, 71.0, 58.6, 39.4, 26.2, 15.8. HRMS (ESI) *m*/*z*: [M + H]^+^ calcd for C_12_H_18_NO_2_ 208.1338; found 208.1337.

*N-Benzyl-2-ethylbenzamide* (**3fb**) [60] White solid (218.0 mg, 91%); m.p. = 94–96 °C. ^1^H NMR (400 MHz, CDCl_3_) δ(ppm): 7.35–7.24 (m, 8H), 7.17 (t, *J* = 7.6 Hz, 1H), 6.12 (s, 1H), 4.59 (d, *J* = 5.6 Hz, 2H), 2.80 (q, *J* = 7.6 Hz, 2H), 1.22 (t, *J* = 7.6 Hz, 3H). ^13^C NMR (100 MHz, CDCl_3_) δ(ppm): 170.1, 142.4, 138.3, 136.0, 130.0, 129.4, 128.7, 127.8, 127.5, 126.7, 125.7, 43.8, 26.3, 15.9.

*2-Ethyl-N-(4-formylbenzyl)benzamide* (**3gb**) White solid (63.2 mg, 24%); m.p. = 109–111 °C. ^1^H NMR (400 MHz, CDCl_3_) δ(ppm): 9.96 (s, 1H), 7.83 (d, *J* = 8.0 Hz, 2H), 7.49 (d, *J* = 8.0 Hz, 2H), 7.37–7.32 (m, 2H), 7.27–7.25 (m, 1H), 7.18 (t, *J* = 7.2 Hz, 1H), 6.45 (s, 1H), 4.65 (d, *J* = 6.0 Hz, 2H), 2.78 (q, *J* = 7.6 Hz, 2H), 1.20 (t, *J* = 7.6 Hz, 3H). ^13^C NMR (100 MHz, CDCl_3_) δ(ppm): 191.9, 170.4, 145.6, 142.3, 135.6, 135.4, 130.1, 130.0, 129.4, 128.0, 126.7, 125.6, 43.3, 26.2, 15.8. HRMS (ESI) *m*/*z*: [M + Na]^+^ calcd for C_17_H_17_NO_2_Na 290.1157; found 290.1156.

*N-(4-Cyanobenzyl)-2-ethylbenzamide* (**3hb**) White solid (189.9 mg, 72%); m.p. = 110–111 °C. ^1^H NMR (400 MHz, CDCl_3_) δ(ppm): 7.61 (d, *J* = 8.0 Hz, 2H), 7.44 (d, *J* = 8.0 Hz, 2H), 7.38–7.32 (m, 2H), 7.27–7.25 (m, 1H), 7.19 (t, *J* = 7.6 Hz, 1H), 6.41 (s, 1H), 4.63 (d, *J* = 6.0 Hz, 2H), 2.78 (q, *J* = 7.6 Hz, 2H), 1.20 (t, *J* = 7.6 Hz, 3H).^13^C NMR (100 MHz, CDCl_3_) δ(ppm): 170.4, 144.1, 142.5, 135.4, 132.4, 130.3, 129.6, 128.2, 126.7, 125.8, 118.8, 111.1, 43.2, 26.3, 15.9. HRMS (ESI) *m*/*z*: [M + Na]^+^ calcd for C_17_H_16_N_2_ONa 287.1160; found 287.1161.

*2-Ethyl-N-(2,2,2-trifluoroethyl)benzamide* (**3ib**) White solid (50.9 mg, 22%); m.p. = 68–69 °C. ^1^H NMR (400 MHz, CDCl_3_) δ(ppm): 7.39–7.35 (m, 1H), 7.32–7.26 (m, 2H), 7.20 (t, *J* = 7.6 Hz, 1H), 6.22 (s, 1H), 4.09–4.01 (m, 2H), 2.76 (q, *J* = 7.6 Hz, 2H), 1.21 (t, *J* = 7.6 Hz, 3H). ^13^C NMR (100 MHz, CDCl_3_) δ(ppm): 170.3, 142.8, 134.9, 130.7, 129.8, 126.8, 125.9, 124.2 (q, *J* = 277.0 Hz), 40.9 (q, *J* = 34.0 Hz), 26.4, 15.9. ^19^F NMR (400 MHz, CDCl_3_) δ(ppm): −72.41 (t, *J* = 9.6 Hz). HRMS (ESI) *m*/*z*: [M + Na]^+^ calcd for C_11_H_12_NOF_3_Na 254.0769; found 254.0767.

*Ethyl (2-ethylbenzoyl)glycinate* (**3jb**) White solid (97.6 mg, 41%); m.p. = 49–51 °C. ^1^H NMR (400 MHz, CDCl_3_) δ(ppm): 7.40–7.34 (m 2H), 7.28–7.26 (m, 1H), 7.21 (t, *J* = 7.2 Hz, 1H), 6.34 (s, 1H), 4.24 (q, *J* = 7.2 Hz, 2H), 4.20 (d, *J* = 5.2 Hz, 2H), 2.82 (q, *J* = 7.6 Hz, 2H), 1.31 (t, *J* = 7.2 Hz, 3H), 1.24 (t, *J* = 7.6 Hz, 3H). ^13^C NMR (100 MHz, CDCl_3_) δ(ppm): 170.4, 169.9, 142.6, 135.4, 130.3, 129.5, 127.0, 125.8, 61.6, 41.7, 26.3, 15.9, 14.2. HRMS (ESI) *m*/*z*: [M + Na]^+^ calcd for C_13_H_17_NO_3_Na 258.1106; found 258.1108.

*2-Ethyl-N-phenylbenzamide* (**3kb**) [62] White solid (24.8 mg, 11%); m.p. = 141–142 °C. ^1^H NMR (400 MHz, CDCl_3_) δ(ppm): 7.63–7.58 (m, 3H), 7.42–7.32(m, 4H), 7.30–7.28 (m, 1H), 7.24–7.20 (m, 1H), 7.13 (t, *J* = 7.2 Hz, 1H), 2.83 (q, *J* = 7.6 Hz, 2H), 1.24 (t, *J* = 7.6 Hz, 3H).^13^C NMR (100 MHz, CDCl_3_) δ(ppm): 168.4, 142.6, 138.1, 136.3, 130.4, 129.7, 129.1, 126.7, 125.9, 124.6, 120.0, 26.4, 15.9.

*Methyl 3-ethyl-4-(methylcarbamoyl)benzoate* (**3lb**) White solid (135.1 mg, 61%); m.p. = 119–121 °C. ^1^H NMR (400 MHz, CDCl_3_) δ(ppm): 7.90 (s, 1H), 7.81 (d, *J* = 8.0 Hz, 1H), 7.33 (d, *J* = 7.6 Hz, 1H), 6.07 (s, 1H), 3.91 (s, 3H), 2.97 (d, *J* = 4.8 Hz, 3H), 2.79 (q, *J* = 7.6 Hz, 2H), 1.24 (t, *J* = 7.6 Hz, 3H).^13^C NMR (100 MHz, CDCl_3_) δ(ppm): 170.2, 166.7, 142.4, 140.5, 131.1, 130.3, 126.9, 126.8, 52.2, 26.6, 26.2, 15.5. HRMS (ESI) *m*/*z*: [M + Na]^+^ calcd for C_12_H_15_NO_3_Na 244.0950; found 244.0952.

*2-Ethyl-5-fluoro-N-methylbenzamide* (**3mb**) White solid (159.4 mg, 88%); m.p. = 92–93 °C. ^1^H NMR (400 MHz, CDCl_3_) δ(ppm): 7.21–7.17 (m, 1H), 7.04–6.96 (m, 2H), 6.05 (s, 1H), 2.94 (d, *J* = 4.8 Hz, 3H), 2.71 (q, *J* = 7.6 Hz, 2H), 1.18 (t, *J* = 7.6 Hz, 3H). ^13^C NMR (100 MHz, CDCl_3_) δ(ppm): 169.8, 160.5 (d, *J* = 244.0 Hz), 137.8 (d, *J* = 3.0 Hz), 137.6 (d, *J* = 6.0 Hz), 130.9 (d, *J* = 8.0 Hz), 116.5 (d, *J* = 21.0 Hz), 113.7(d, *J* = 23.0 Hz), 26.6, 25.6, 15.8. ^19^F NMR (400 MHz, CDCl_3_) δ(ppm): −117.14 (m). HRMS (ESI) *m*/*z*: [M + H]^+^ calcd for C_10_H_13_NOF 182.0981; found 182.0982.

*5-Chloro-2-ethyl-N-methylbenzamide* (**3nb**) White solid (158.3 mg, 80%); m.p. = 120–121 °C. ^1^H NMR (400 MHz, CDCl_3_) δ(ppm): 7.30–7.25 (m, 2H), 7.17 (d, *J* = 8.4 Hz, 1H), 6.03 (s, 1H), 2.94 (d, *J* = 4.8 Hz, 3H), 2.72 (q, *J* = 7.6 Hz, 2H), 1.18 (t, *J* = 7.6 Hz, 3H). ^13^C NMR (100 MHz, CDCl_3_) δ(ppm): 169.6, 140.7, 137.7, 131.2, 130.8, 129.8, 126.8, 26.6, 25.8, 15.6. HRMS (ESI) *m*/*z*: [M + H]^+^ calcd for C_10_H_13_NOCl 198.0686; found 198.0689.

*2-Ethyl-N,5-dimethylbenzamide* (**3pb**) White solid (134.8 mg, 76%; 151.2 mg, 85% using 5 mol% NiCl_2_(DME)/10 mol% bpy); m.p. = 120–122 °C. ^1^H NMR (400 MHz, CDCl_3_) δ(ppm): 7.13–7.11 (m, 3H), 5.88 (s, 1H), 2.95 (d, *J* = 4.4 Hz, 3H), 2.73 (q, *J* = 7.6 Hz, 2H), 2.30 (s, 3H), 1.19 (t, *J* = 7.6 Hz, 3H). ^13^C NMR (100 MHz, CDCl_3_) δ(ppm): 171.2, 139.1, 136.2, 135.1, 130.5, 129.2, 127.4, 26.5, 25.9, 20.8, 15.9. HRMS (ESI) *m*/*z*: [M + H]^+^ calcd for C_11_H_16_NO 178.1232; found 178.1233.

*2-Ethyl-5-methoxy-N-methylbenzamide* (**3qb**) White solid (147.3 mg, 76%; 169.6 mg, 88% using 5 mol% NiCl_2_(DME)/10 mol% bpy); m.p. = 108–110 °C. ^1^H NMR (400 MHz, CDCl_3_) δ(ppm): 7.14 (d, *J* = 8.4 Hz, 1H), 6.88–6.83 (m, 2H), 5.92 (s, 1H), 3.77 (s, 3H), 2.95 (d, *J* = 4.8 Hz, 3H), 2.69 (q, *J* = 7.6 Hz, 2H), 1.18 (t, *J* = 7.6 Hz, 3H). ^13^C NMR (100 MHz, CDCl_3_) δ(ppm): 170.8, 157.4, 137.1, 134.0, 130.4, 115.5, 112.2, 55.4, 26.6, 25.5, 16.0. HRMS (ESI) *m*/*z*: [M + H]^+^ calcd for C_11_H_16_NO_2_ 194.1181; found 194.1182.

*2-Ethyl-3-methoxy-N-methylbenzamide* (**3rb**) White solid (176.2 mg, 91%); m.p. = 109–112 °C. ^1^H NMR (400 MHz, CDCl_3_) δ(ppm): 7.14 (t, *J* = 8.0 Hz, 1H), 6.89–6.87 (m, 2H), 5.86 (s, 1H), 3.83(s, 3H), 2.94 (d, *J* = 4.8 Hz, 3H), 2.71 (q, *J* = 7.6 Hz, 2H), 1.15 (t, *J* = 7.6 Hz, 3H). ^13^C NMR (100 MHz, CDCl_3_) δ(ppm): 171.0, 157.7, 137.9, 130.6, 126.6, 118.8, 111.5, 55.5, 26.4, 20.5, 14.8. HRMS (ESI) *m*/*z*: [M + H]^+^ calcd for C_11_H_16_NO_2_ 194.1181; found 194.1182.

*2-Ethyl-N-methyl-[1,1′-biphenyl]-3-carboxamide* (**3sb**) White solid (201.2 mg, 84%); m.p. = 134–136 °C. ^1^H NMR (400 MHz, CDCl_3_) δ(ppm): 7.41–7.35 (m, 3H), 7.29–7.26 (m, 3H), 7.21–7.20 (m, 2H), 5.96 (s, 1H), 2.98 (d, *J* = 4.8 Hz, 3H), 2.73 (q, *J* = 7.6 Hz, 2H), 0.96 (t, *J* = 7.6 Hz, 3H). ^13^C NMR (100 MHz, CDCl_3_) δ(ppm): 171.6, 143.1, 141.6, 139.6, 137.4, 131.6, 129.1, 128.1, 127.0, 126.2, 125.4, 26.6, 23.2, 15.9. HRMS (ESI) *m*/*z*: [M + H]^+^ calcd for C_16_H_18_NO 240.1388; found 240.1387.

*2-Ethyl-N,3-dimethylbenzamide* (**3tb**) White solid (153.1 mg, 86%); m.p. = 72–74 °C. ^1^H NMR (400 MHz, CDCl_3_) δ(ppm): 7.18–7.16 (m, 1H), 7.11–7.04 (m, 2H), 5.87 (s, 1H), 2.95 (d, *J* = 4.8 Hz, 3H), 2.73 (q, *J* = 7.6 Hz, 2H), 2.34 (s, 3H), 1.16 (t, *J* = 7.6 Hz, 3H). ^13^C NMR (100 MHz, CDCl_3_) δ(ppm): 171.7, 140.0, 137.1, 136.8, 131.5, 125.4, 124.5, 26.4, 23.3, 19.2, 14.8. HRMS (ESI) *m*/*z*: [M + H]^+^ calcd for C_11_H_16_NO 178.1232; found 178.1234.

### 3.3. Procedure for Gram-Scale Synthesis of 2-Ethyl-N,3-Dimethylbenzamide (***3tb***)

To a 100 mL dry flask, 3,8-dimethylbenzotriazinone (**1t**, 1.752 g, 10.0 mmol, 1.0 equiv.), NiCl_2_(DME) (0.110 g, 0.5 mmol, 5 mol%), bpy (0.078 g, 0.5 mmol, 5 mol%), TBAI (0.110 g, 0.3 mmol, 3 mol%), and Mn powder (1.100 g, 20.0 mmol, 2.0 equiv.) were added. After replacement of the air in the flask with N_2_ using a standard Schlenk line, non-anhydrous DMA (dried over 4 Å MS, 30 mL), ethyl tosylate (**2b**, 3.000 g, 15.0 mmol, 1.5 equiv.), and TMSCl (130 μL, 1.0 mmol, 10 mol%) were added by syringe. The mixture was stirred at room temperature for 30 min and then heated at 60 °C (oil bath) for 8 h. After being cooled to room temperature, 5% HCl (20 mL) was added to quench the reaction. The mixture was filtered to remove insoluble solids. The filtrate was diluted and extracted with ethyl acetate (3 × 50 mL). The combined organic phase was dried over Na_2_SO_4_, filtered, and evaporated by rotavapor to give the crude product, which was purified by column chromatography on silica gel with petroleum ether/ethyl acetate (3:1) eluent to afford product **3tb** 1.478 g (84%).

## 4. Conclusions

In summary, an efficient denitrogenative XEC of *N*-alkyl-1,2,3-benzotriazinones with alkyl tosylates and mesylates has been effected in inexpensive non-anhydrous DMA by using 5 mol% NiCl_2_(DME)/bpy catalyst in the presence of 3 mol% TBAI co-catalyst to convert sulfonates into iodides in situ. The stabilizing effect of the electron-neutral/-rich *N*-alkyl groups in benzotriazinones and the in situ conversion of alkyl sulfonates into iodides have been proposed to be the key for the compatibility to water residue in non-anhydrous DMA solvent. An optimal condition has been established, under which the scope and limitations of the protocol have been briefly explored, showing a large electronic effect from the *N*-substituents in benzotriazinones while their steric hindrance could be well tolerated. In fact, an unexpected steric acceleration has been observed from the core of benzotriazinones, not only promising highly efficient access to 2-alkyl-2,3-disubstituted benzamides from readily accessible substrates but also implying sterically switchable rate-determining steps in the catalytic cycle. Mesylates appeared to be as usable as tosylates in the iodide/nickel co-catalyzed XEC with benzotriazinones and even better in the case of methylation. In addition, good tolerance to up to 1000 ppm water content in DMA solvent makes the protocol more practical and economical, in particular if conducted at scale.

## Figures and Tables

**Table 1:** Optimization of conditions for the model reaction ^a^.

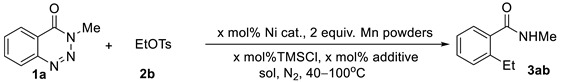							
Entry	Cat. (mol%)	mol% TMSCl	Add. (mol%)	Sol.	T (℃)	Time (h)	Yield (%) ^b^
1	NiCl_2_(bpy) (10)	25	KI (10)	DMA	100	2	76
2	NiCl_2_(bpy) (10)	25	NaI (10)	DMA	100	2	74
3	NiCl_2_(bpy) (10)	25	/	DMA	100	4	20
4	NiCl_2_(bpy) (10)	25	KBr (10)	DMA	100	4	36
5	NiCl_2_(bpy) (10)	25	TBAB (10)	DMA	100	4	41
6	NiCl_2_(bpy) (10)	25	TBAI (10)	DMA	100	1	86
7	NiCl_2_(bpy) (10)	25	TBAI (3)	DMA	100	1	83
8	NiCl_2_(bpy) (10)	25	TBAI (1)	DMA	100	3	77
9	NiCl_2_(bpy) (10)	25	TBAI (3)	DMA	80	2	85
10	NiCl_2_(bpy) (10)	25	TBAI (3)	DMA	60	4	86
11	NiCl_2_(bpy) (10)	25	TBAI (3)	DMA	40	10	56 ^c^
12	NiCl_2_(bpy) (5)	25	TBAI (3)	DMA	60	10	86
13	NiCl_2_(bpy) (3)	25	TBAI (3)	DMA	60	14	74 ^c^
14	NiCl_2_/bpy (5)	25	TBAI (3)	DMA	60	10	85
15	Ni(OAc)_2_/bpy (5)	25	TBAI (3)	DMA	60	12	70 ^c^
16	Ni(acac)_2_/bpy (5)	25	TBAI (3)	DMA	60	12	68 ^c^
17	NiCl_2_(DME)/bpy (5)	25	TBAI (3)	DMA	60	10	89
18	NiCl_2_(PPh_3_)_2_(5) ^d^	25	TBAI (3)	DMA	60	14	trace
19	NiCl_2_(DME)/bpy (3)	25	TBAI (3)	DMA	60	14	85
20	NiCl_2_(DME)/dtbbpy(3)	25	TBAI (3)	DMA	60	14	26 ^c^
21	NiCl_2_(DME)/phen (3)	25	TBAI (3)	DMA	60	14	71 ^c^
22	NiCl_2_(DME)/bpy (5)	25	TBAI (3)	DMF	60	12	74
23	NiCl_2_(DME)/bpy (5)	25	TBAI (3)	Sol.^e^	60	12	NR
24	NiCl_2_(DME)/bpy (5)	50	TBAI (3)	DMA	60	8	65 ^c^
25	NiCl_2_(DME)/bpy (5)	10	TBAI (3)	DMA	60	10	88
26	NiCl_2_(DME)/bpy (5)	5	TBAI (3)	DMA	60	12	83 ^c^
27	NiCl_2_(DME)/bpy (5)	/	TBAI (3)	DMA	60	12	36 ^c^
28	NiCl_2_(DME)/bpy (5)	10	TBAI (3)	DMA	60	10	72 ^c,f^
29	NiCl_2_(DME)/bpy (5)	10	TBAI (3)	DMA	60	8	89 ^g^
30	NiCl_2_(DME)/bpy (5)	10	TBAI (3)	DMA	60	10	86 ^h^
31	NiCl_2_(DME)/bpy (5)	10	TBAI (3)	DMA	60	10	81 ^i^
32	NiCl_2_(DME)/bpy (5)	10	TBAI (3)	DMA	60	8	NR ^j^

^a^ Reaction run at 1 mmol scale with respect to **1a** with 1.5 equiv. **2b**, in 3 mL non-anhydrous DMA dried over 4 Å MS. ^b^ Isolated yield. ^c^ Incomplete conversion of **1a**. ^d^ Similarly, no reaction observed with NiCl_2_ or NiCl_2_(DME) without supporting ligand. ^e^ DMSO, THF, DME, dioxane, or toluene as solvent. ^f^ With 1.0 equiv. **2b**. ^g^ With 2.0 equiv. **2b**. ^h^ Non-anhydrous DMA without drying over MS. ^i^ DMA containing 1000 ppm (0.1% *w*/*w*) H_2_O. ^j^ DMA containing 5000 ppm (0.5% *w*/*w*) H_2_O.

## Data Availability

Data are reported in this paper and the Supplementary Materials.

## Supplementary Materials

The following Supplementary Materials can be downloaded at https://www.mdpi.com/article/10.3390/molecules30112397/s1: Copies of ^1^H & ^13^C NMR spectra of products; HRMS for all new compounds and GC-MS data mentioned in the text.
