# Supplementary material for: Iodide/Nickel Co-Catalyzed Manganese-Mediated Denitrogenative Cross-Electrophile Coupling of Benzotriazinones with Alkyl Sulfonates
Source: Molecules. 2025 May 30;30(11):2397. doi: 10.3390/molecules30112397 (PMC12156213; doi:10.3390/molecules30112397)

Supplementary Materials

For

Iodide/nickel co-catalyzed manganese-mediated denitrogenative cross-electrophile coupling of benzotriazinones with alkyl sulfonates

Yingying Hong, Xuanxuan Zhang and Gang Zou*

Table of Contents

1. Characterization data of benzotriazinones 1

2. ^{1}H and ^{13}C NMR spectra 5

3. ^{19}F NMR spectra 38

4. ^{1}H NMR and GC-MS of crude product with 2a 39

5. ^{1}H NMR and GC-MS of the XEC product (Cl/Br/I scrambled) with 2e 43

6.GC-MS of 3ah 46

7.GC-MS of the reaction mixture from 1a with 2i 48

8. GC-MS of the reaction mixture from the competing reaction 50

9. ^{1}H NMR and GC-MS of radical clock experiment 54

10. HRMS of new compounds 58

1. Characterization data of benzotriazinones

All starting materials of benzotriazinones were shown in Figure S1.

Method a [1] (except for benzotriazinones 1c, 1e-h, 1j)

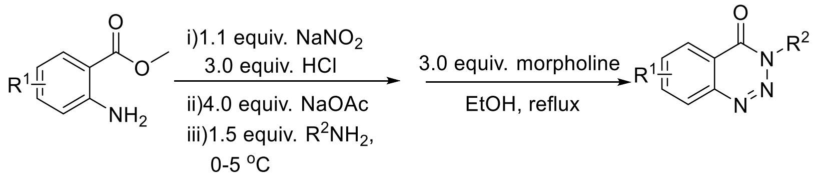

To a 100 mL round-bottom flask was added methyl anthranilate derivative (20 mmol, 1.0 equiv.) in HCl (con., 5.0 mL, 60 mmol) followed by solution of $NaNO_2$ (1.518 g, 22 mmol, 1.1 equiv.) in water (10 mL) at 0 °C. The resulting solution was stirred for 1 h. Then, a solution of NaOAc (6.562 g, 80 mmol, 4.0 equiv.) in water (20 mL) was slowly added and followed by addition of amine (30 mmol, 1.5 equiv.) at 0 °C. The resulting mixture was stirred at 0 °C for 6 h. The precipitate was collected by filtration, washed with water (70 mL), and purified by recrystallization from ethanol to afford triazenes.

The above triazene was refluxed in ethanol (70 mL) with excess morpholine (5.228 g, 60 mmol, 3.0 equiv.) until triazene was completely consumed (TLC). The reaction mixture was cooled to room temperature to afford N-alkyl benzotriazinone as crystals.

Preparation of 1s

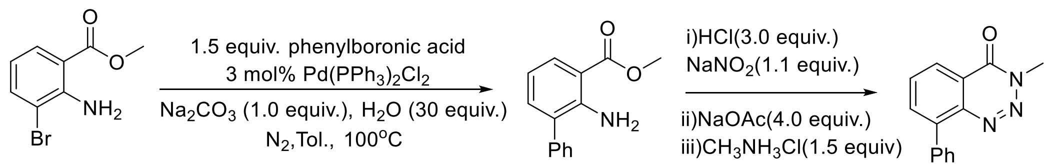

A 100 mL reaction tube containing methyl 2-amino-3-bromobenzoate (20 mmol, 4.600 g), phenylboronic acid (30 mmol, 3.600 g), $PdCl_2(PPh_3)_2$ (0.6 mmol, 0.420 g), Na_2CO_3 (20 mmol, 2.120 g) was evacuated and purged with nitrogen three times. Then, toluene (40 mL) and H_2O (600 mmol, 10.8 mL) were added by syringe and the reaction mixture was stirred at 100 °C (bath temperature). After complete consumption of methyl 2-amino-3-bromobenzoate, as monitored by TLC, the mixture was cooled to room temperature, diluted and extracted with ethyl acetate (3 × 100 mL). The combined organic phase was dried over Na_2SO_4, filtered, and evaporated by rotavapor to give the crude product, which was purified by column chromatography on silica gel with petroleum ether/ethyl acetate eluent to afford the product methyl 2-amino-[1,1'-biphenyl]-3-carboxylate (19 mmol, 4.320 g).

Then, to a 100 mL round-bottom flask was added methyl 2-amino-[1,1'-biphenyl]-3-carboxylate (19 mmol, 4.320 g) in HCl (conc., 57 mmol, 4.8 mL) followed by a solution of $NaNO_2$ (1.442 g, 20.9 mmol) in water (10 mL) at 0 °C. The resulting solution was stirred for 60 min. Then, a solution of NaOAc (76 mmol, 6.232 g) in water (20 mL) was slowly added and followed by the addition of methylamine hydrochloride (28.5 mmol, 1.924 g) at 0 °C . The resulting mixture was stirred at 0 °C for 6 h. The precipitate was collected by filtration, washed with water (50 mL), and purified by recrystallization from ethanol to afford benzotriazinone **1s** (3.420 g, 14.4 mmol).

Method b[2] (benzotriazinones 1c, 1e-h and 1j)

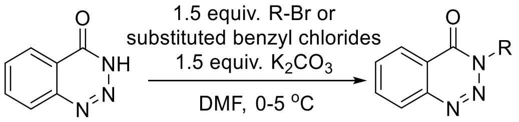

To a 100 mL three-necked flask was added 1,2,3-Benzotriazin-4(3H)-one (2.940 g, 20 mmol) and K_2CO_3 (4.146 g, 30 mmol, 1.5 equiv.). After replacement of air in the flask by N_2 using a standard Schlenk line, DMF (40 mL) and the corresponding alkyl bromide or benzyl chloride (30 mmol, 1.5 equiv.) were added via syringe at 0-5 °C. The mixture stirred until complete consumption of starting material (TLC). Then, diluted and extracted with ethyl acetate (3 × 100 mL). The combined organic phase was dried over Na_2SO_4, filtered, and evaporated by rotavapor to give the crude product. The crude product was purified by recrystallization from ethanol to afford pure benzotriazinone.

New compounds **1g**, **1h**, **1o, 1p, 1s** were characterized by both NMR spectra and HRMS.

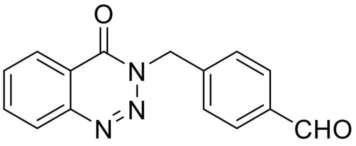

1g

4-((4-oxobenzo[d][1,2,3]triazin-3(4H)-yl)methyl)benzaldehyde (1g) White solid; m.p. = 177-179 °C. **^{1}H NMR** (400 MHz, $CDCl_3$) δ(ppm): 9.99 (s, 1H), 8.34 (d, J = 8.0 Hz, 1H), 8.17 (d, J = 8.4 Hz, 1H), 7.98 - 7.94 (m, 1H), 7.86 (d, J = 8.0 Hz, 2H), 7.83 - 7.79 (m, 1H), 7.66 (d, J = 8.0 Hz, 2H), 5.69 (s, 2H). **^{13}C NMR** (100 MHz, $CDCl_3$) δ(ppm) 191.8, 155.4, 144.3, 142.3, 136.2, 135.1, 132.7, 130.2, 129.3, 128.5, 125.2, 120.0, 53.0. **HRMS** (ESI) m/z: $[M+Na]^+$ calcd for $C_{15}H_{11}N_3O_2Na$ 288.0749; found 288.0750.

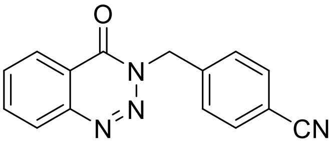

1h

4-((4-oxobenzo[d][1,2,3]triazin-3(4H)-yl)methyl)benzonitrile (1h) White solid, m.p. = 196-198 °C. **^{1}H NMR** (400 MHz, $CDCl_3$) δ(ppm) 8.34 (d, J = 8.0 Hz, 1H), 8.18 (d, J = 8.0 Hz, 1H), 7.99 - 7.95 (m, 1H), 7.84 - 7.80 (m, 1H), 7.66 - 7.61 (m, 4H), 5.66 (s, 2H). **^{13}C NMR** (100 MHz, $CDCl_3$) δ(ppm) 155.4, 144.3, 140.8, 135.2, 132.8 132.6, 129.5, 128.6, 125.2, 119.9, 118.5, 112.2, 52.9. **HRMS** (ESI) m/z: $[M+H]^+$ calcd for $C_{15}H_{11}N_4O$ 263.0933; found 263.0931.

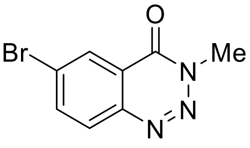

1o

6-bromo-3-methylbenzo[d][1,2,3]triazin-4(3H)-one(1o) Yellow solid; m.p. = 145-146 °C. **^{1}H NMR** (400 MHz, $CDCl_3$) δ(ppm): 8.45 (s, 1H), 8.02 - 7.97 (m, 2H), 4.04 (s, 3H). **^{13}C NMR** (100 MHz, $CDCl_3$) δ(ppm): 154.5, 143.1, 138.1, 129.9, 127.6, 126.9, 120.8, 37.5. **HRMS** (ESI) m/z: $[M+H]^+$ calcd for $C_8H_7BrN_3O$ 239.9772, found 239.9777.

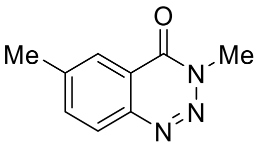

1p

3,6-dimethylbenzo[d][1,2,3]triazin-4(3H)-one(1p) White solid, m.p. = 88-90 °C. **^{1}H NMR** (400 MHz, $CDCl_3$) δ(ppm): 8.10 (s, 1H), 8.00 (d, J = 8.4 Hz, 1H), 7.71 (dd, J = 8.0, 1.6 Hz, 1H), 4.02 (s, 3H), 2.55 (s, 3H). **^{13}C NMR** (100 MHz, $CDCl_3$) δ(ppm): 155.9, 143.5, 142.9, 136.1, 128.1, 124.2, 119.5, 37.3, 21.9. **HRMS** (ESI) m/z: $[M+H]^+$ calcd for $C_9H_{10}N_3O$ 176.0824, found 176.0823.

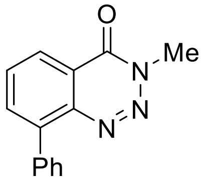

1s

3-methyl-8-phenylbenzo[d][1,2,3]triazin-4(3H)-one (1s) White solid, m.p. = 201-203 °C. **^{1}H NMR** (400 MHz, $CDCl_3$) δ(ppm) 8.37 (d, J = 8.0 Hz, 1H), 7.94 (d, J = 7.2 Hz, 1H), 7.83 (t, J = 7.6 Hz, 1H), 7.63 (d, J = 7.6 Hz, 2H), 7.52 -7.43 (m, 3H), 4.06 (s, 3H). **^{13}C NMR** (100 MHz, $CDCl_3$) δ(ppm) 155.9, 141.5, 141.0, 136.7, 135.8, 132.2, 130.7, 128.3, 128.2, 124.1, 120.1, 37.3. **HRMS** (ESI) m/z: $[M+H]^+$ calcd for $C_{14}H_{12}N_3O$ 238.0980; found 238.0982.

References

1. Wang, F.; Tong, Y.; Zou, G. Nickel-catalyzed, manganese-assisted denitrogenative cross-electrophile-coupling of benzotriazinones with alkyl halides for ortho-alkylated benzamides. *Org. Lett.* **2022**, *24*, 5741-5745, doi:10.1021/acs.orglett.2c02182.
2. Lin, T.; Wang, Y.-E.; Cui, N.; Li, M.; Wang, R.; Bai, J.; Fan, Y.; Xiong, D.; Xue, F.; Walsh, P.J.; et al. Nickel-catalyzed cross-electrophile coupling of 1,2,3-Benzotriazin-4(3*H*)-ones with aryl bromides. *J. Org. Chem.* **2022**, *87*, 16567-16577, doi:10.1021/acs.joc.2c02246

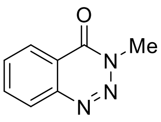
1a

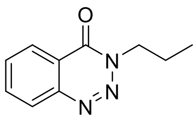
1b

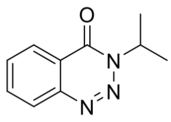
1c

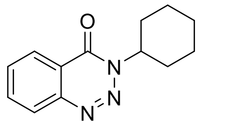
1d

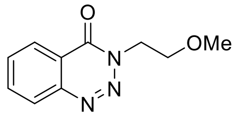
1e

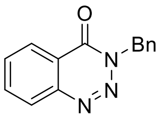
1f

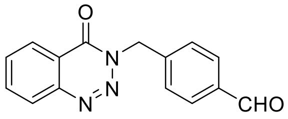
1g

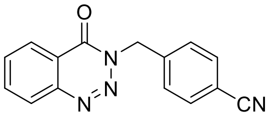
1h

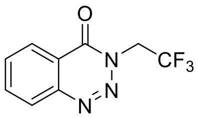
1i

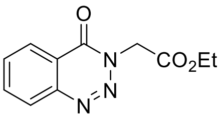
1j

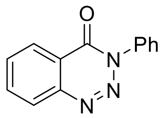
1k

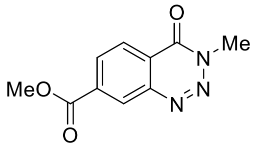
1l

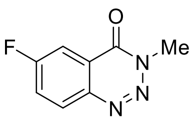
1m

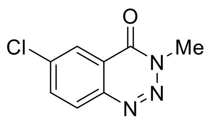
1n

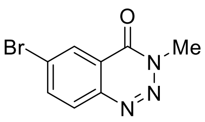
1o

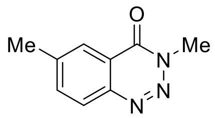
1p

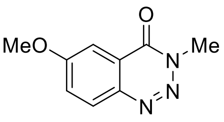
1q

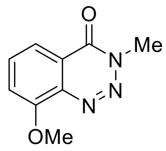
1r

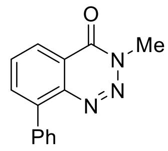
1s

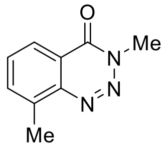
1t

Figure S1. Scope of benzotriazinones

2. ^{1}H and ^{13}C NMR spectra

4-((4-oxobenzo[d][1,2,3]triazin-3(4H)-yl)methyl)benzaldehyde (1g)

^{1}H NMR (400 MHz, $CDCl_3$)

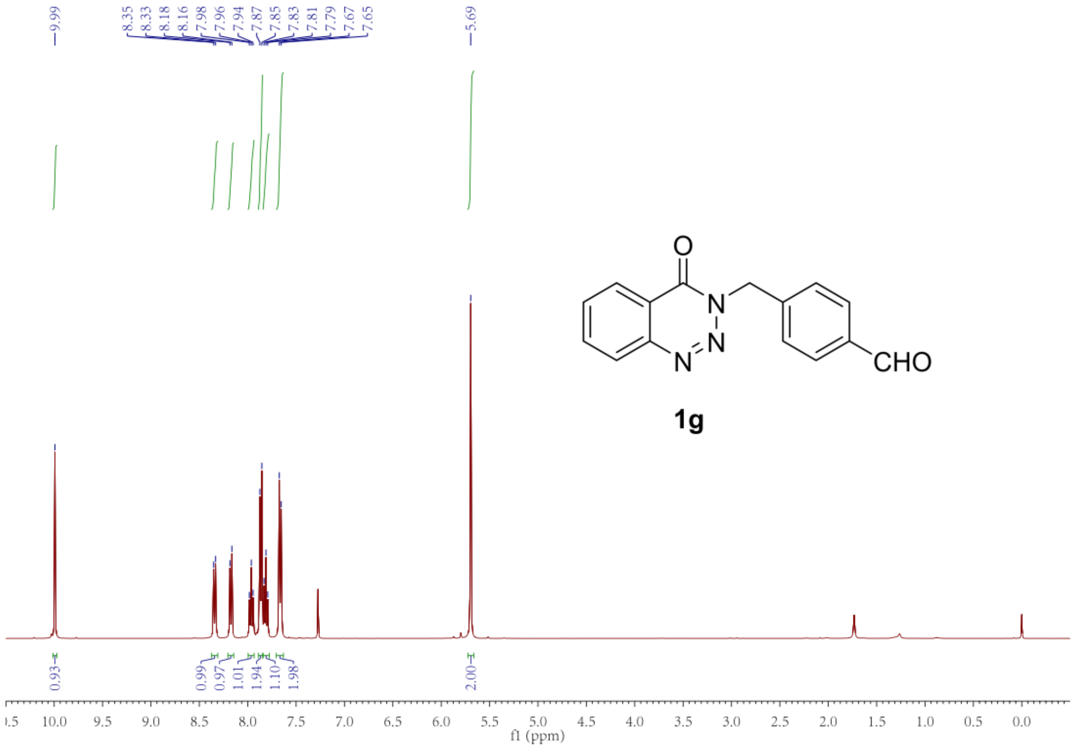

^{13}C NMR (100 MHz, $CDCl_3$)

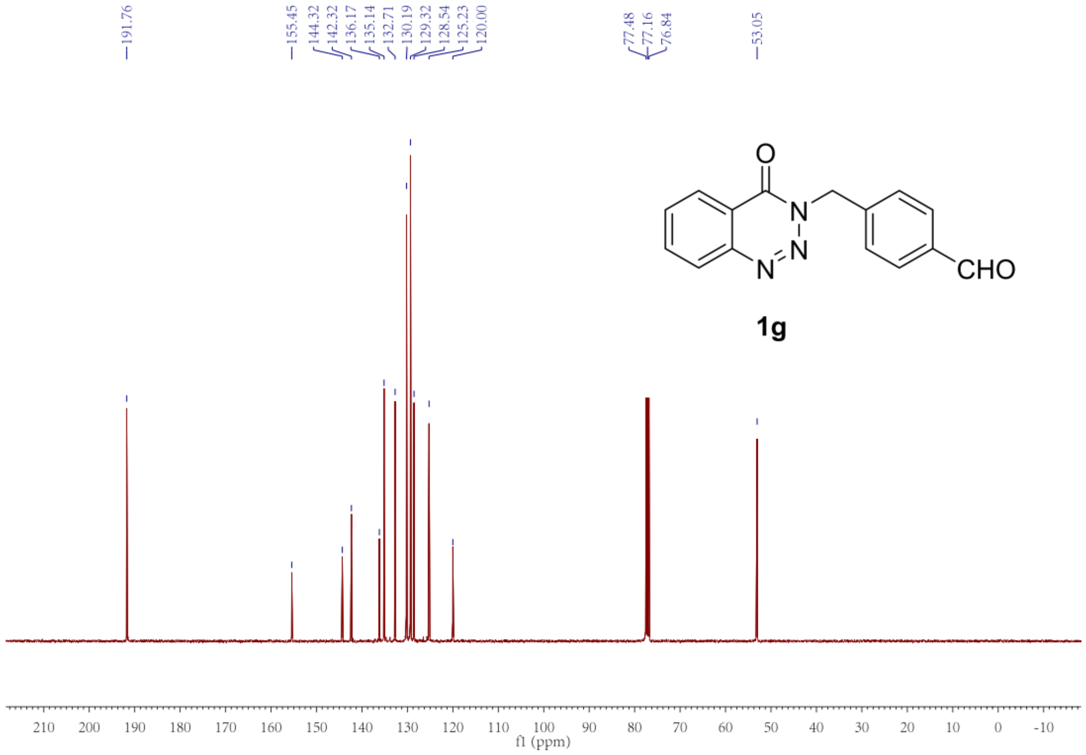

4-((4-oxobenzo[d][1,2,3]triazin-3(4H)-yl)methyl)benzonitrile (1h)

^{1}H NMR (400 MHz, $CDCl_3$)

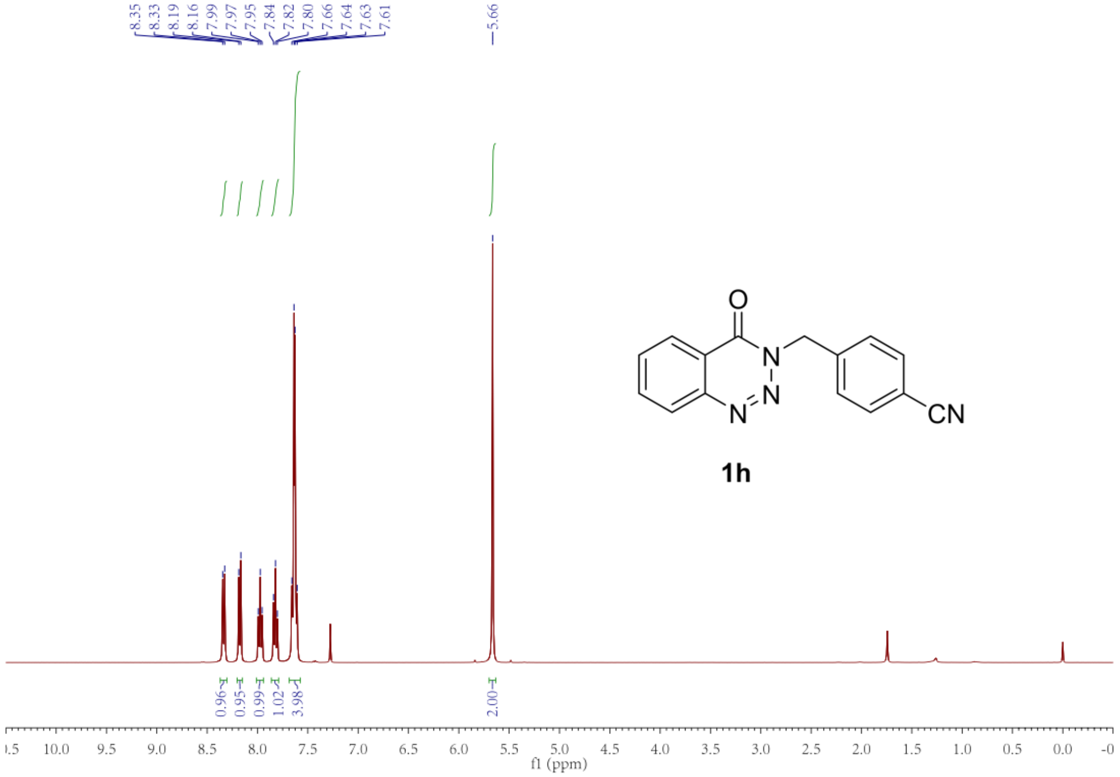

^{13}C NMR (100 MHz, $CDCl_3$)

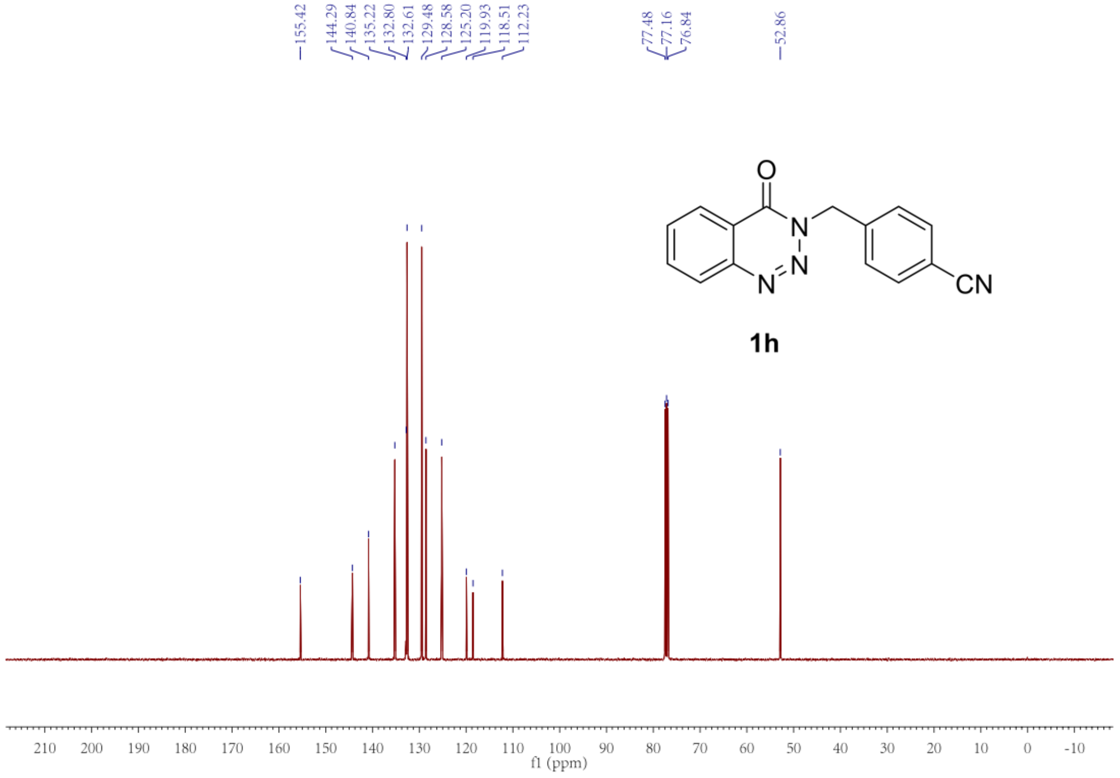

6-bromo-3-methylbenzo[d][1,2,3]triazin-4(3H)-one(1o)

^{1}H NMR (400 MHz, $CDCl_3$)

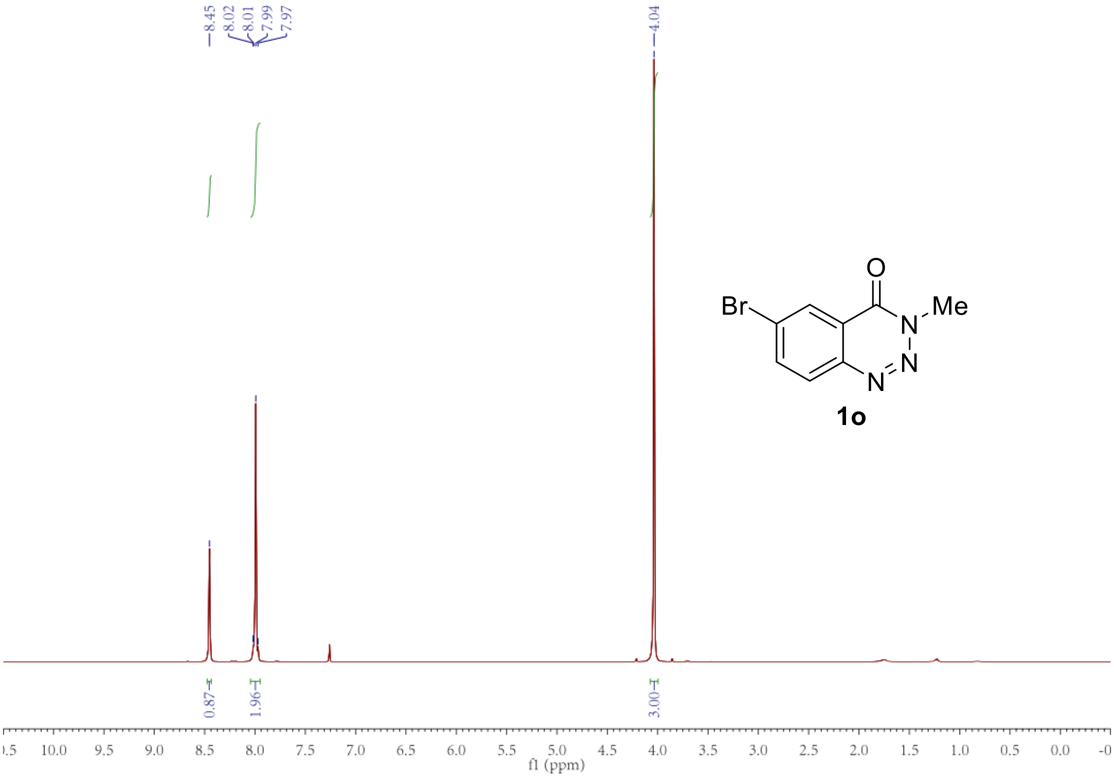

^{13}C NMR (100 MHz, $CDCl_3$)

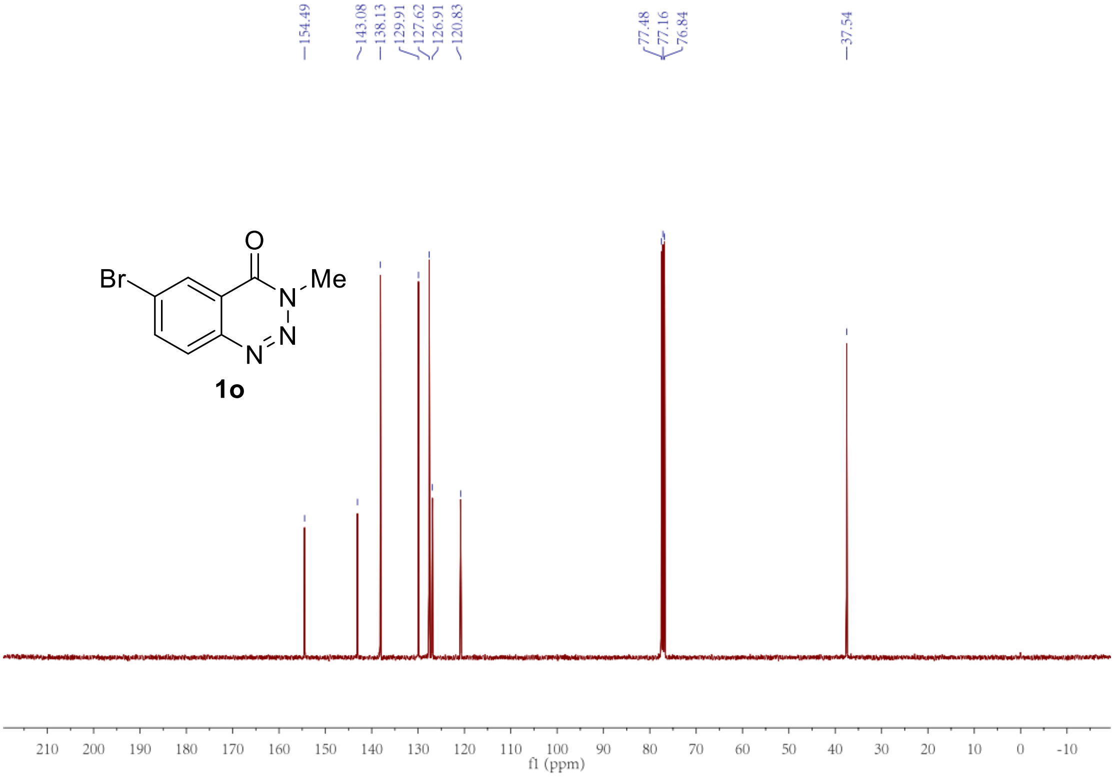

3,6-dimethylbenzo[d][1,2,3]triazin-4(3H)-one(1p)

^{1}H NMR (400 MHz, $CDCl_3$)

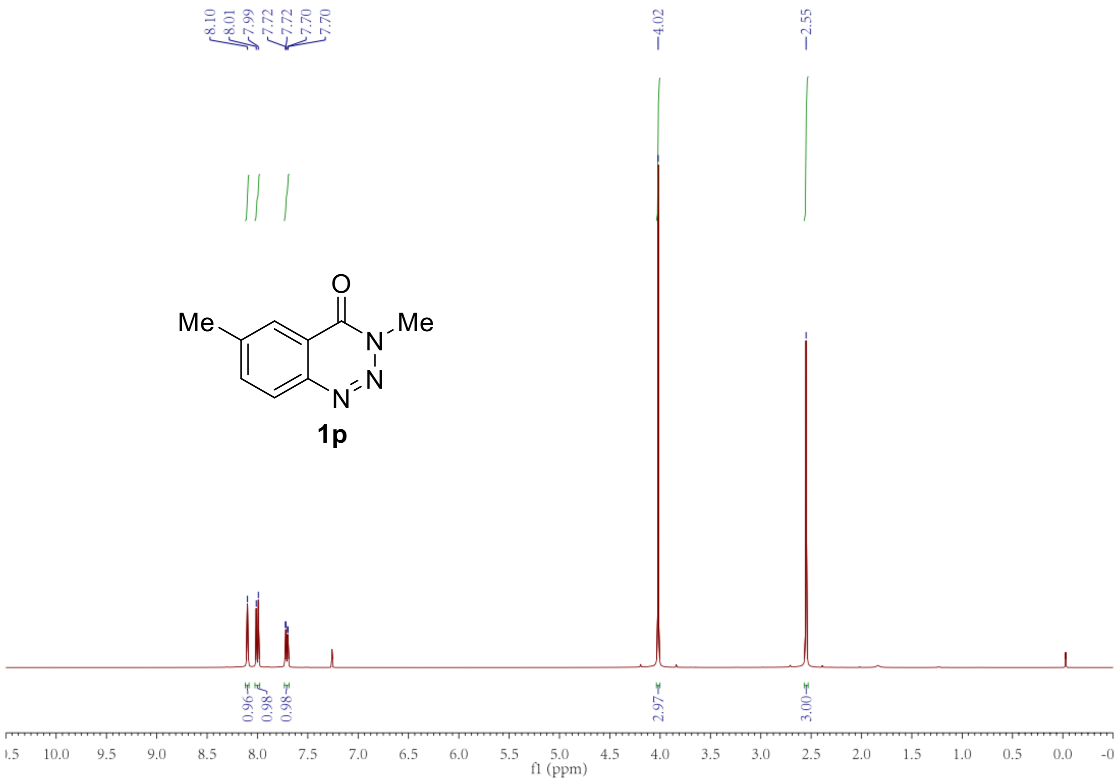

^{13}C NMR (100 MHz, $CDCl_3$)

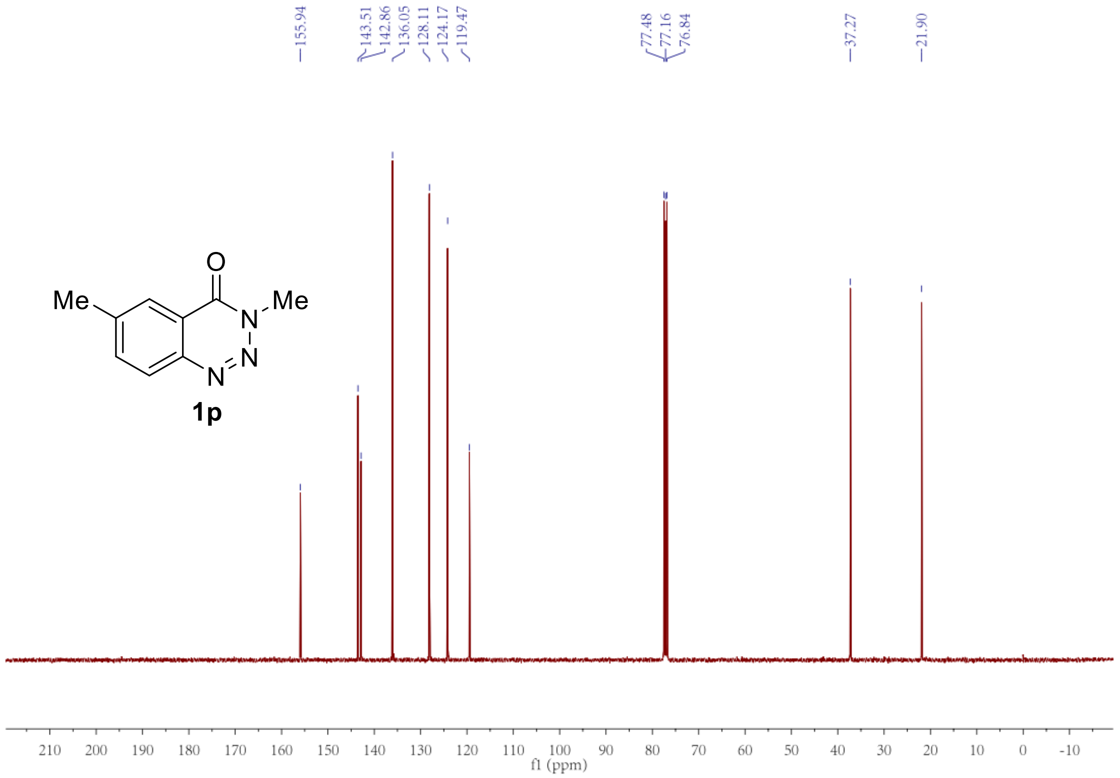

3-methyl-8-phenylbenzo[d][1,2,3]triazin-4(3H)-one (1s)

^{1}H NMR (400 MHz, $CDCl_3$)

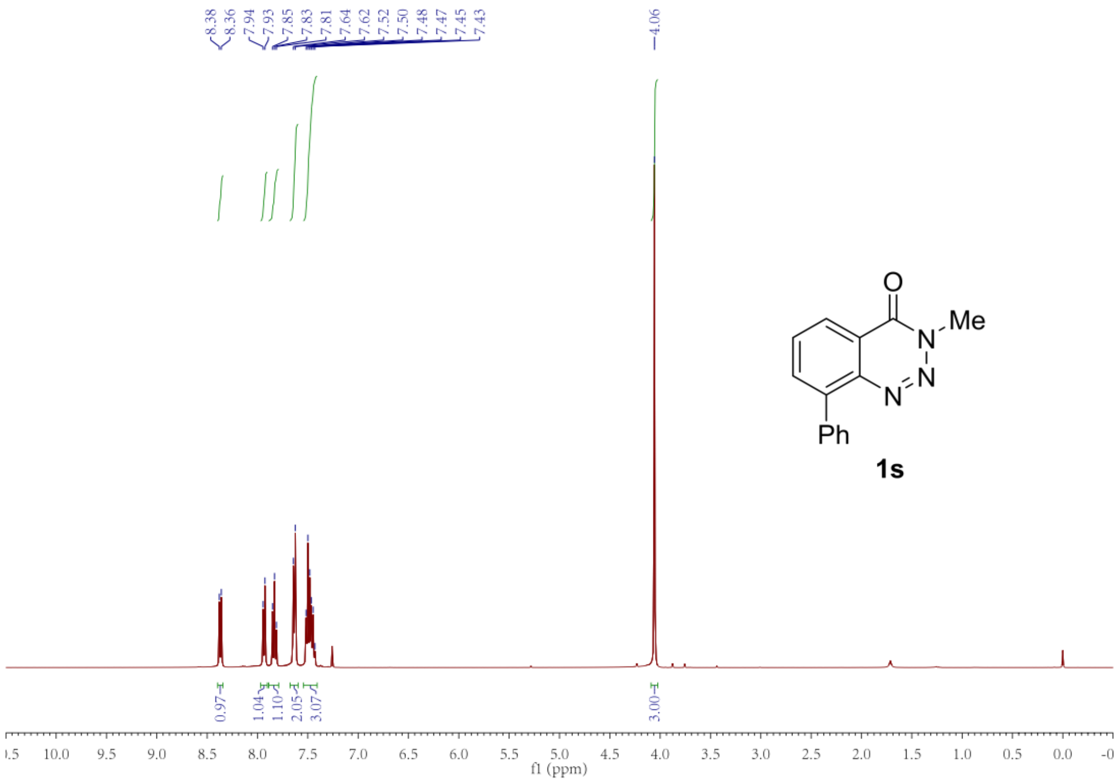

^{13}C NMR (100 MHz, $CDCl_3$)

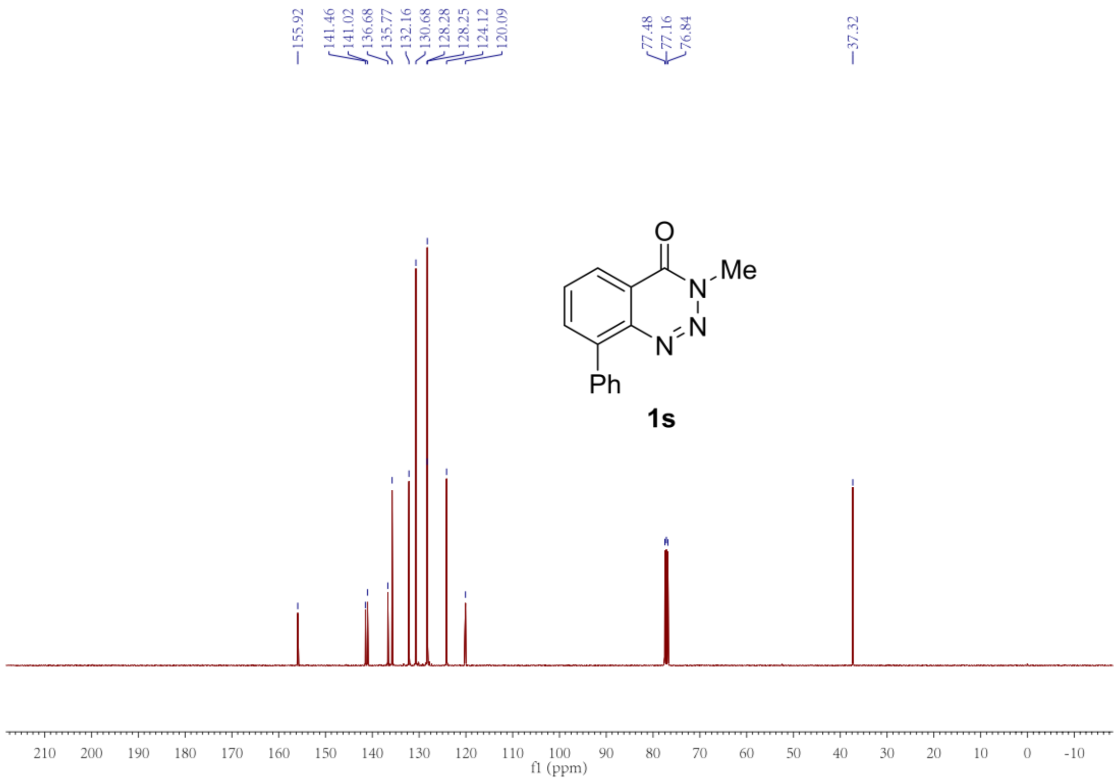

N,2-dimethylbenzamide (3aa)

^{1}H NMR (400 MHz, $CDCl_3$)

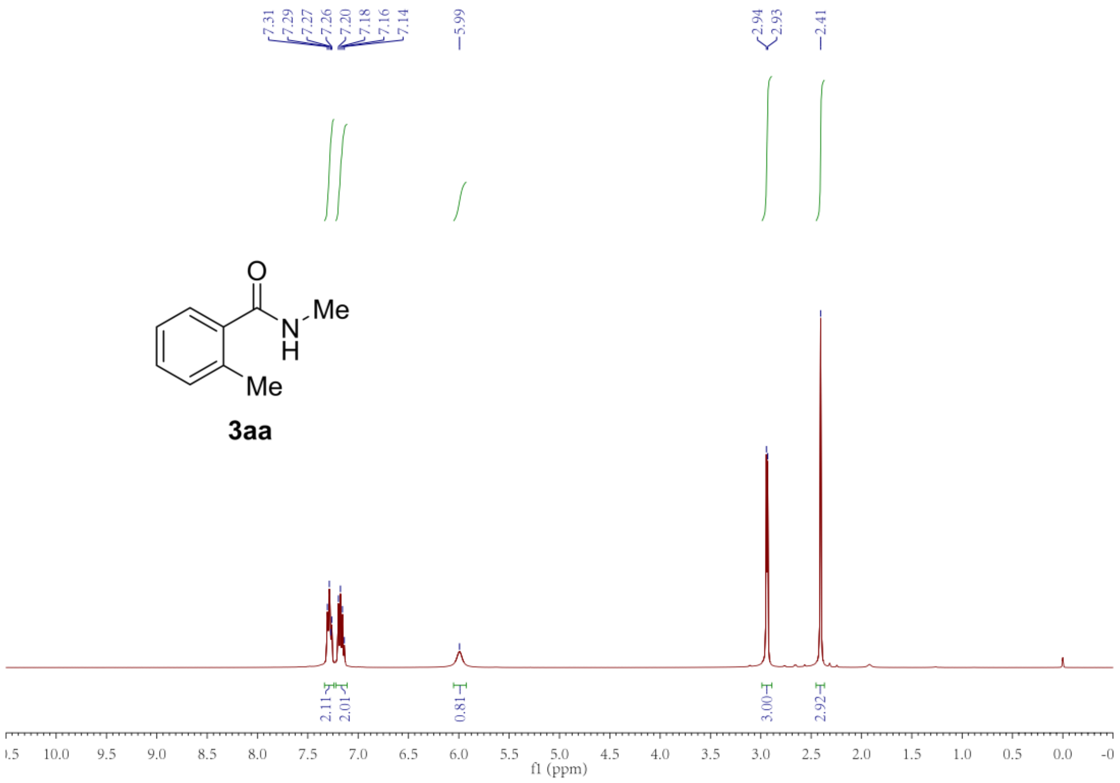

^{13}C NMR (100 MHz, $CDCl_3$)

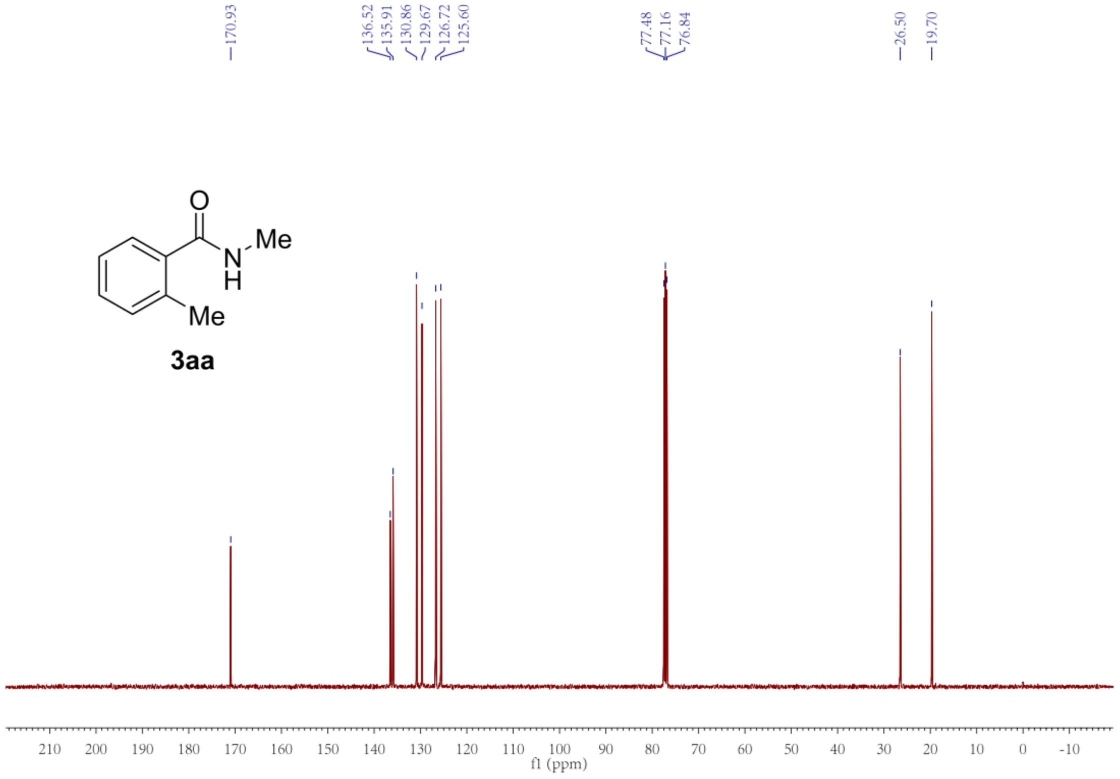

2-ethyl-N-methylbenzamide (3ab)

^{1}H NMR (400 MHz, $CDCl_3$)

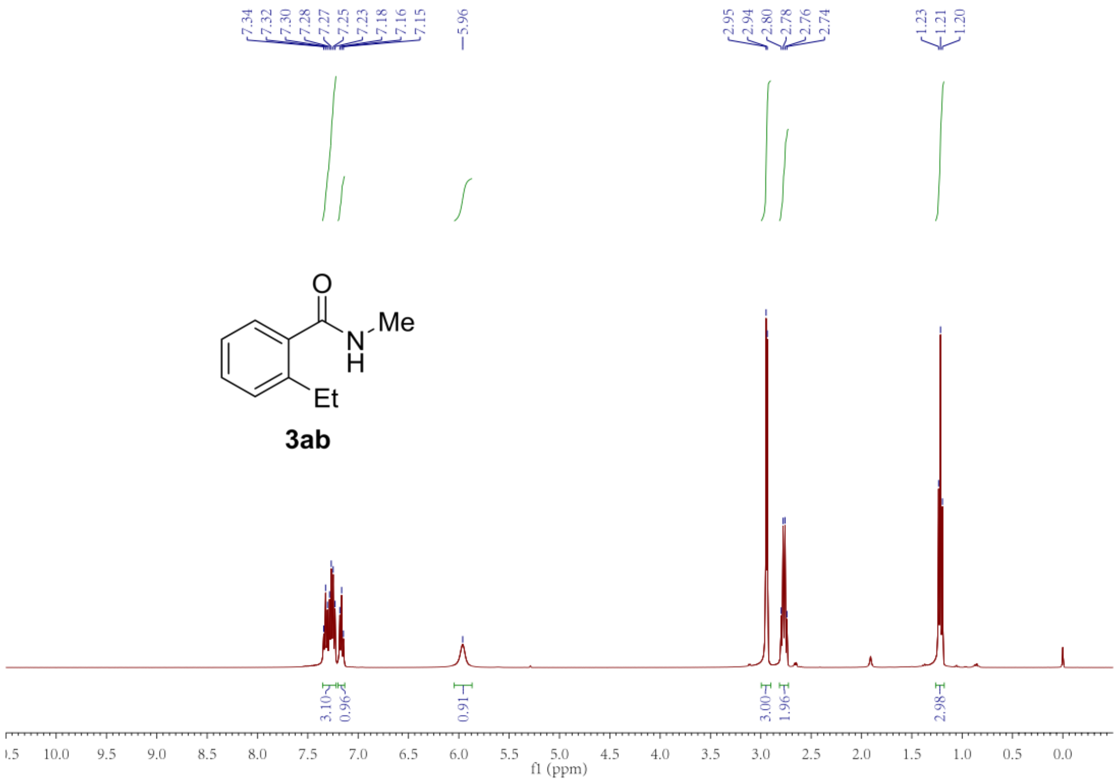

^{13}C NMR (100 MHz, $CDCl_3$)

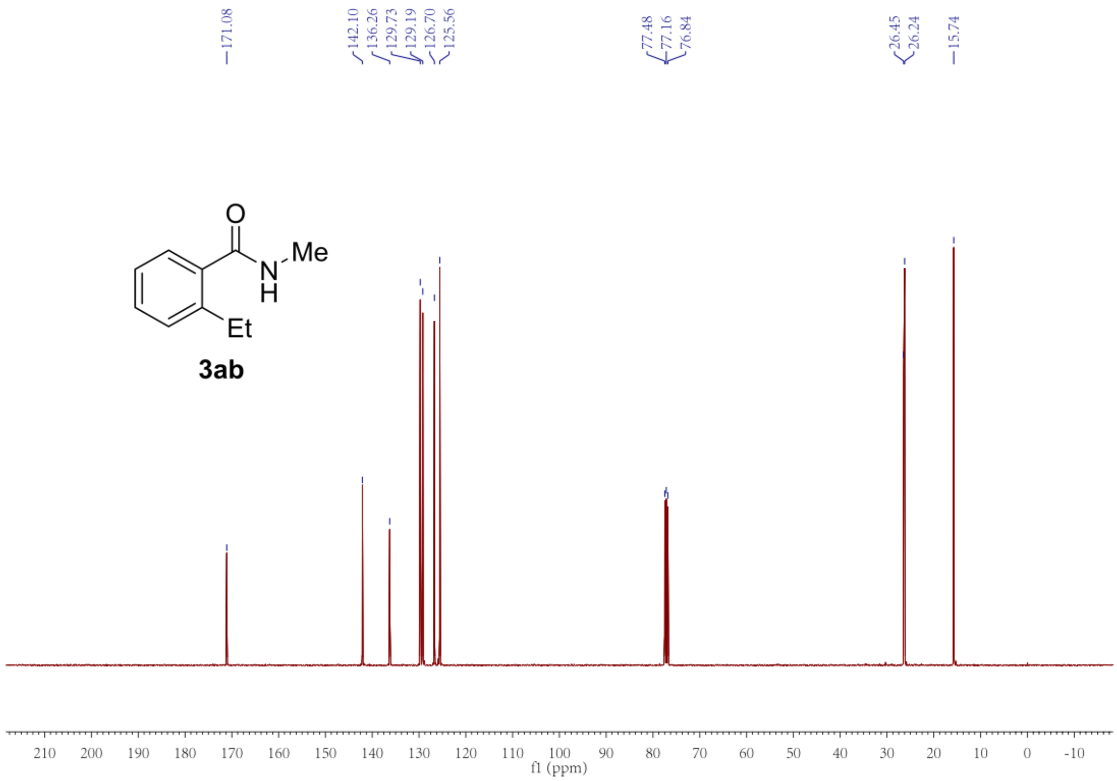

N-methyl-2-(3-phenylpropyl)benzamide (3ac)

^{1}H NMR (400 MHz, $CDCl_3$)

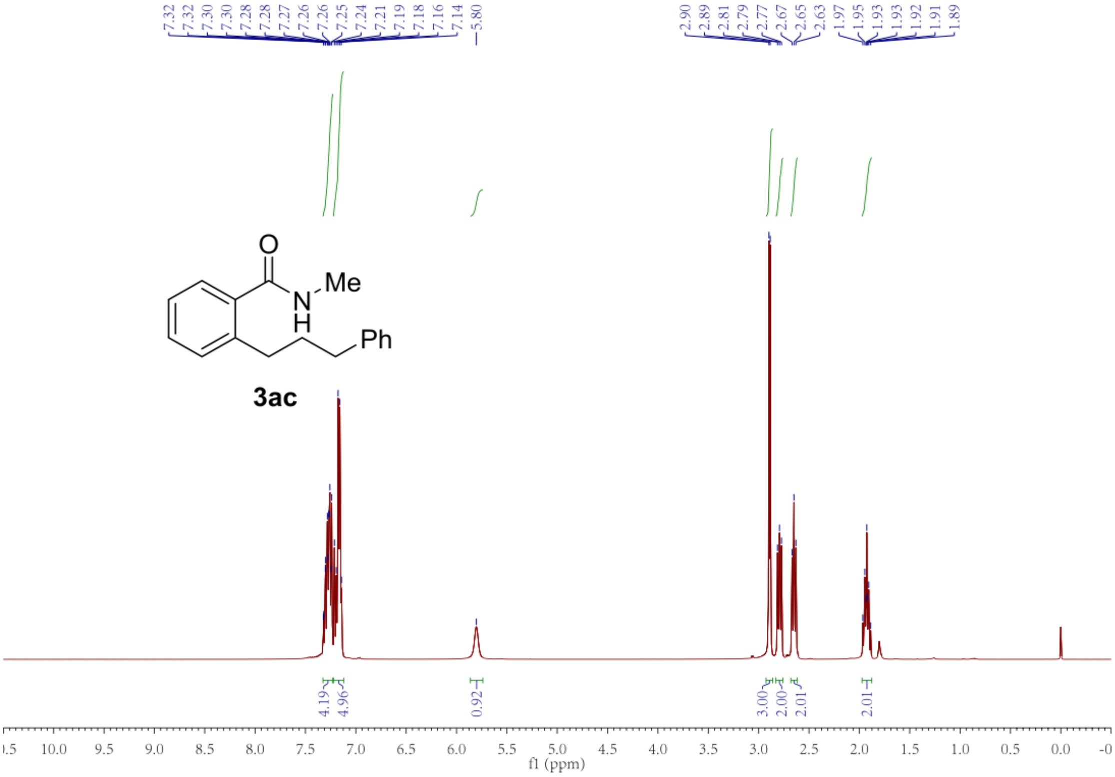

^{13}C NMR (100 MHz, $CDCl_3$)

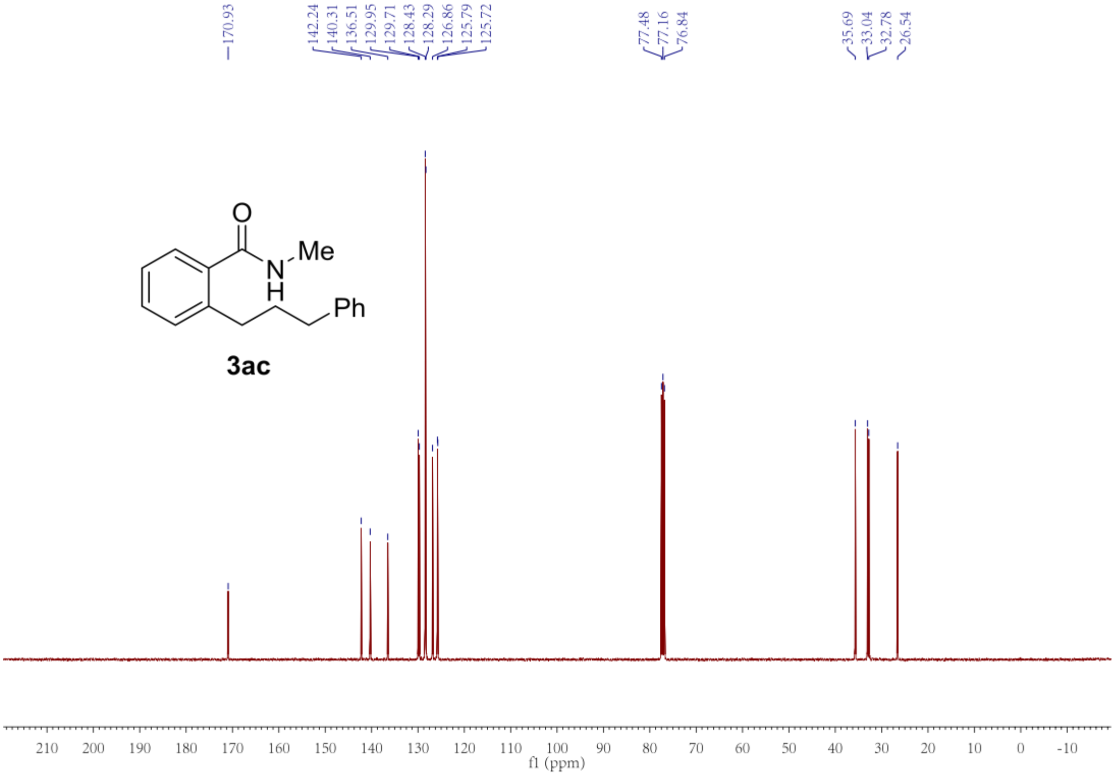

2-(4-chlorobutyl)-N-methylbenzamide (3ad)

^{1}H NMR (400 MHz, $CDCl_3$)

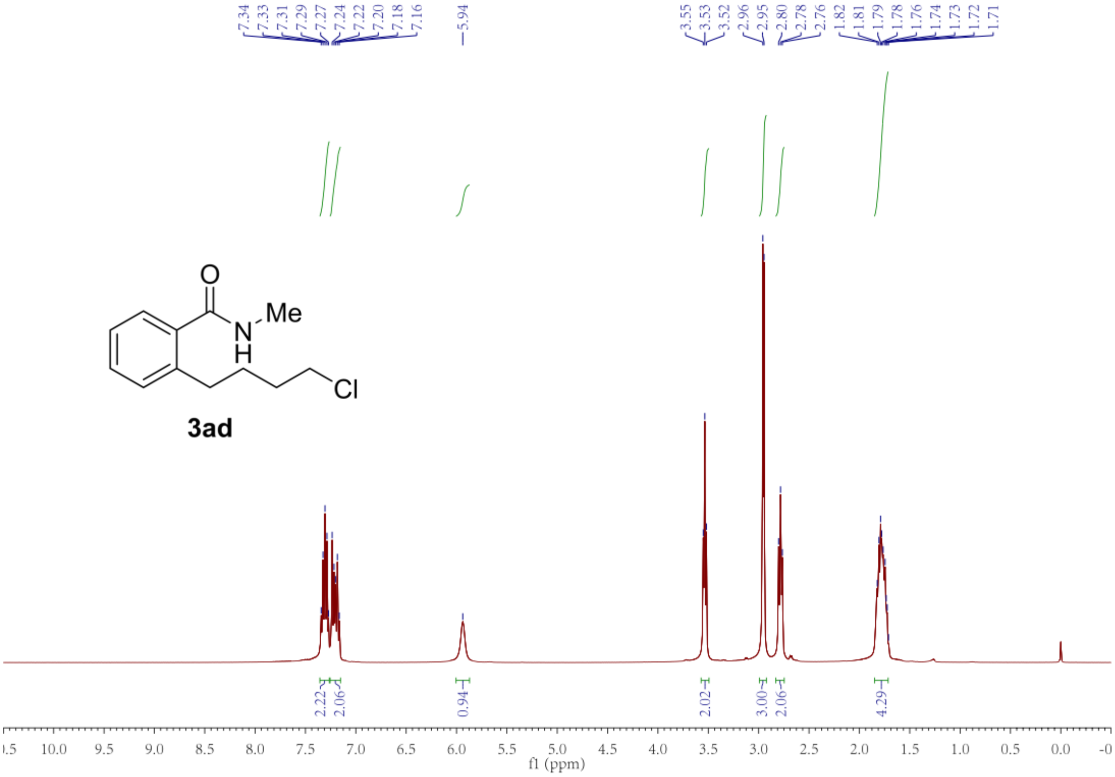

^{13}C NMR (100 MHz, $CDCl_3$)

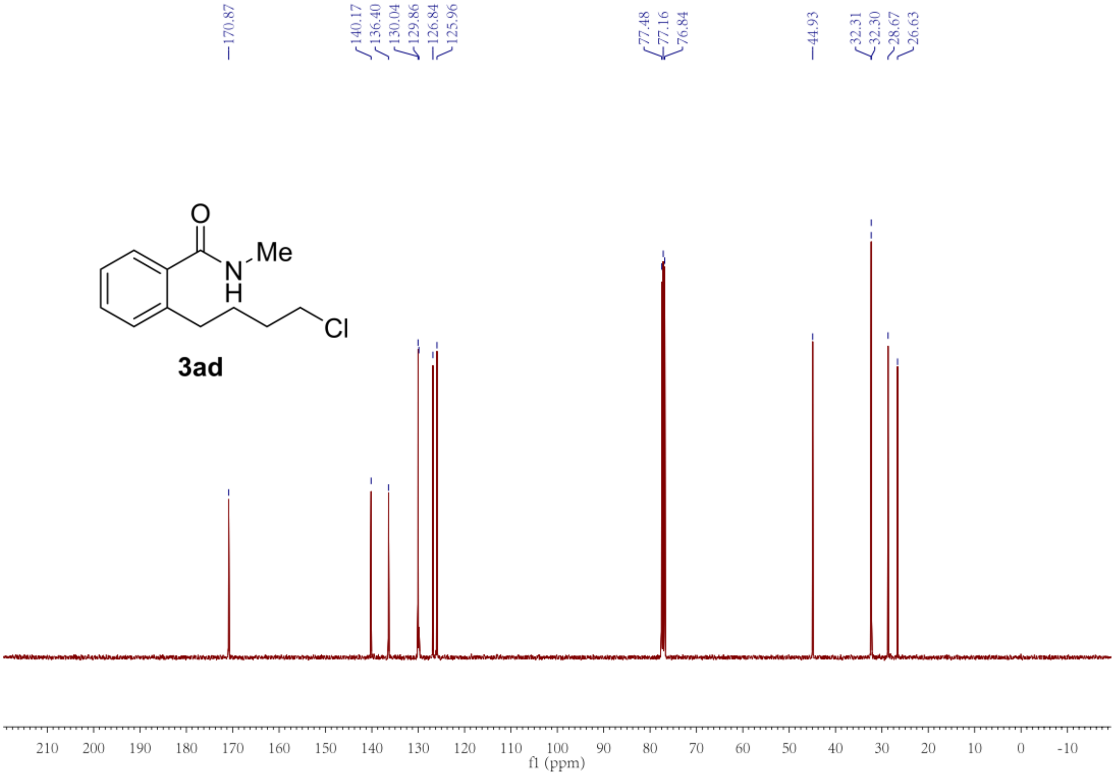

ethyl 6-(2-(methylcarbamoyl)phenyl)hexanoate (3af)

^{1}H NMR (400 MHz, $CDCl_3$)

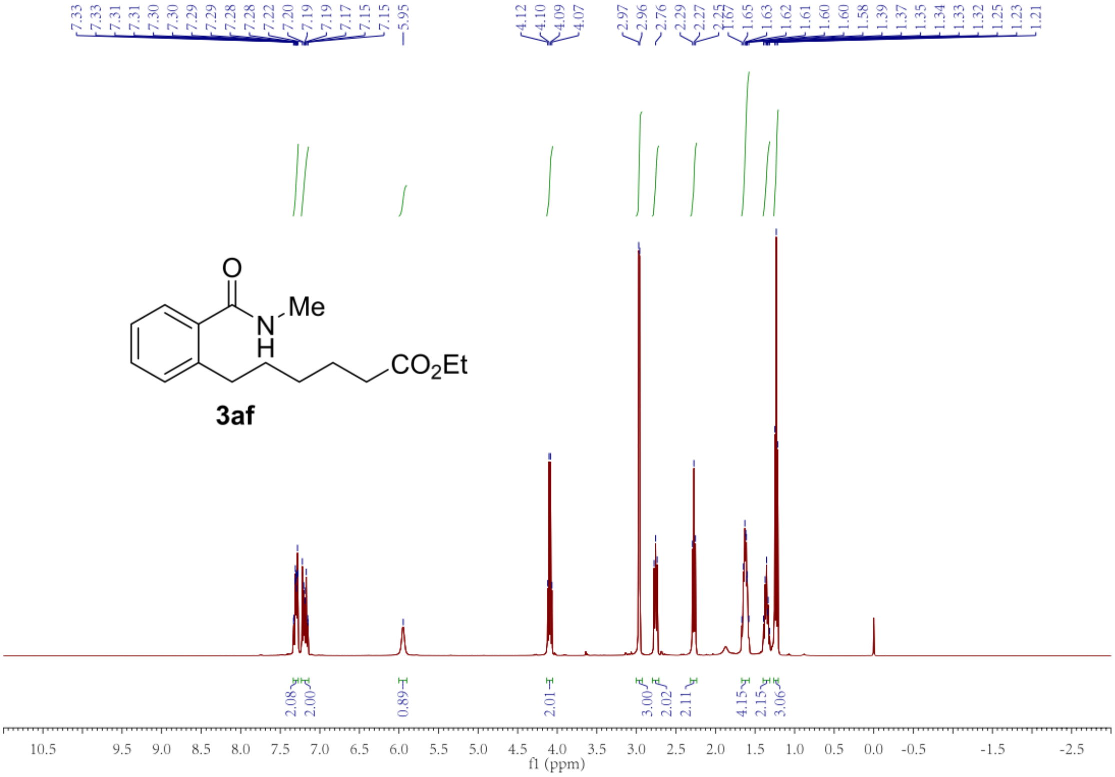

^{13}C NMR (100 MHz, $CDCl_3$)

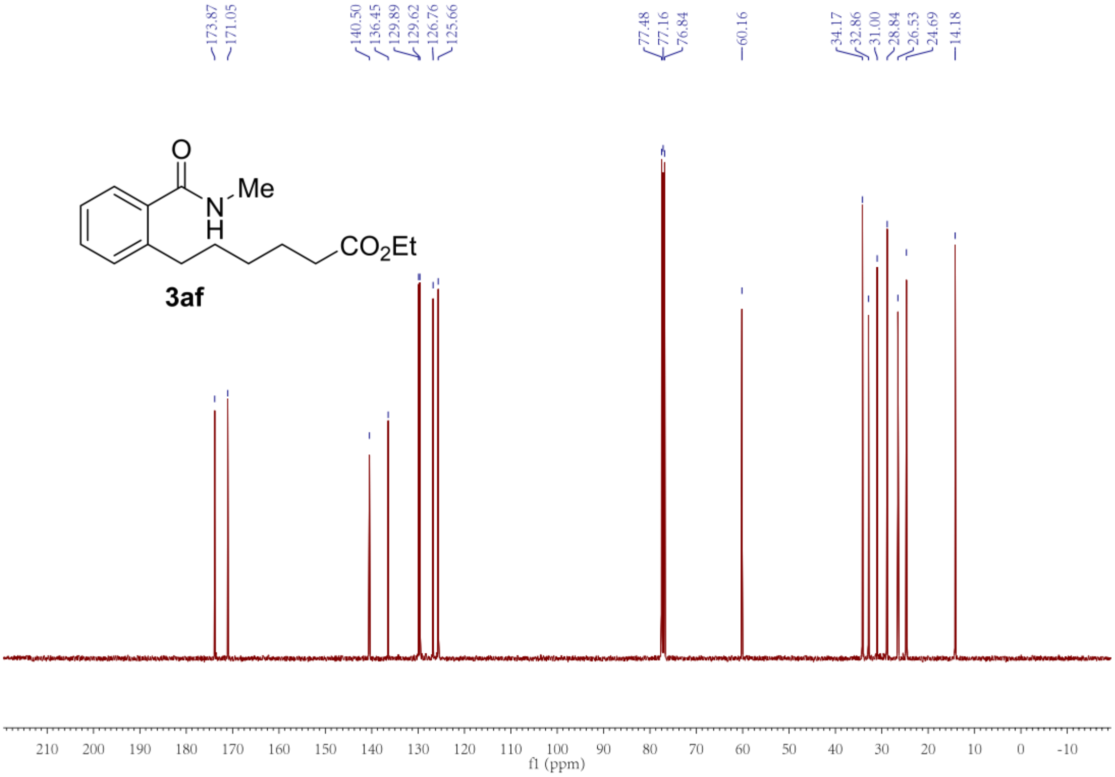

2-benzyl-N-methylbenzamide (3ag)

^{1}H NMR (400 MHz, $CDCl_3$)

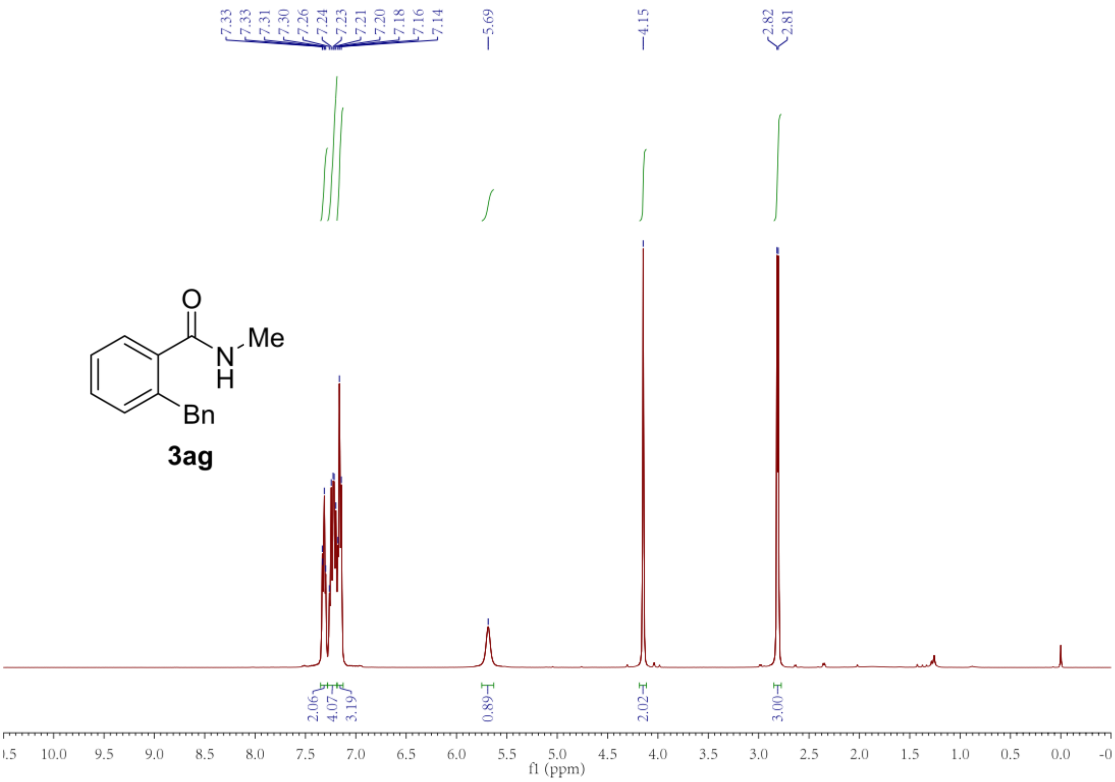

^{13}C NMR (100 MHz, $CDCl_3$)

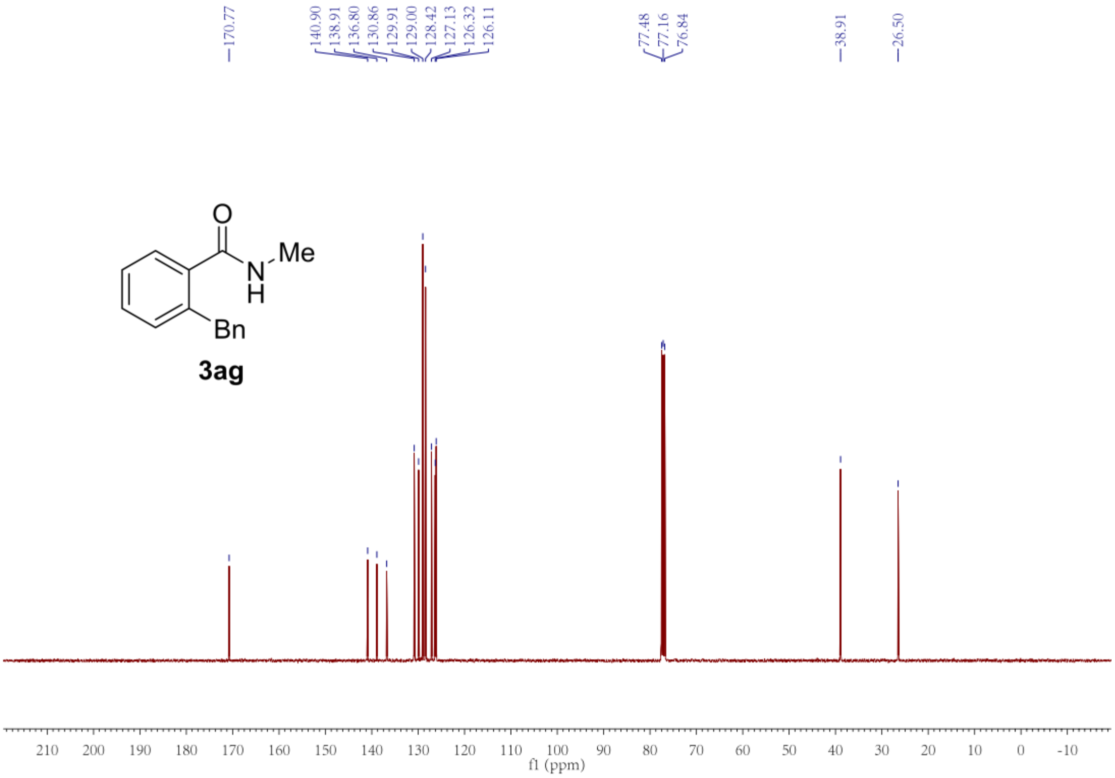

methyl 4-(2-(methylcarbamoyl)benzyl)benzoate (3ah)

^{1}H NMR (400 MHz, $CDCl_3$)

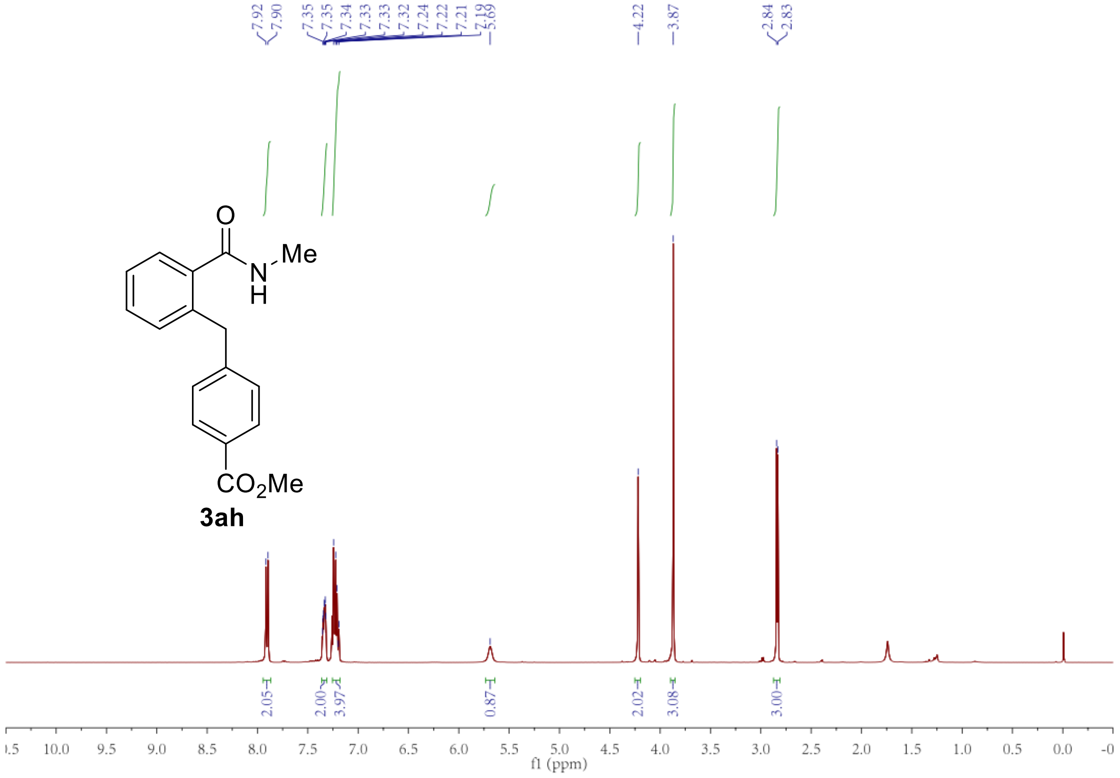

^{13}C NMR (100 MHz, $CDCl_3$)

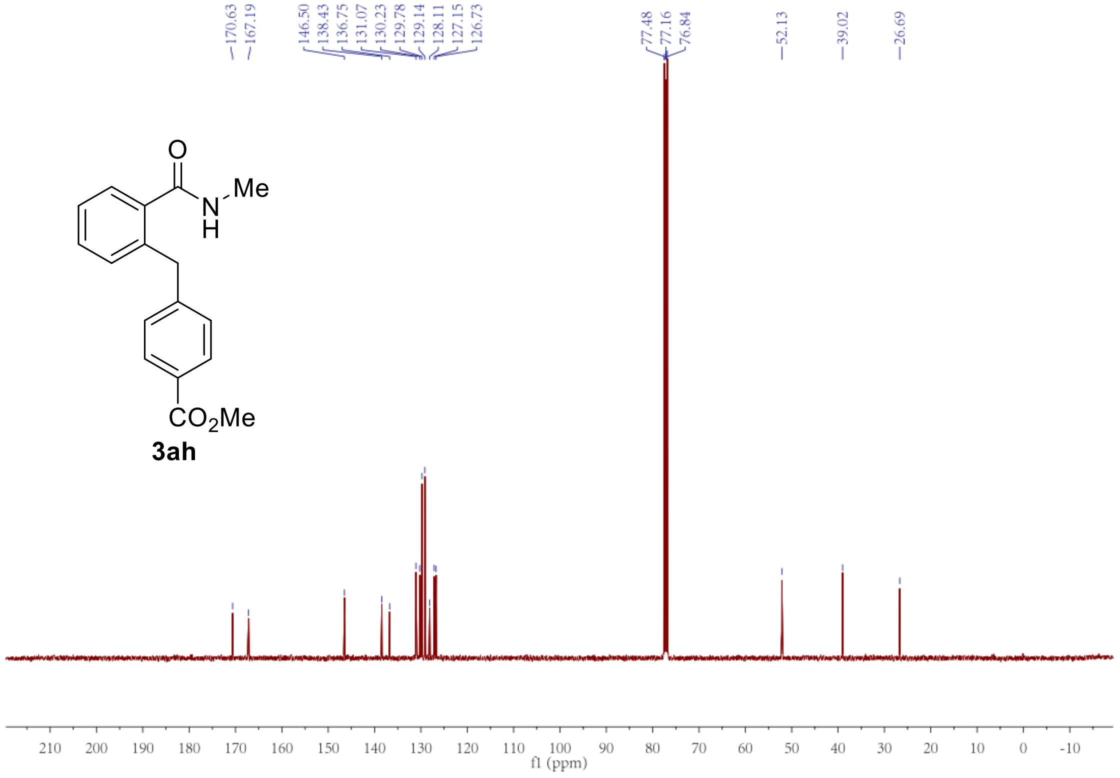

2-(2-ethylhexyl)-N-methylbenzamide (3aj)

^{1}H NMR (400 MHz, $CDCl_3$)

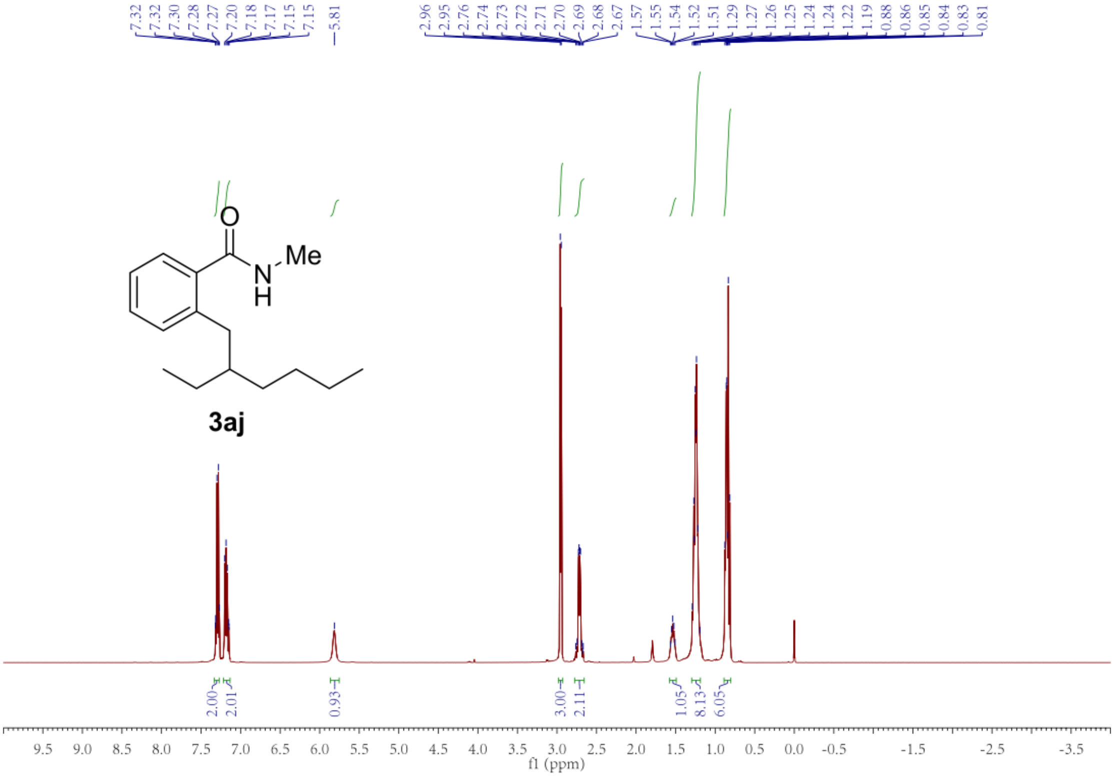

^{13}C NMR (100 MHz, $CDCl_3$)

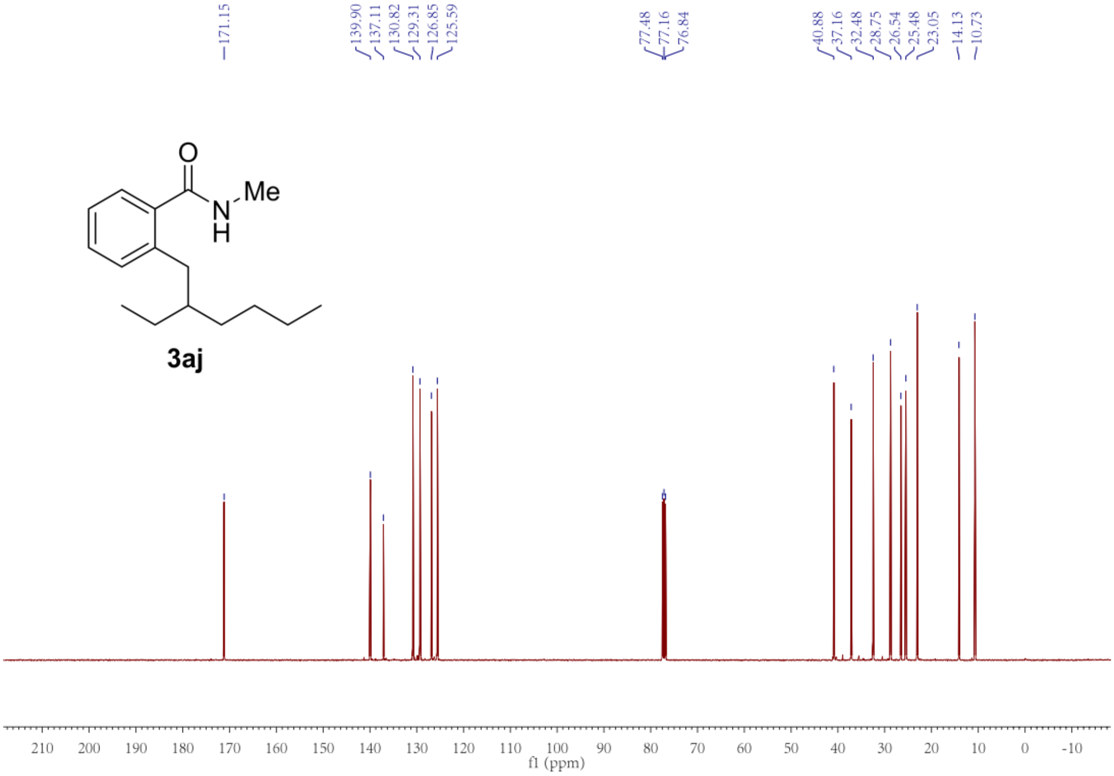

2-(sec-butyl)-N-methylbenzamide (3ak)

^{1}H NMR (400 MHz, $CDCl_3$)

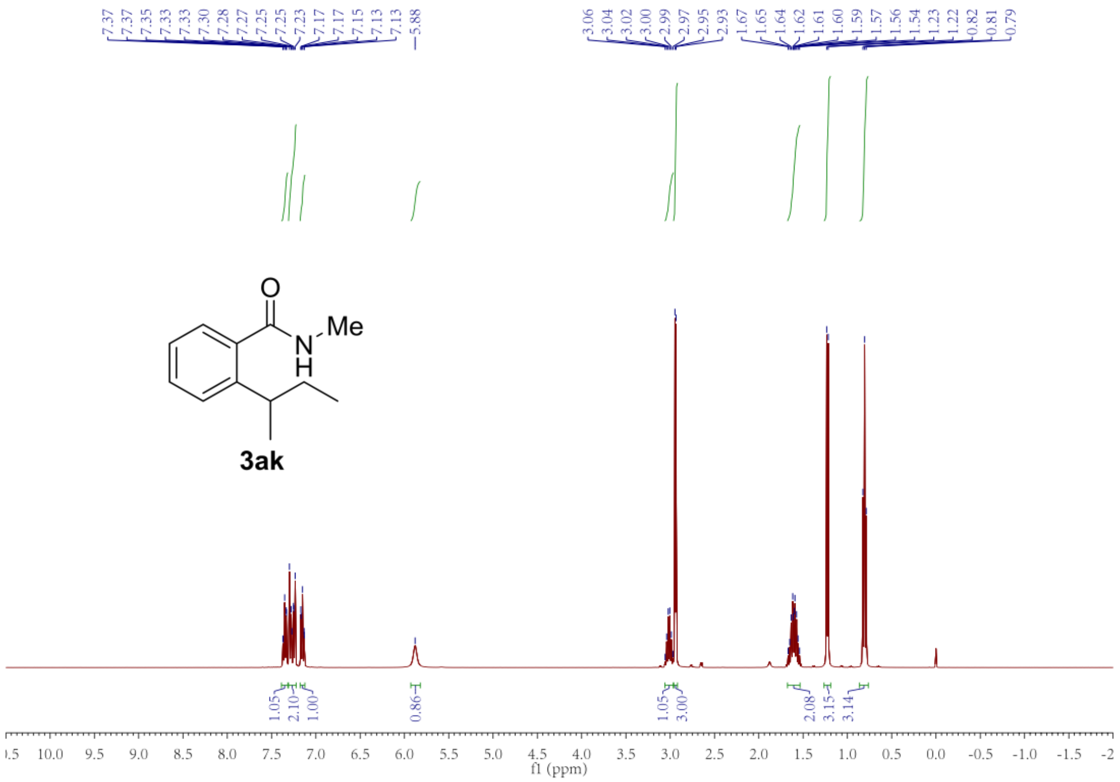

^{13}C NMR (100 MHz, $CDCl_3$)

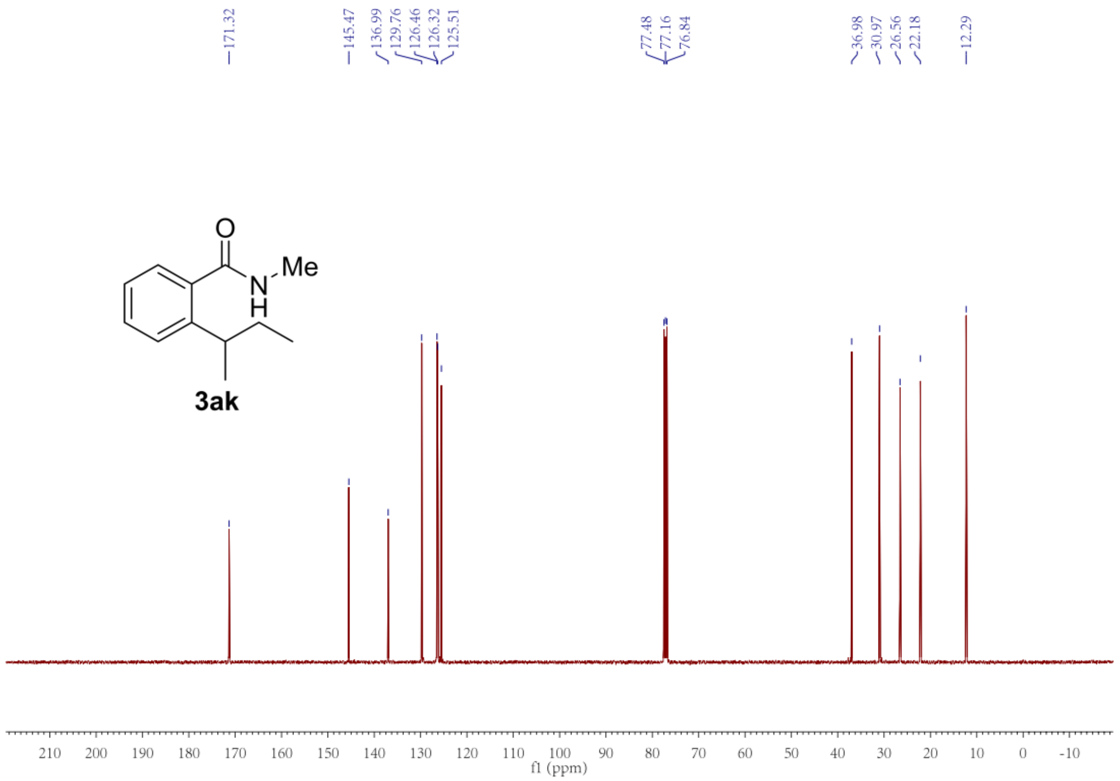

2-isopropyl-N-methylbenzamide (3al)

^{1}H NMR (400 MHz, $CDCl_3$)

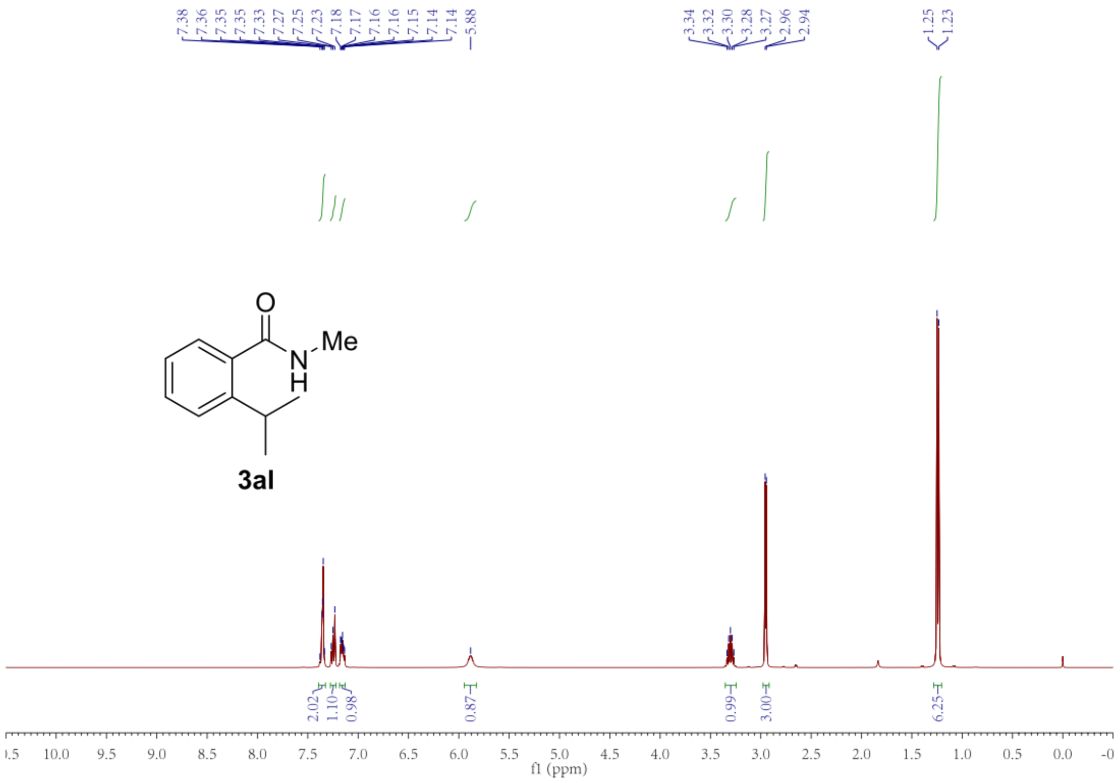

^{13}C NMR (100 MHz, $CDCl_3$)

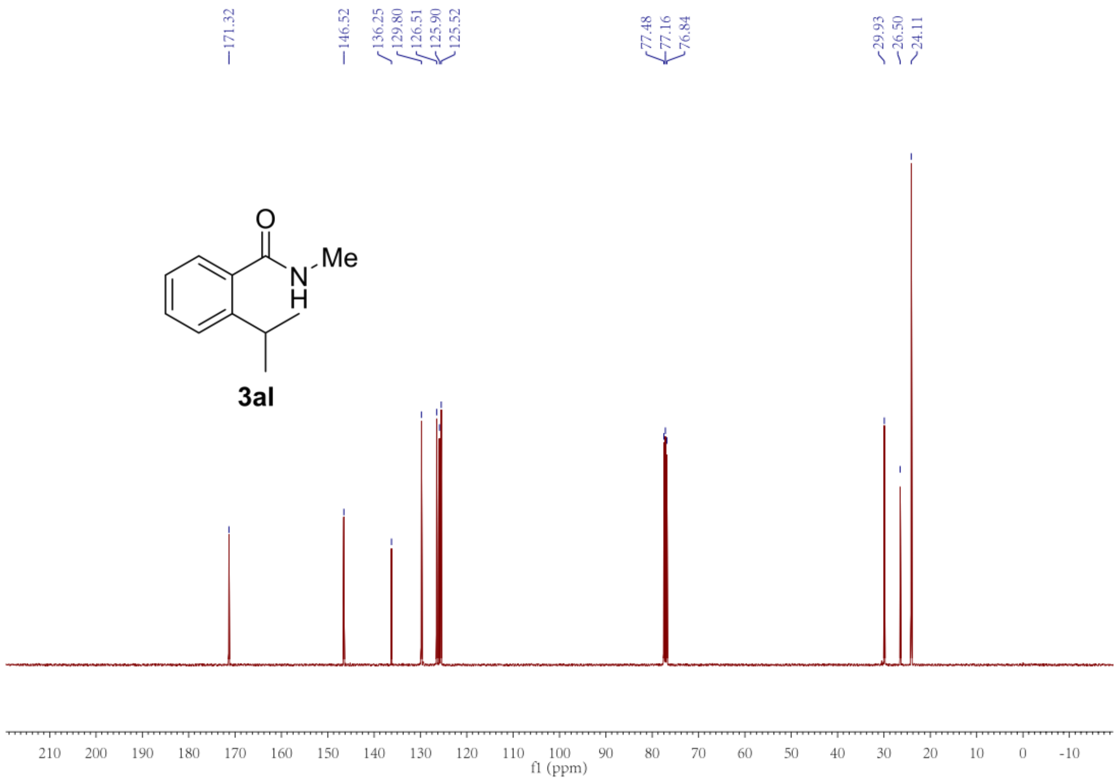

2-ethyl-N-propylbenzamide (3bb)

^{1}H NMR (400 MHz, $CDCl_3$)

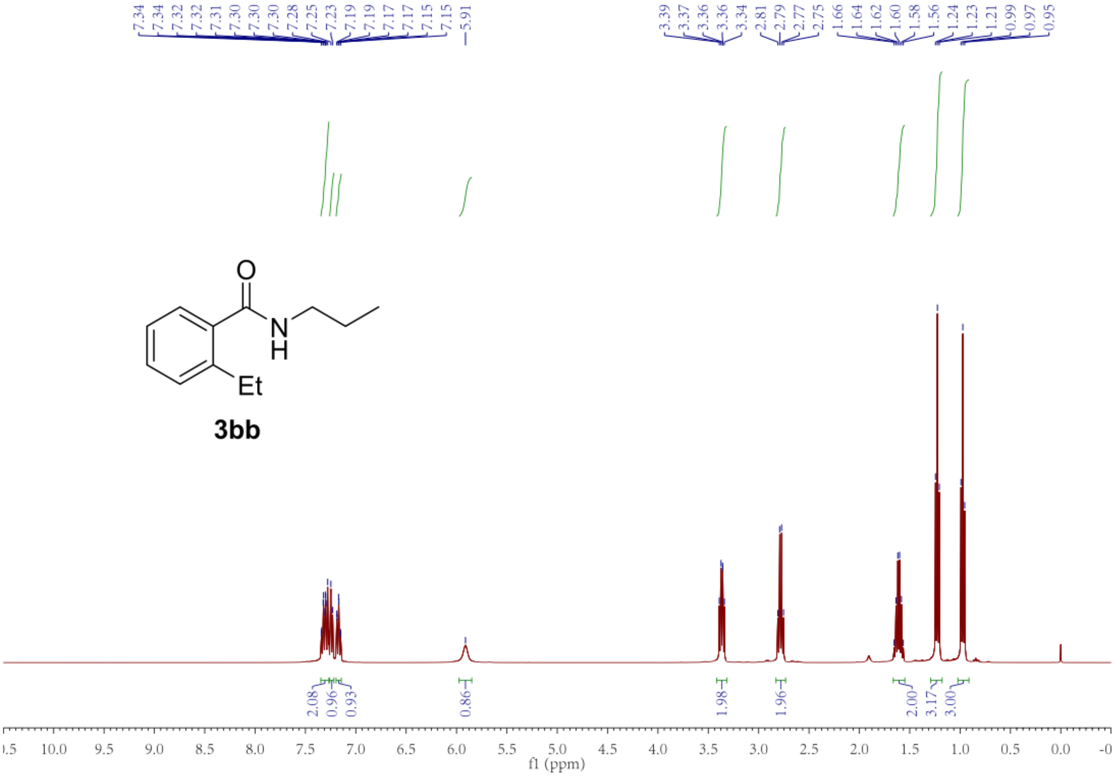

^{13}C NMR (100 MHz, $CDCl_3$)

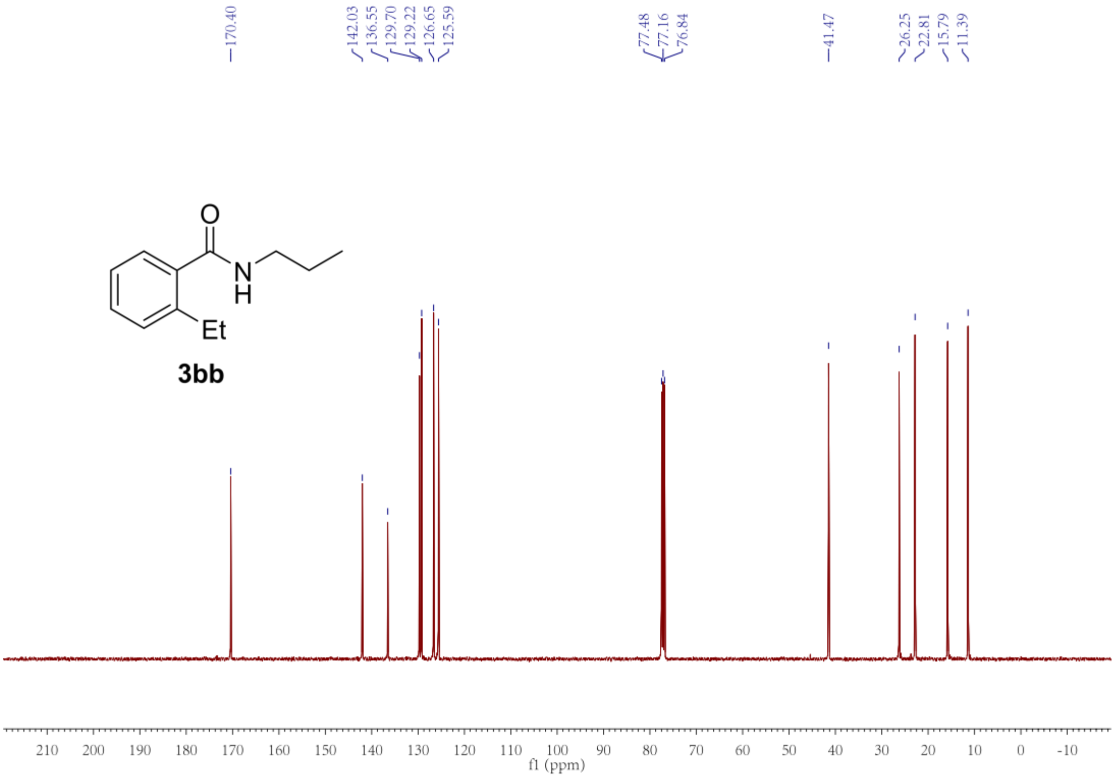

2-ethyl-N-isopropylbenzamide (3cb)

^{1}H NMR (400 MHz, $CDCl_3$)

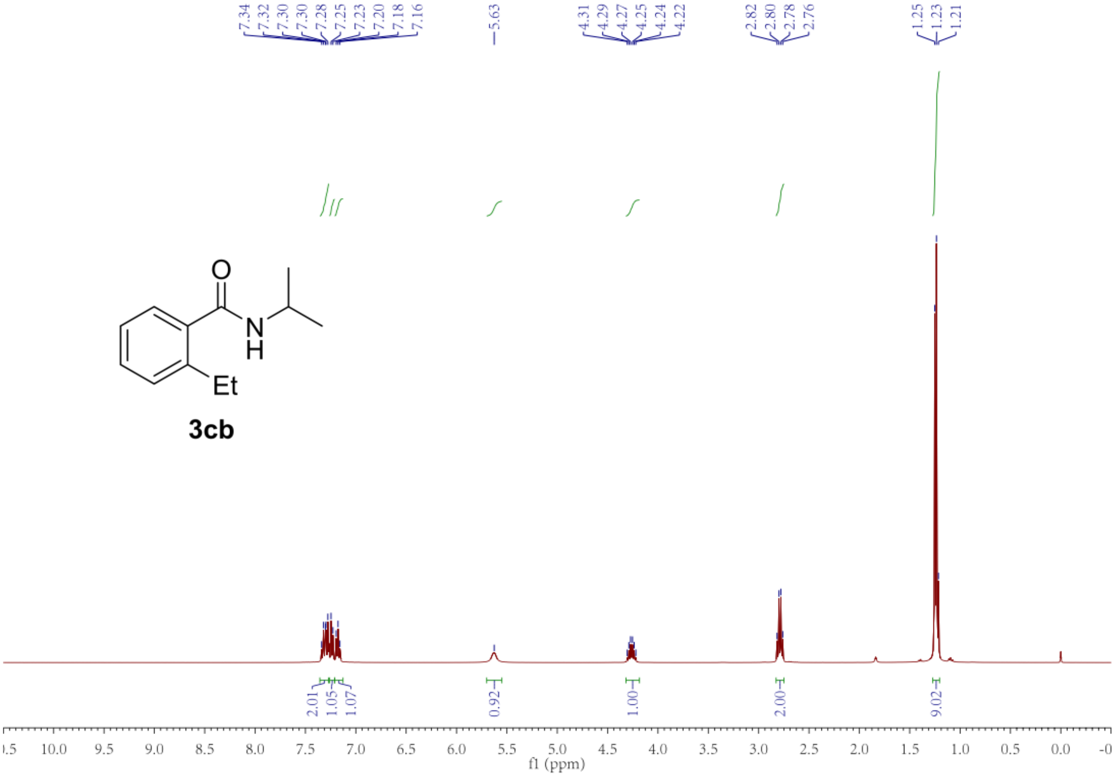

^{13}C NMR (100 MHz, $CDCl_3$)

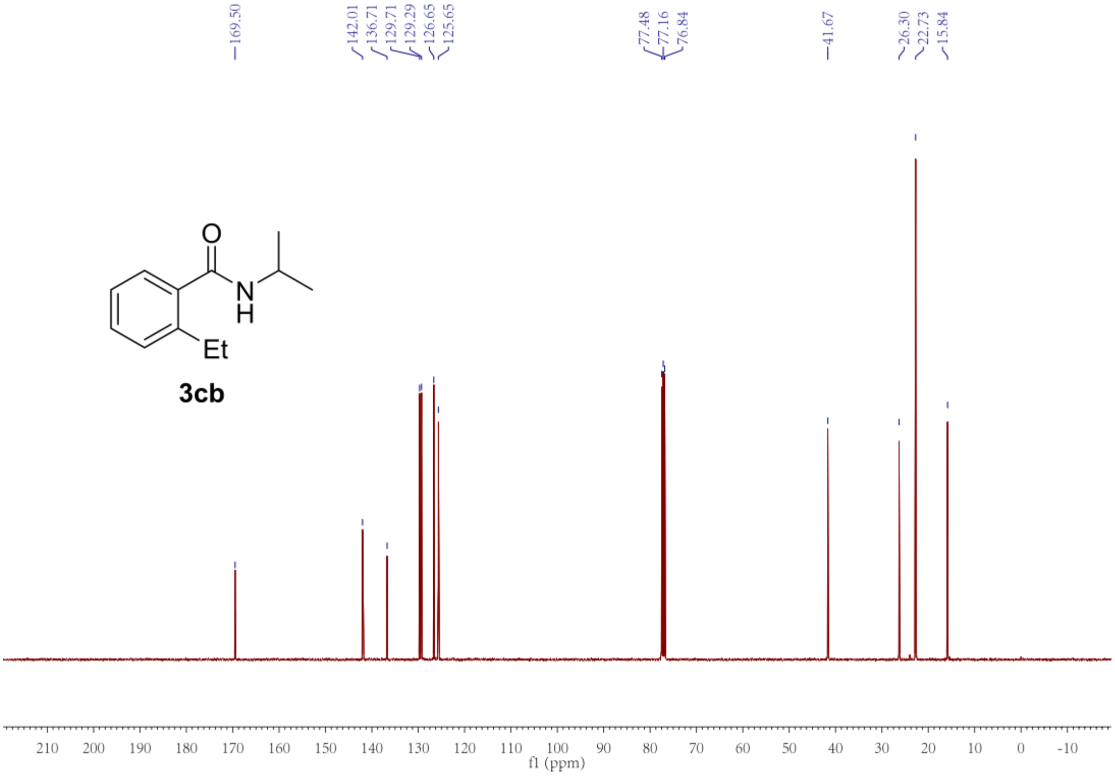

N-cyclohexyl-2-ethylbenzamide (3db)

^{1}H NMR (400 MHz, $CDCl_3$)

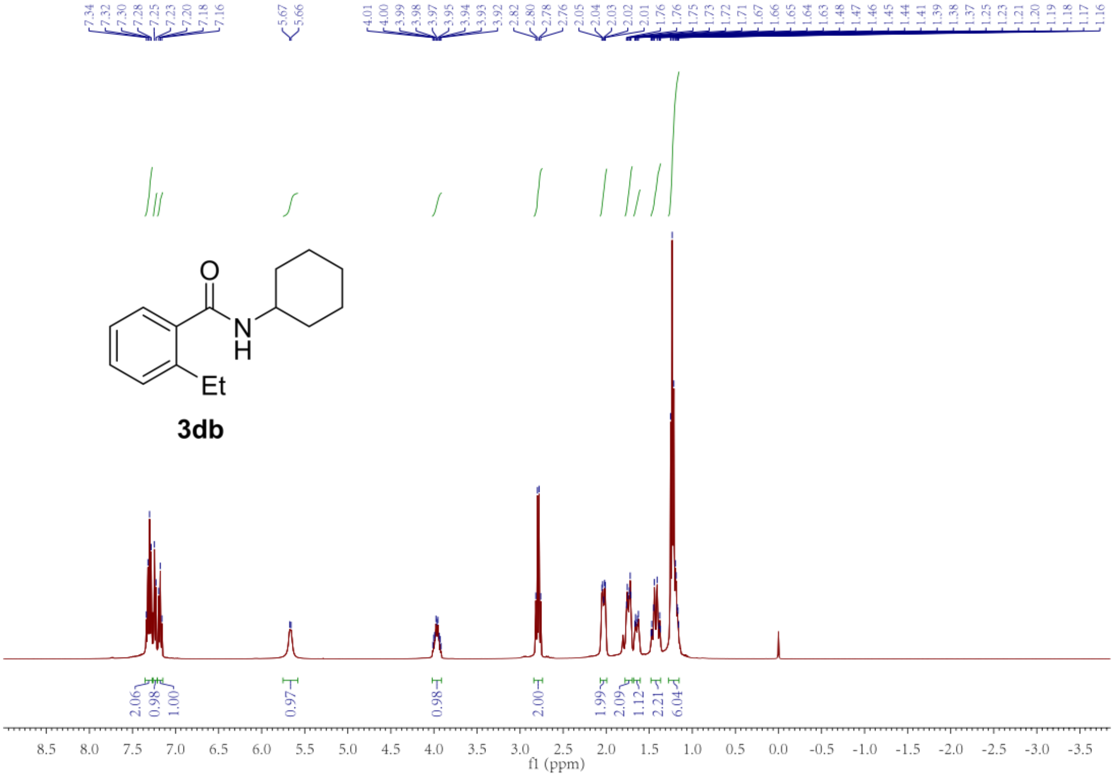

^{13}C NMR (100 MHz, $CDCl_3$)

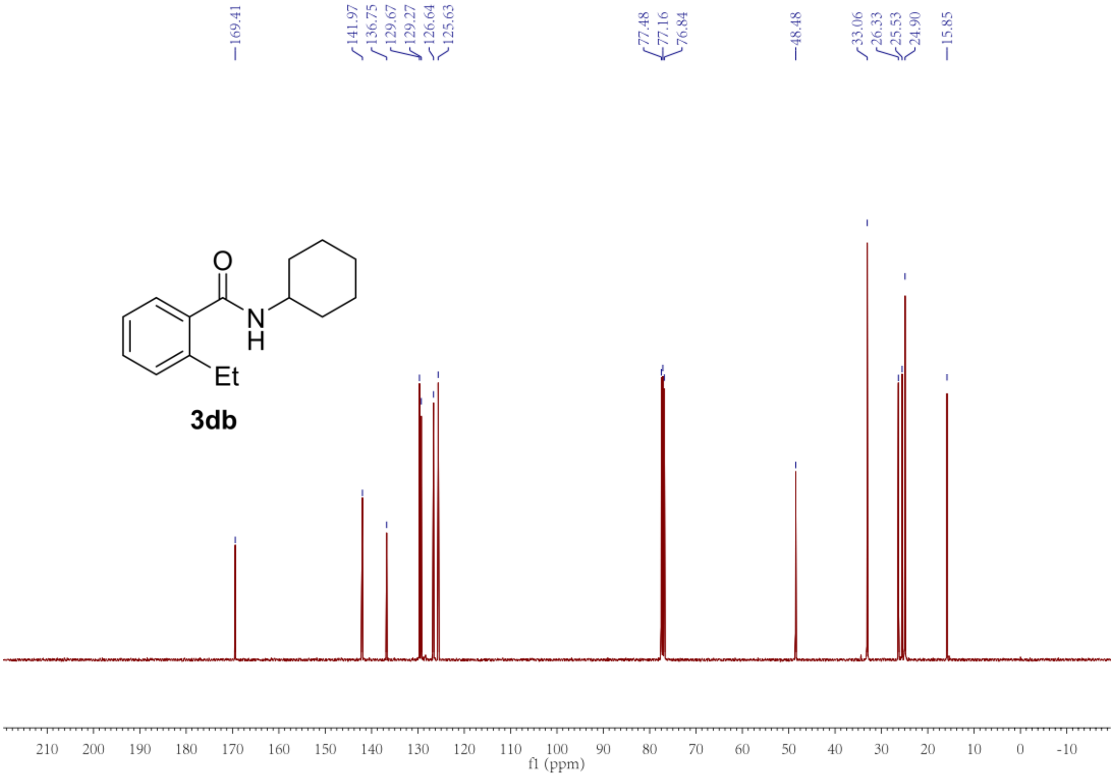

2-ethyl-N-(2-methoxyethyl)benzamide (3eb)

^{1}H NMR (400 MHz, $CDCl_3$)

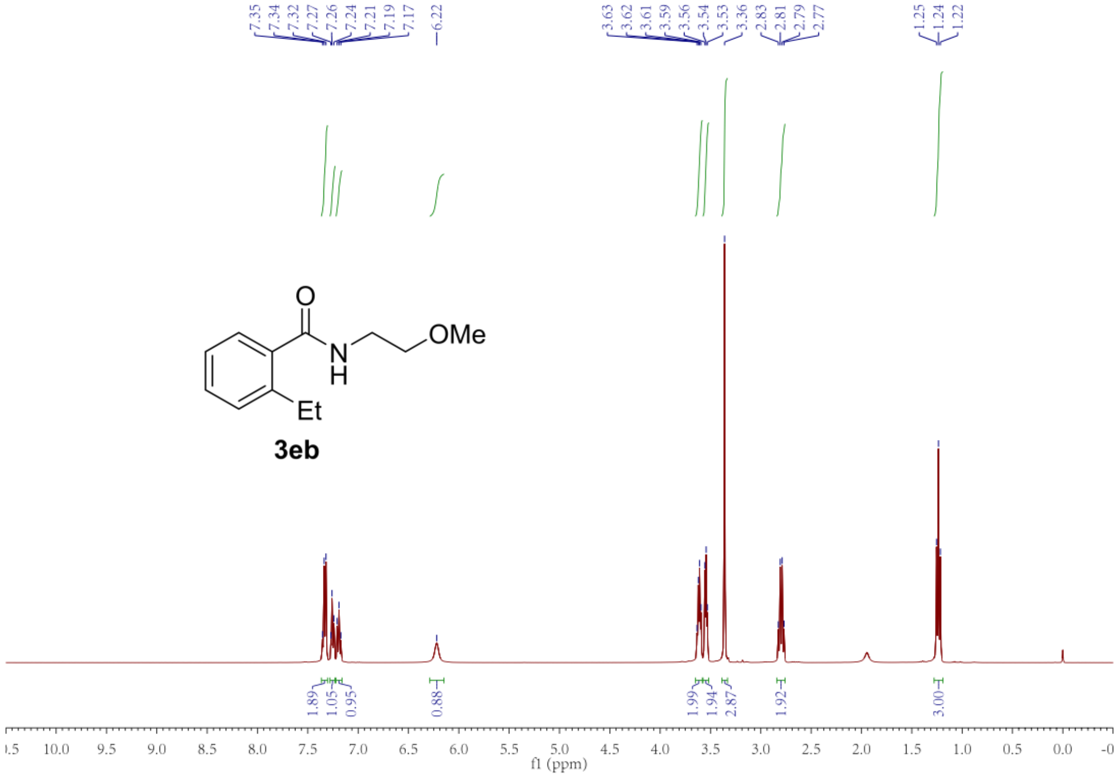

^{13}C NMR (100 MHz, $CDCl_3$)

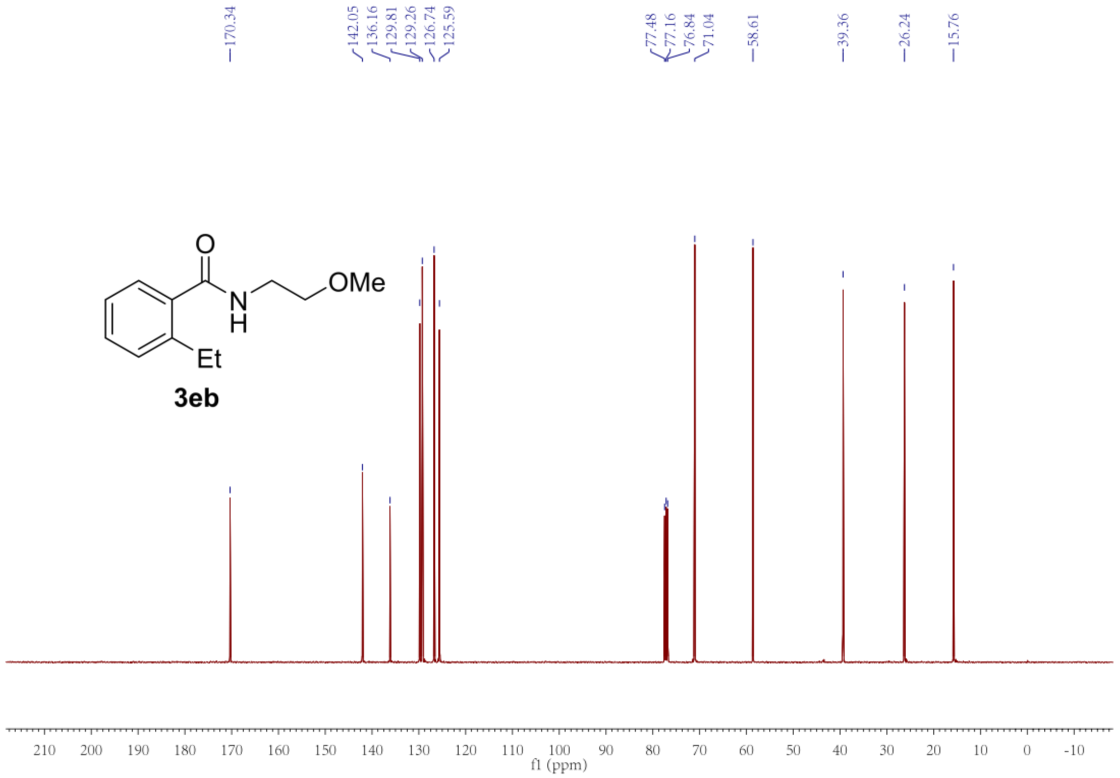

N-benzyl-2-ethylbenzamide (3fb)

^{1}H NMR (400 MHz, $CDCl_3$)

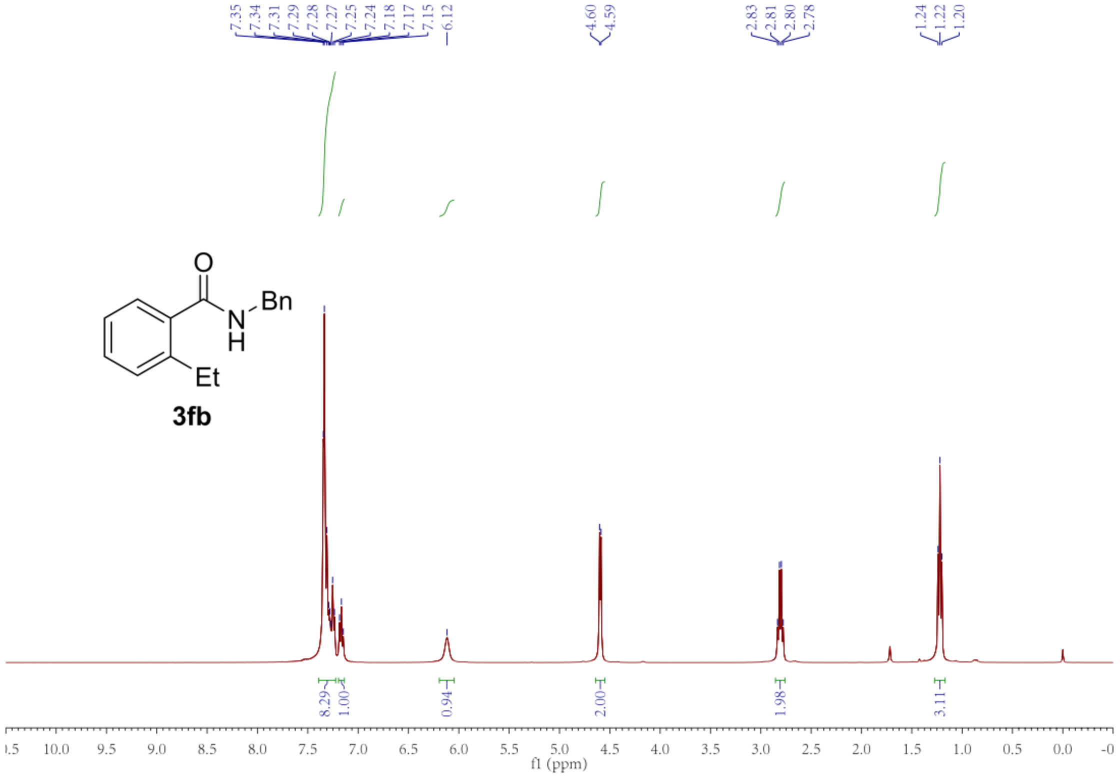

^{13}C NMR (100 MHz, $CDCl_3$)

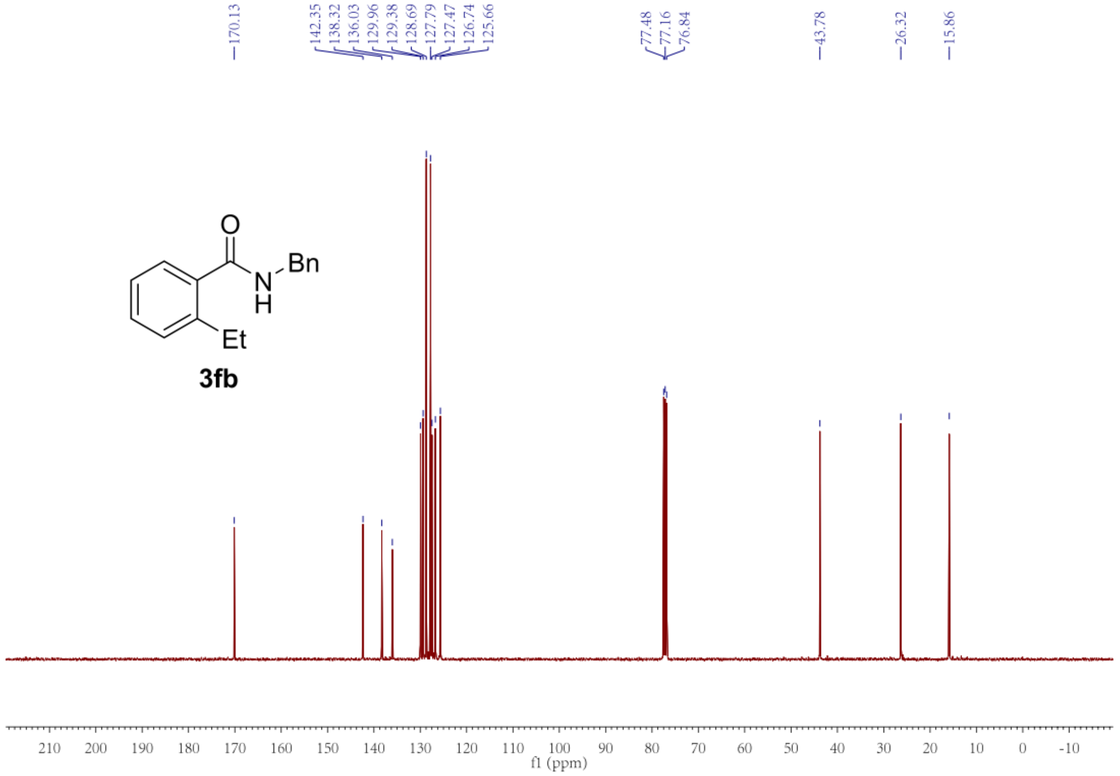

2-ethyl-N-(4-formylbenzyl)benzamide (3gb)

^{1}H NMR (400 MHz, $CDCl_3$)

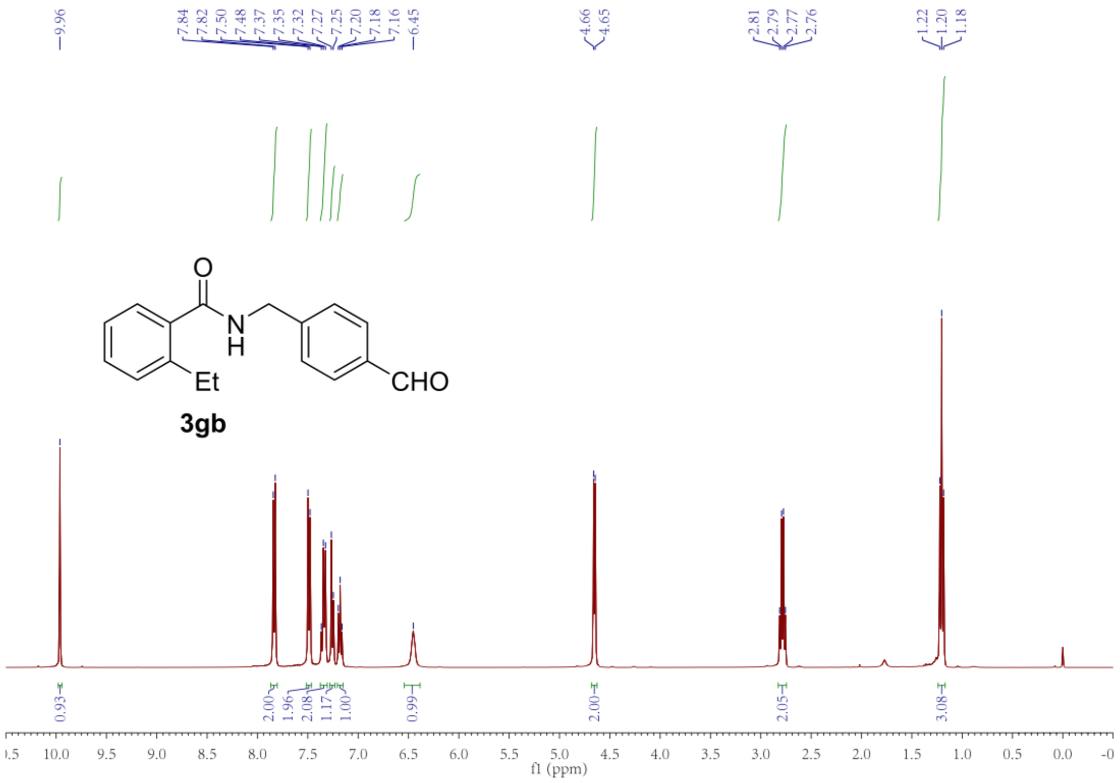

^{13}C NMR (100 MHz, $CDCl_3$)

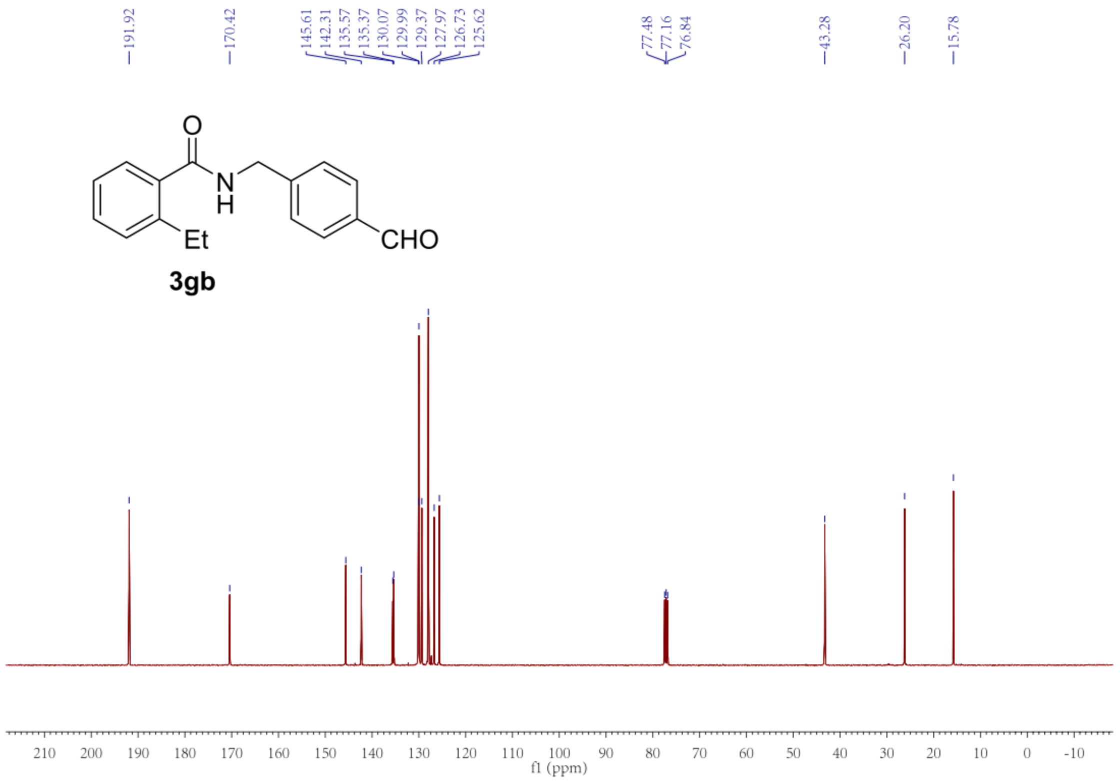

N-(4-cyanobenzyl)-2-ethylbenzamide (3hb)

^{1}H NMR (400 MHz, $CDCl_3$)

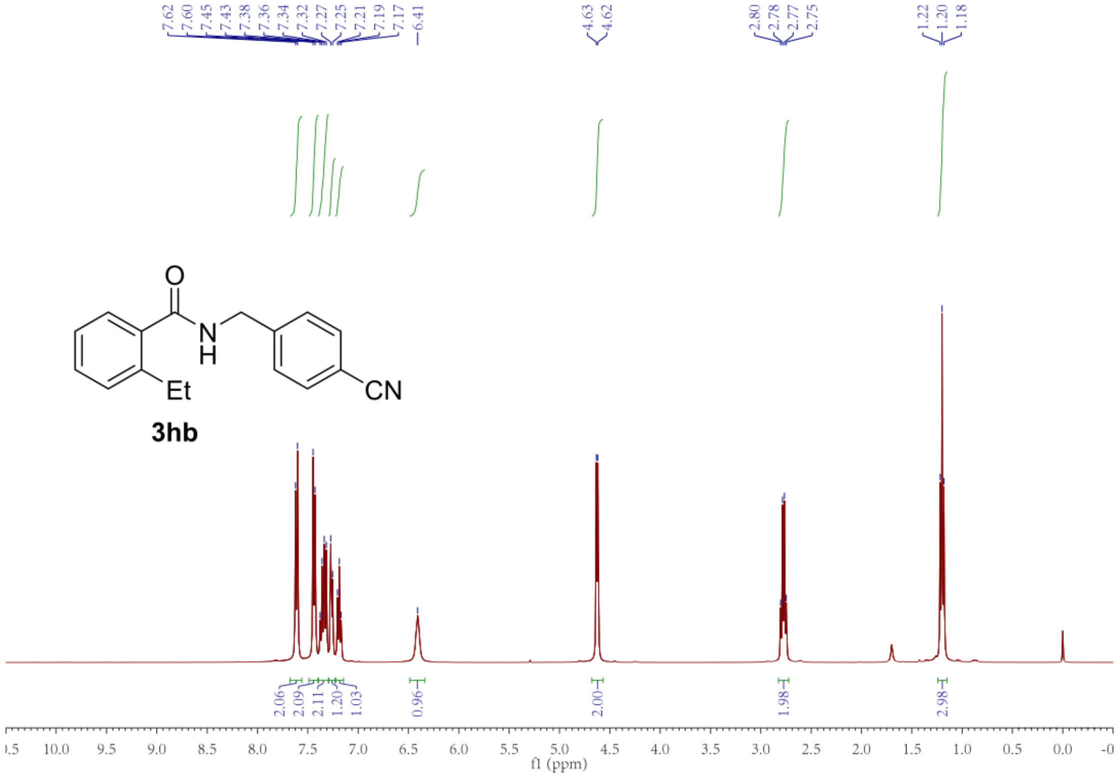

^{13}C NMR (100 MHz, $CDCl_3$)

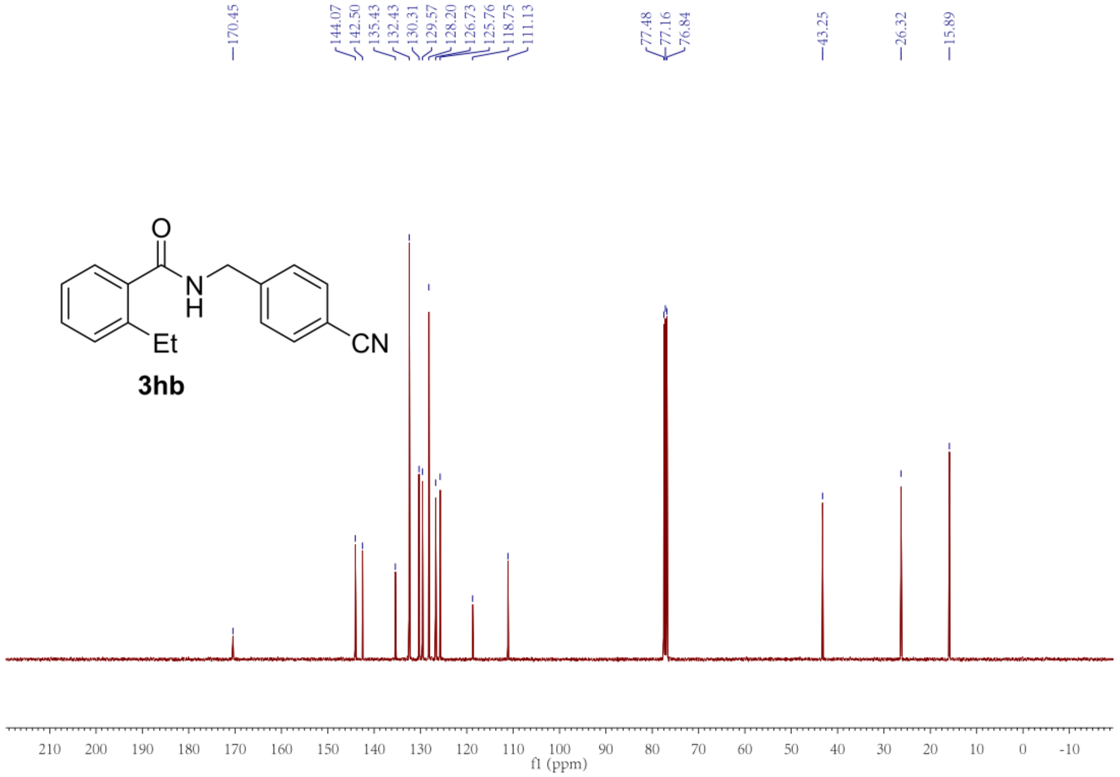

2-ethyl-N-(2,2,2-trifluoroethyl)benzamide (3ib)

^{1}H NMR (400 MHz, $CDCl_3$)

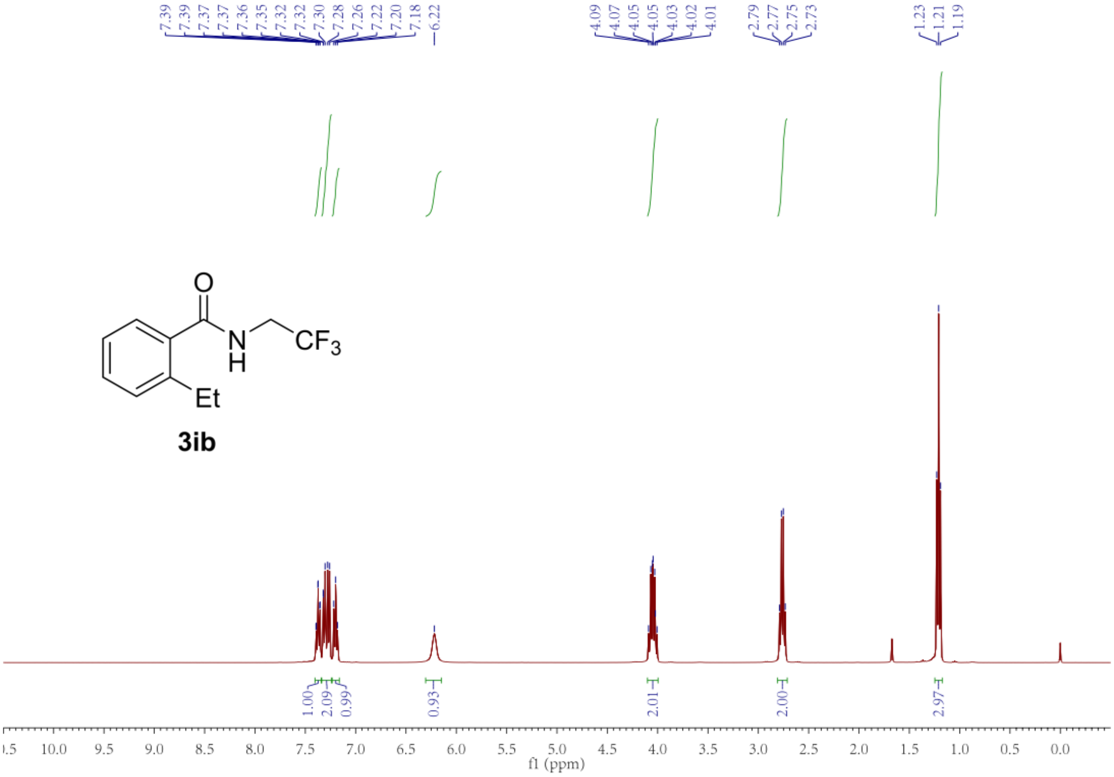

^{13}C NMR (100 MHz, $CDCl_3$)

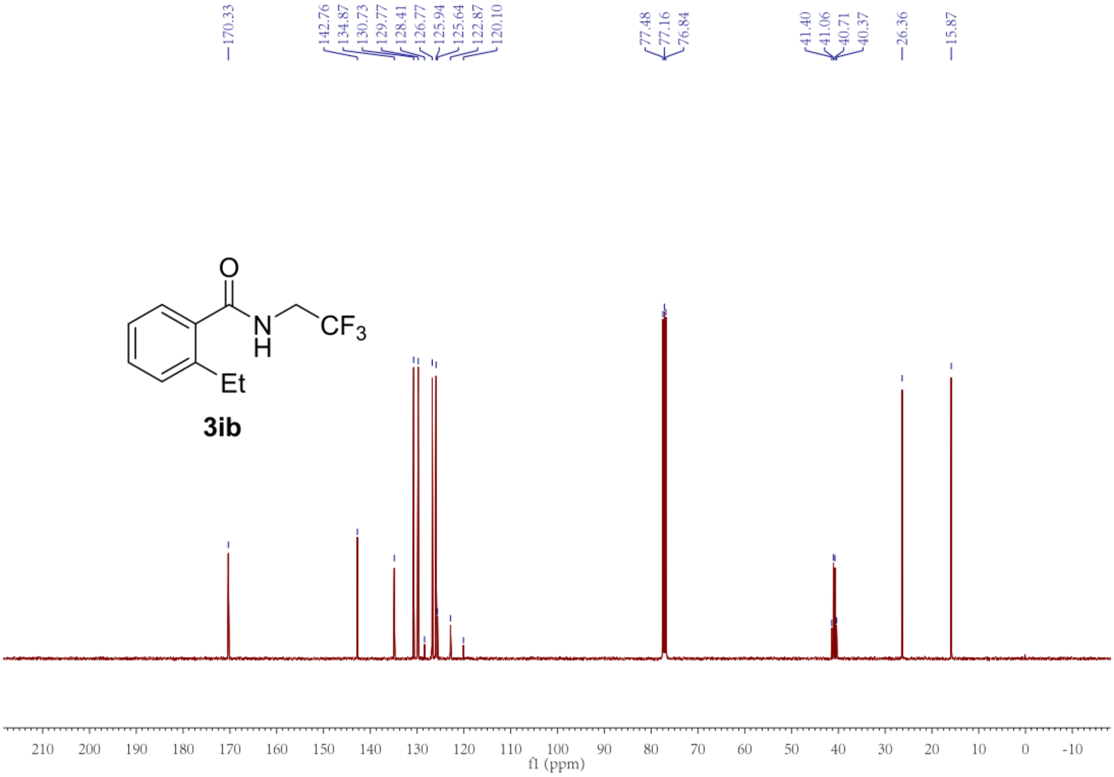

ethyl (2-ethylbenzoyl)glycinate (3jb)

^{1}H NMR (400 MHz, $CDCl_3$)

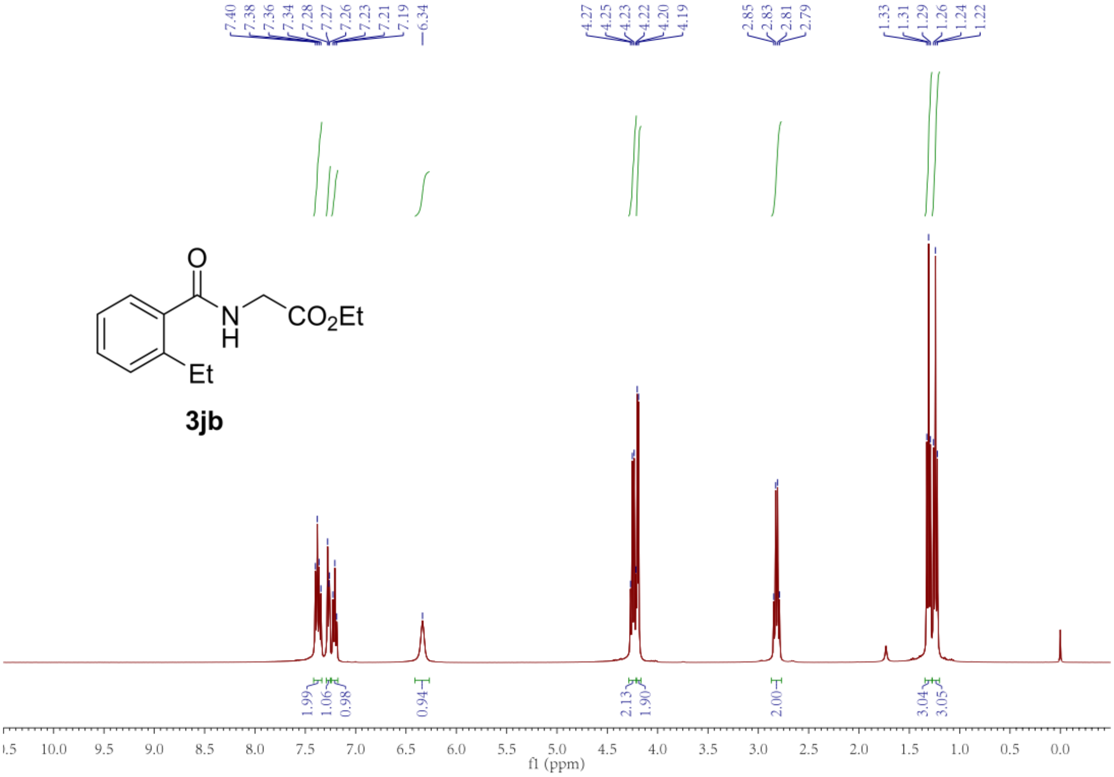

^{13}C NMR (100 MHz, $CDCl_3$)

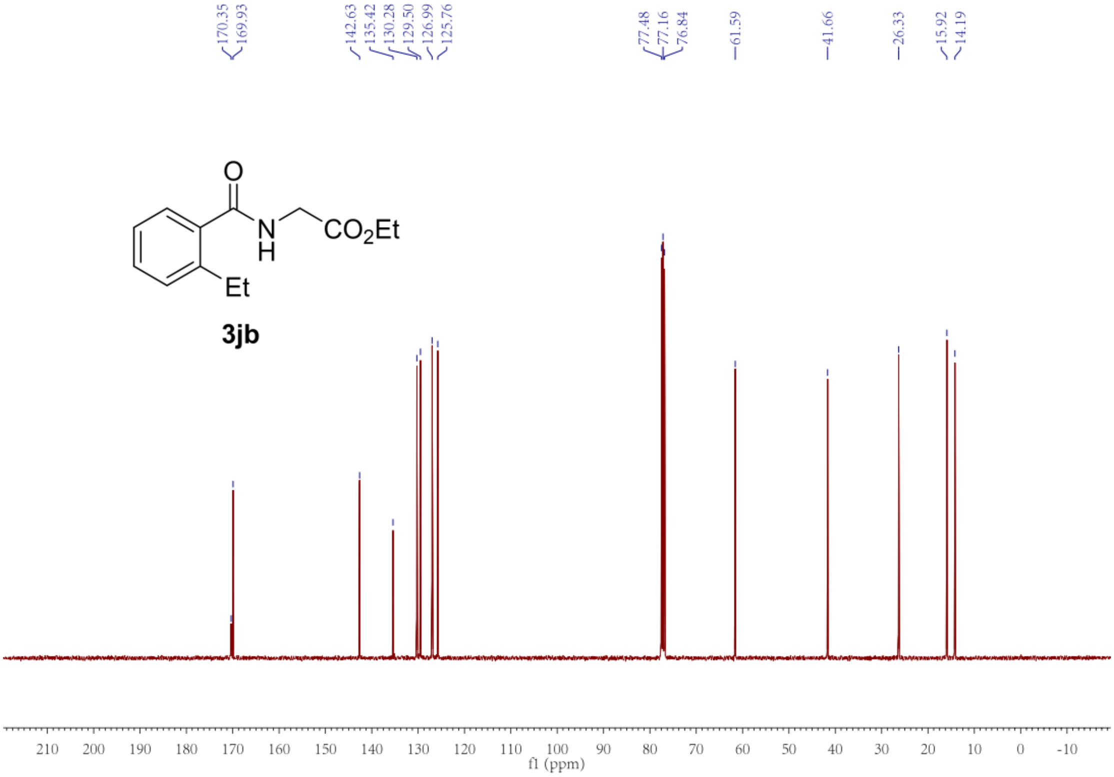

2-ethyl-N-phenylbenzamide (3kb)

^{1}H NMR (400 MHz, $CDCl_3$)

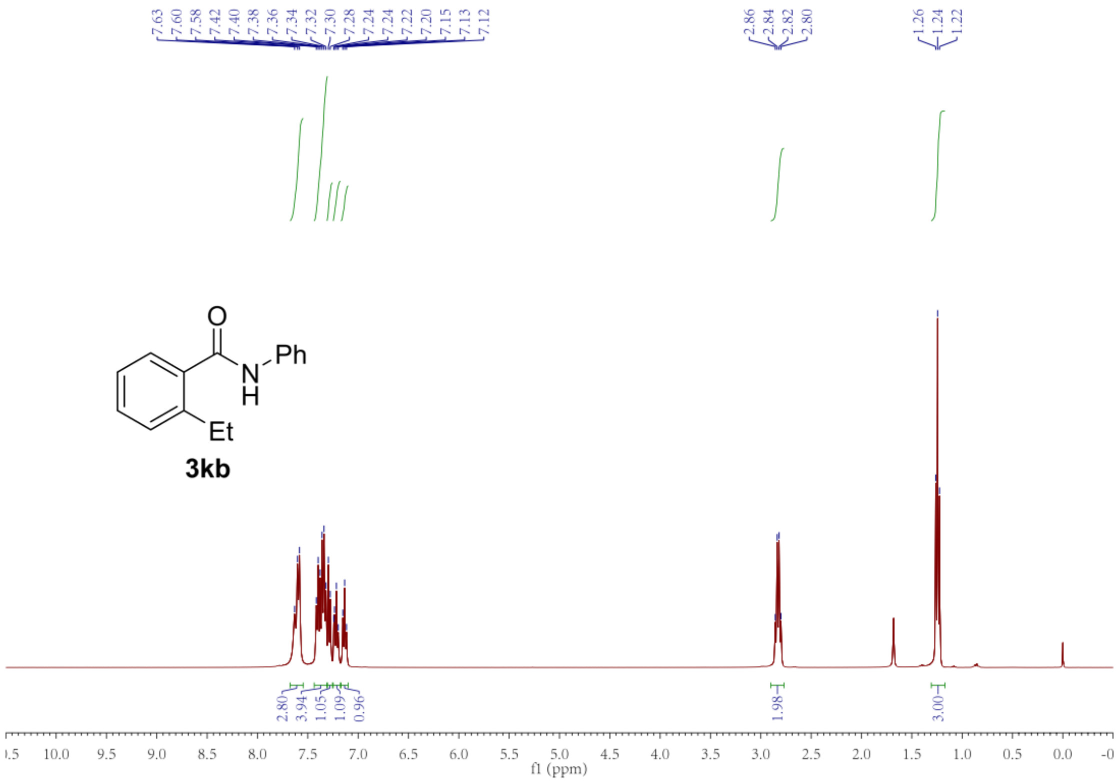

^{13}C NMR (100 MHz, $CDCl_3$)

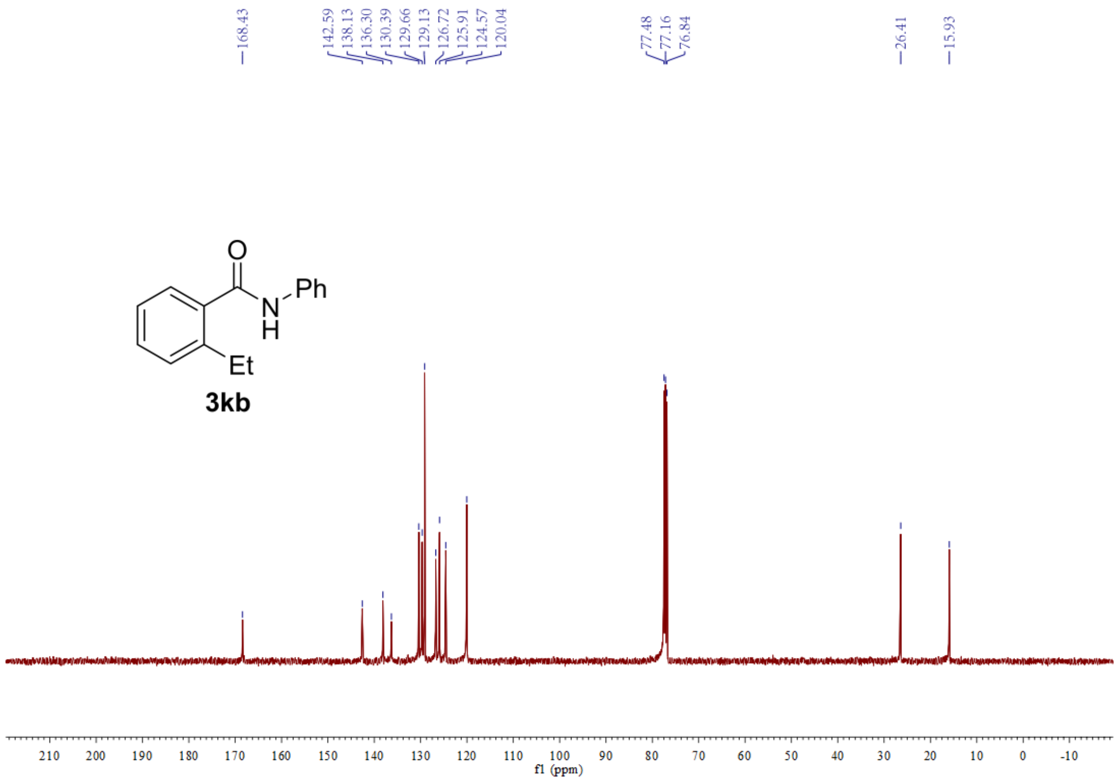

methyl 3-ethyl-4-(methylcarbamoyl)benzoate (3lb)

^{1}H NMR (400 MHz, $CDCl_3$)

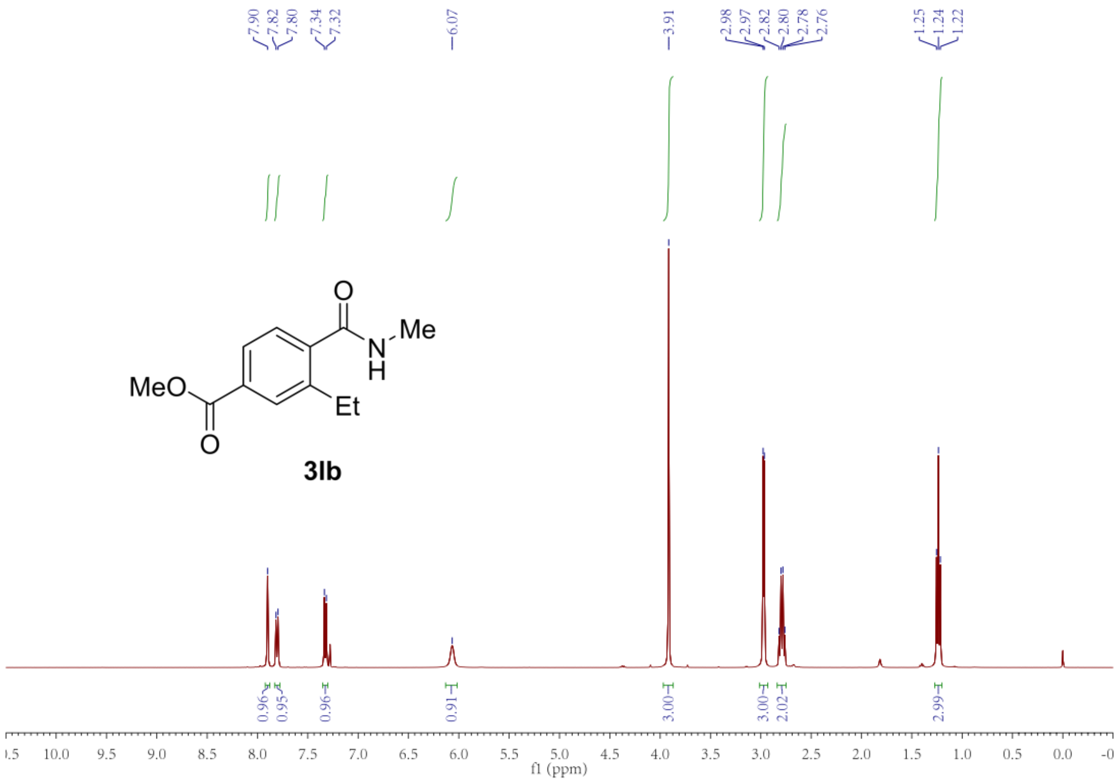

^{13}C NMR (100 MHz, $CDCl_3$)

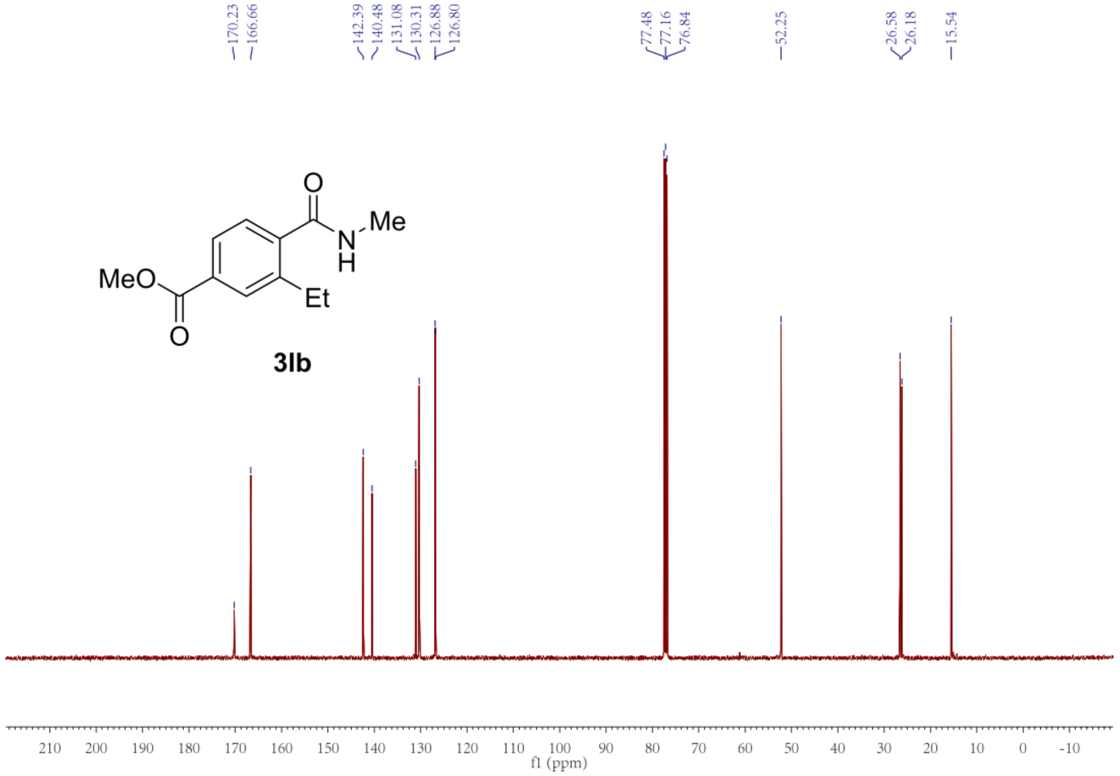

2-ethyl-5-fluoro-N-methylbenzamide (3mb)

^{1}H NMR (400 MHz, $CDCl_3$)

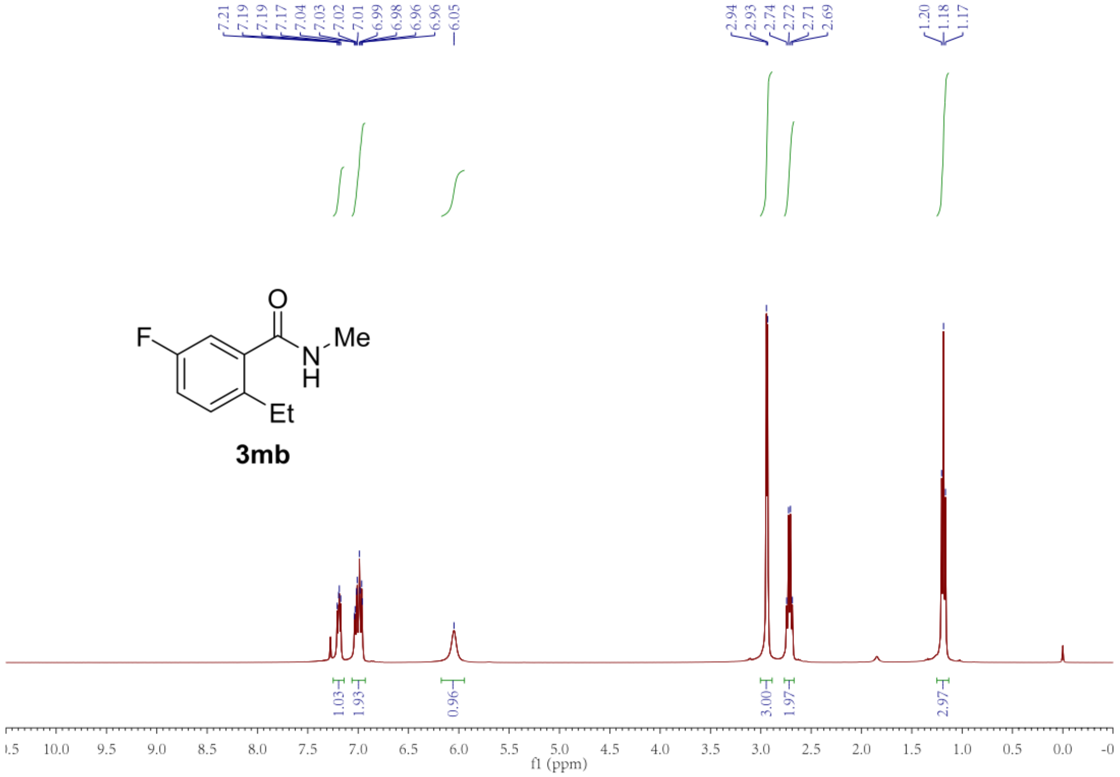

^{13}C NMR (100 MHz, $CDCl_3$)

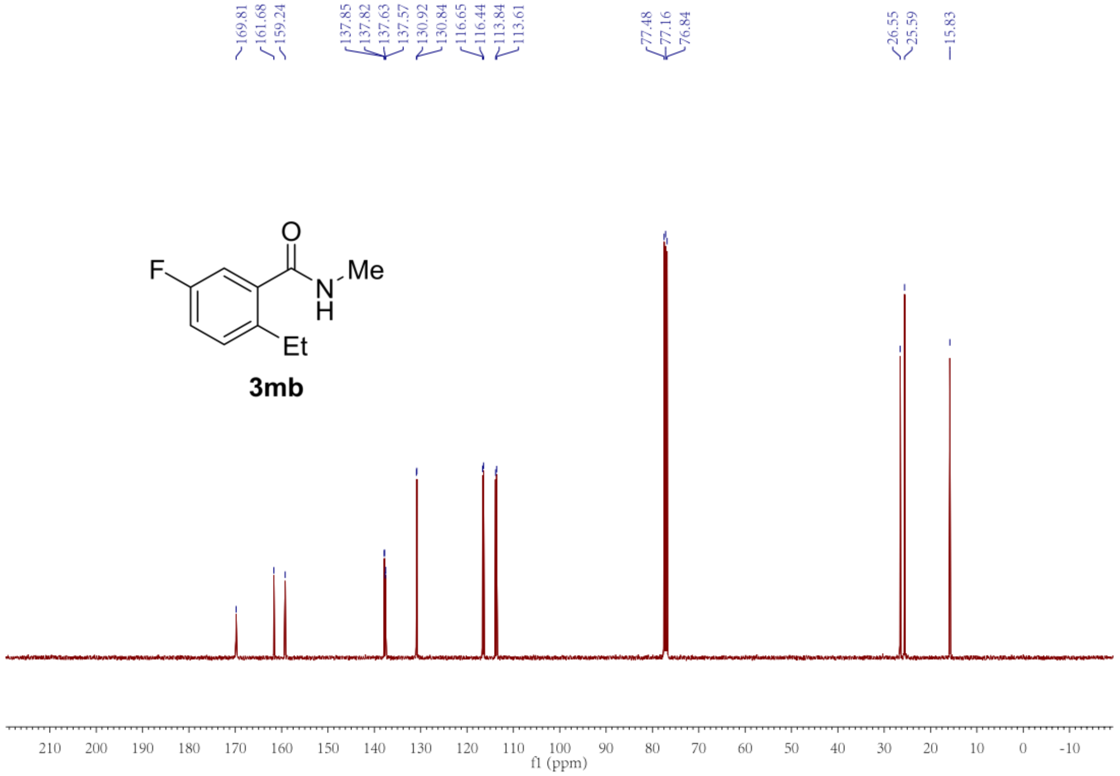

5-chloro-2-ethyl-N-methylbenzamide (3nb)

^{1}H NMR (400 MHz, $CDCl_3$)

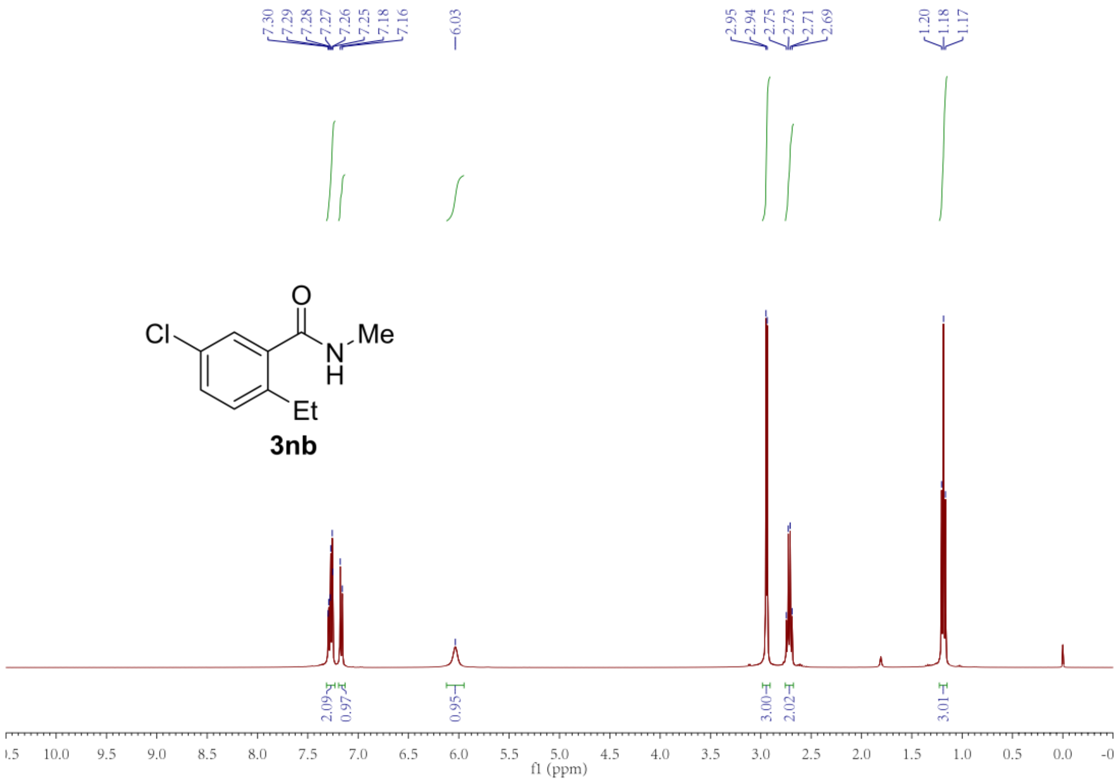

^{13}C NMR (100 MHz, $CDCl_3$)

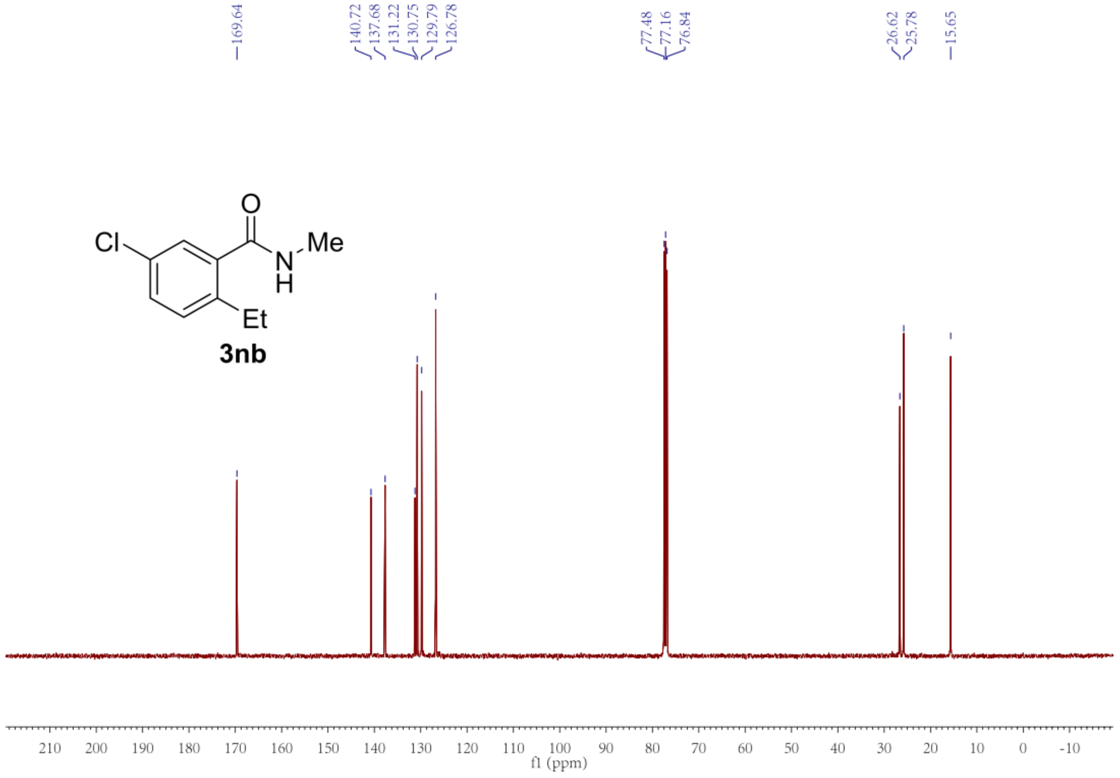

2-ethyl-N,5-dimethylbenzamide (3pb)

^{1}H NMR (400 MHz, $CDCl_3$)

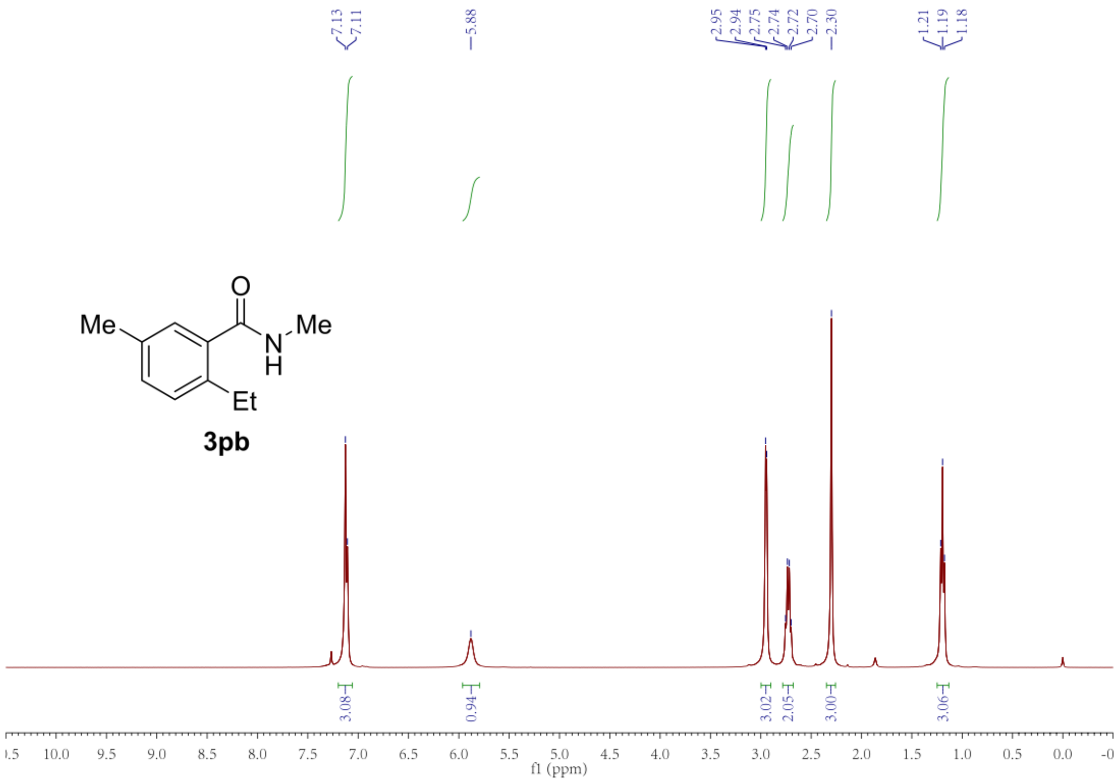

^{13}C NMR (100 MHz, $CDCl_3$)

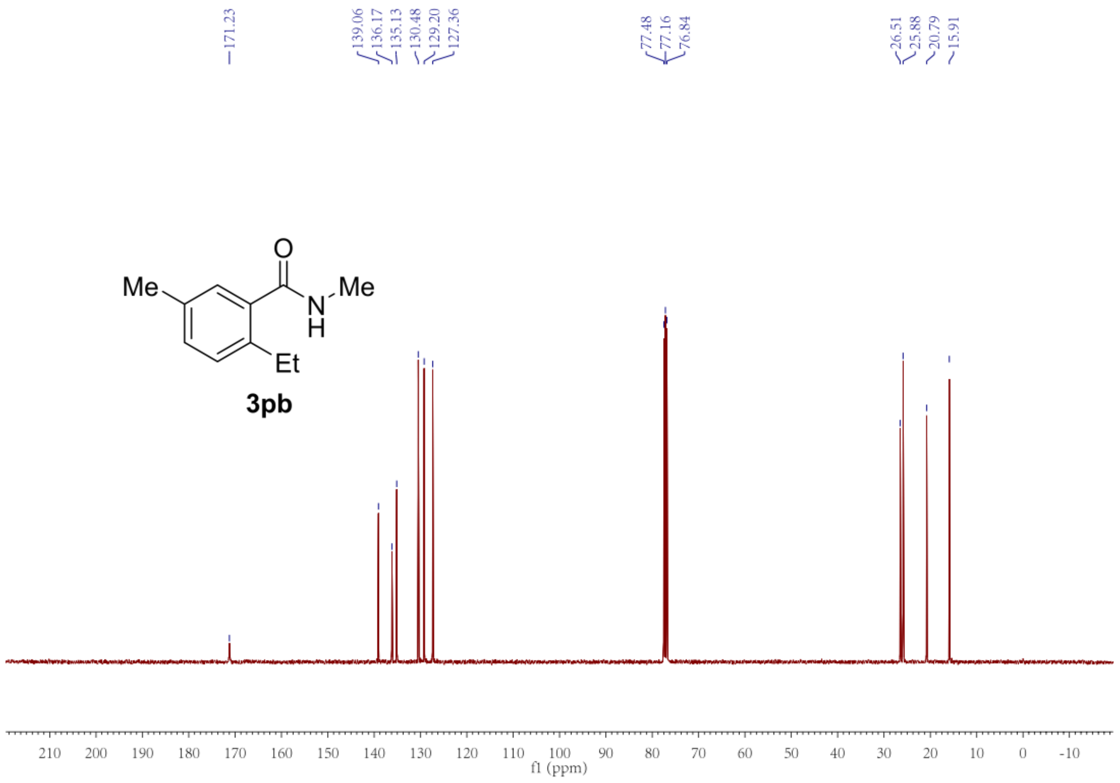

2-ethyl-5-methoxy-N-methylbenzamide (3qb)

^{1}H NMR (400 MHz, $CDCl_3$)

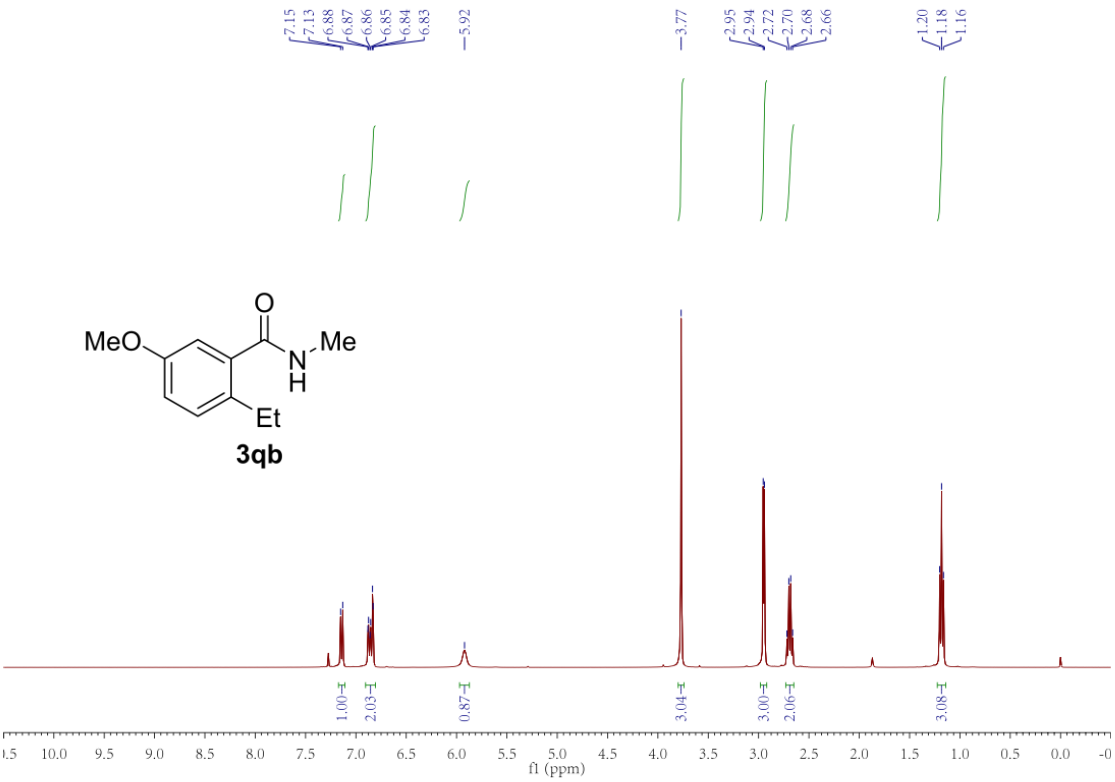

^{13}C NMR (100 MHz, $CDCl_3$)

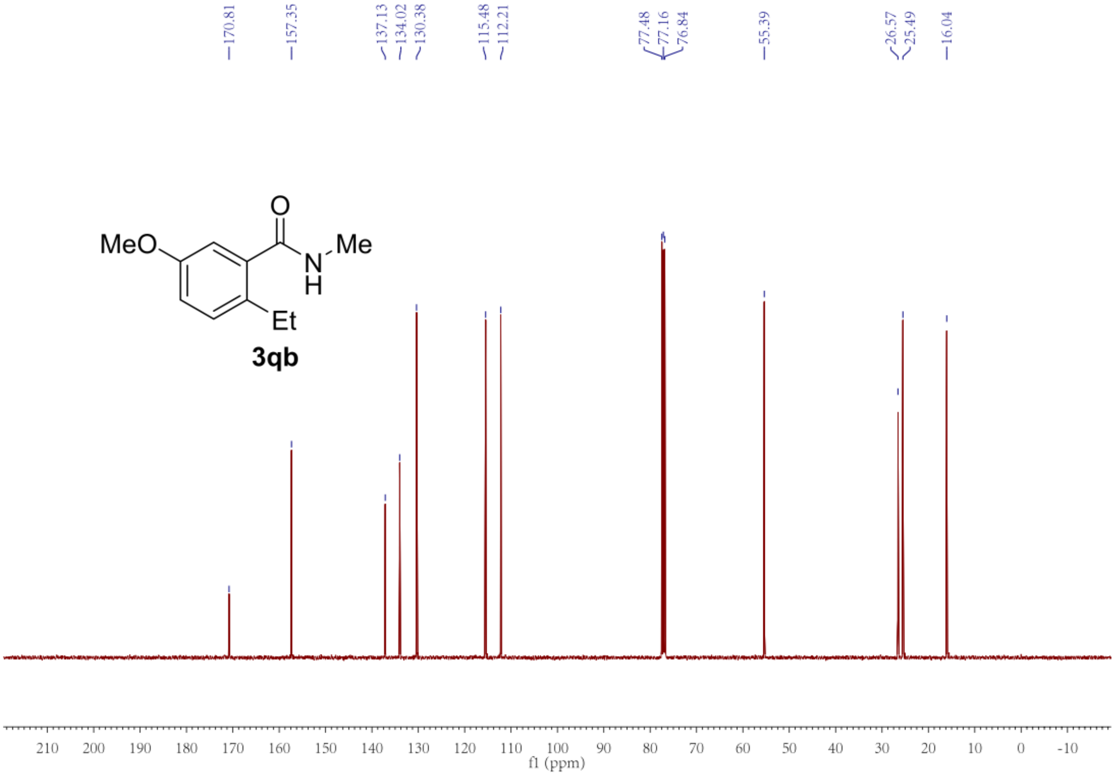

2-ethyl-3-methoxy-N-methylbenzamide (3rb)

^{1}H NMR (400 MHz, $CDCl_3$)

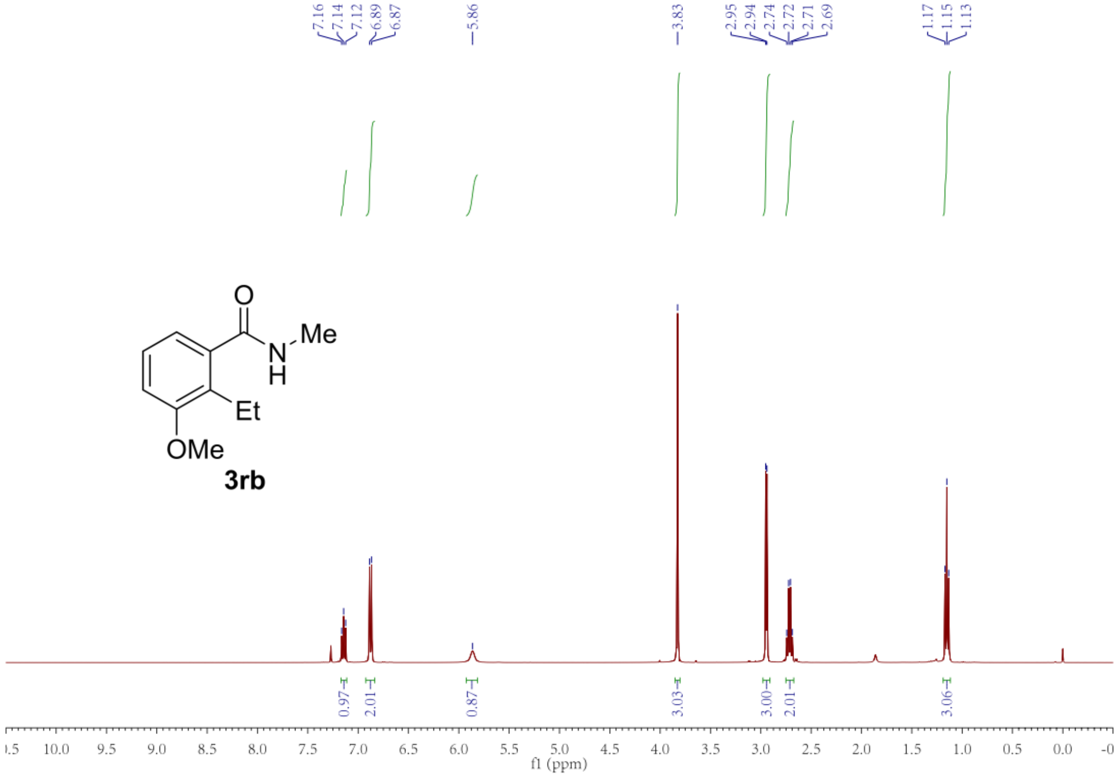

^{13}C NMR (100 MHz, $CDCl_3$)

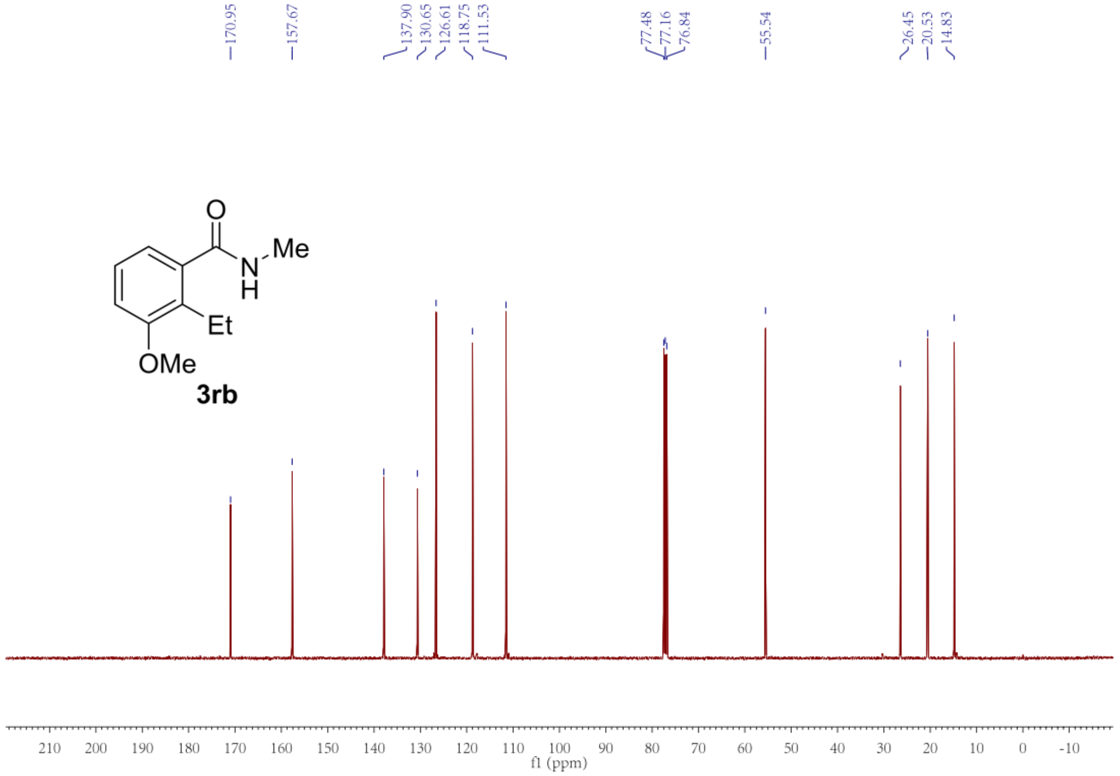

2-ethyl-N-methyl-[1,1'-biphenyl]-3-carboxamide (3sb)

^{1}H NMR (400 MHz, $CDCl_3$)

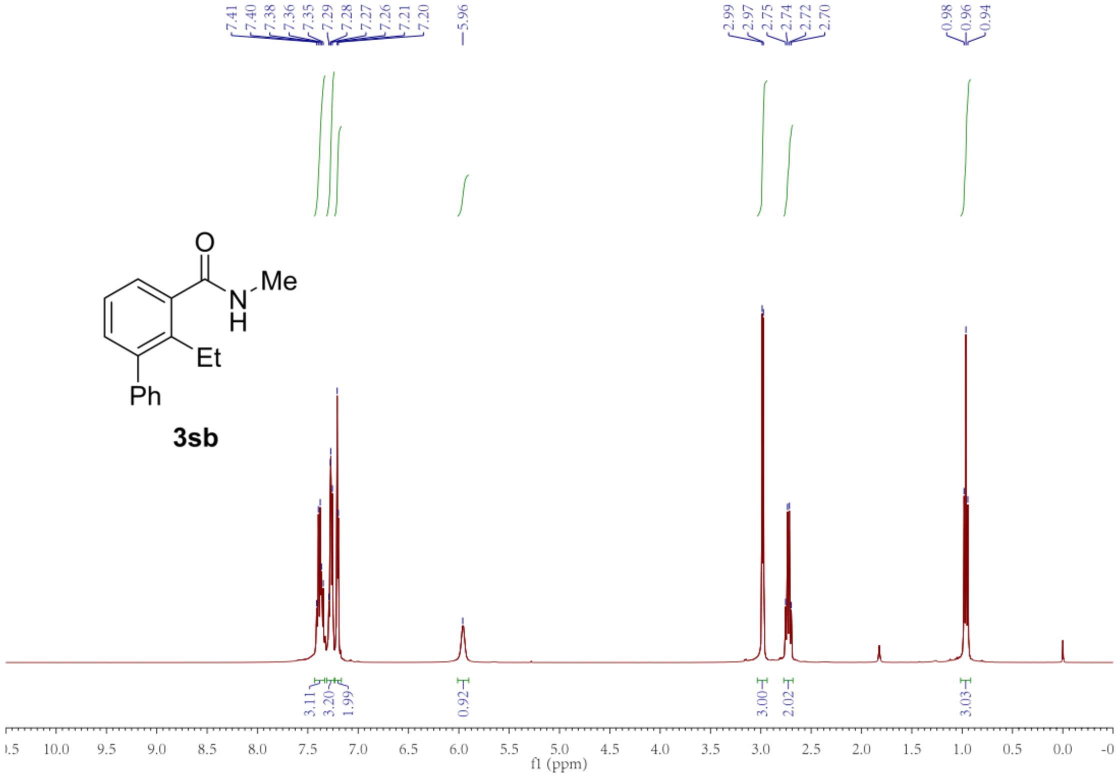

^{13}C NMR (100 MHz, $CDCl_3$)

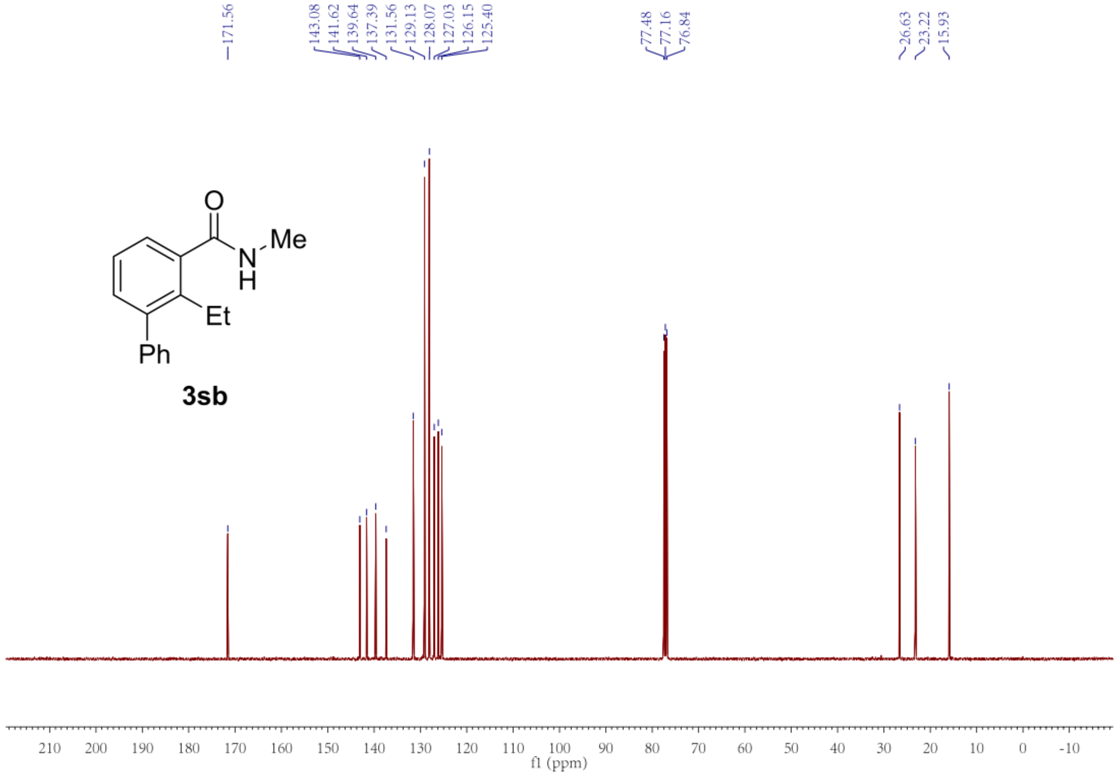

2-ethyl-N,3-dimethylbenzamide (3tb)

^{1}H NMR (400 MHz, $CDCl_3$)

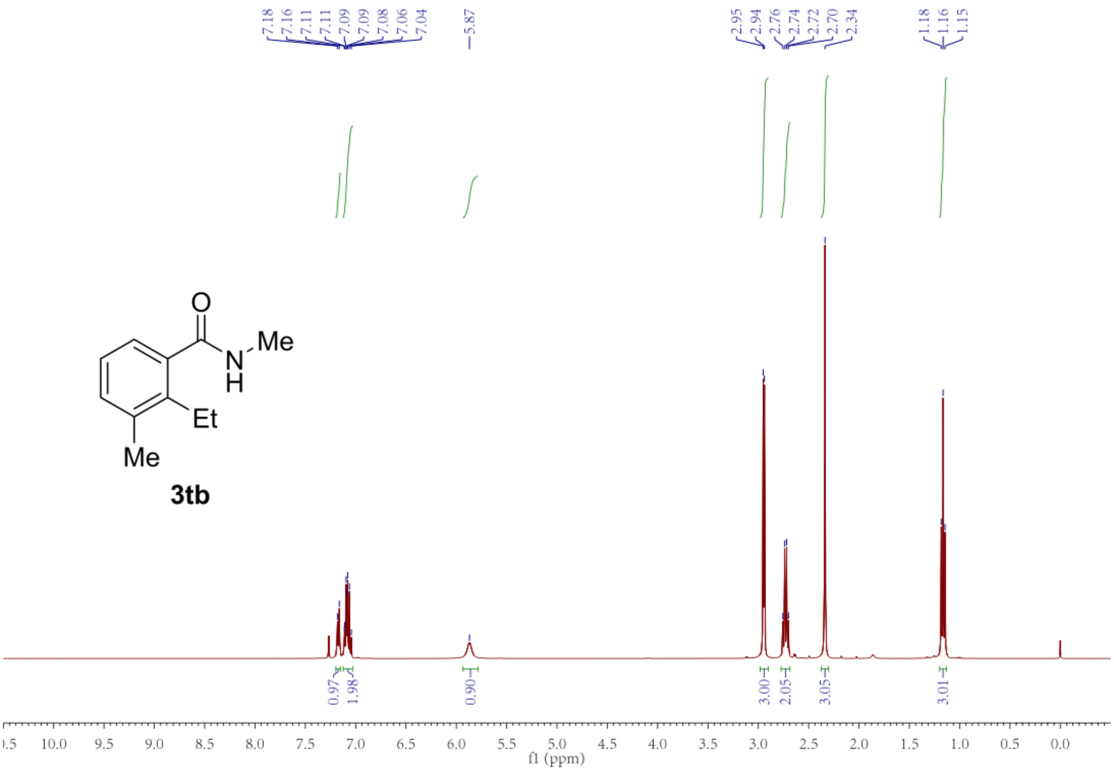

^{13}C NMR (100 MHz, $CDCl_3$)

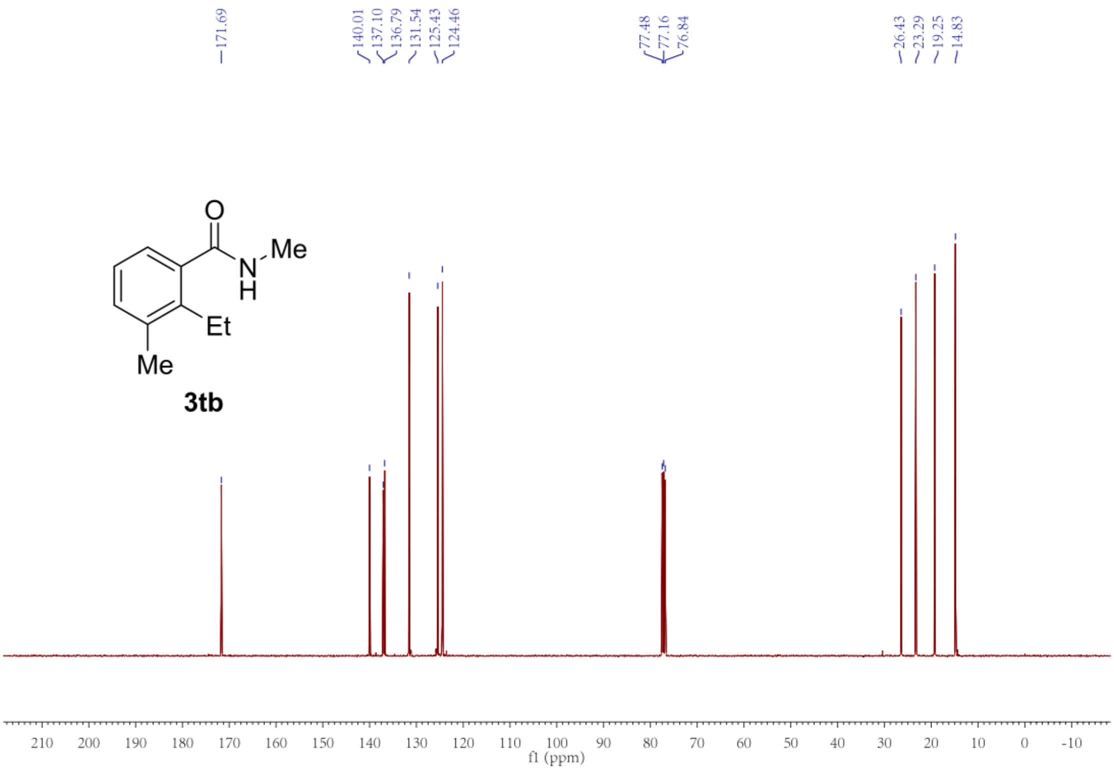

3. ^{19}F NMR spectra

2-ethyl-N-(2,2,2-trifluoroethyl)benzamide (3ib)

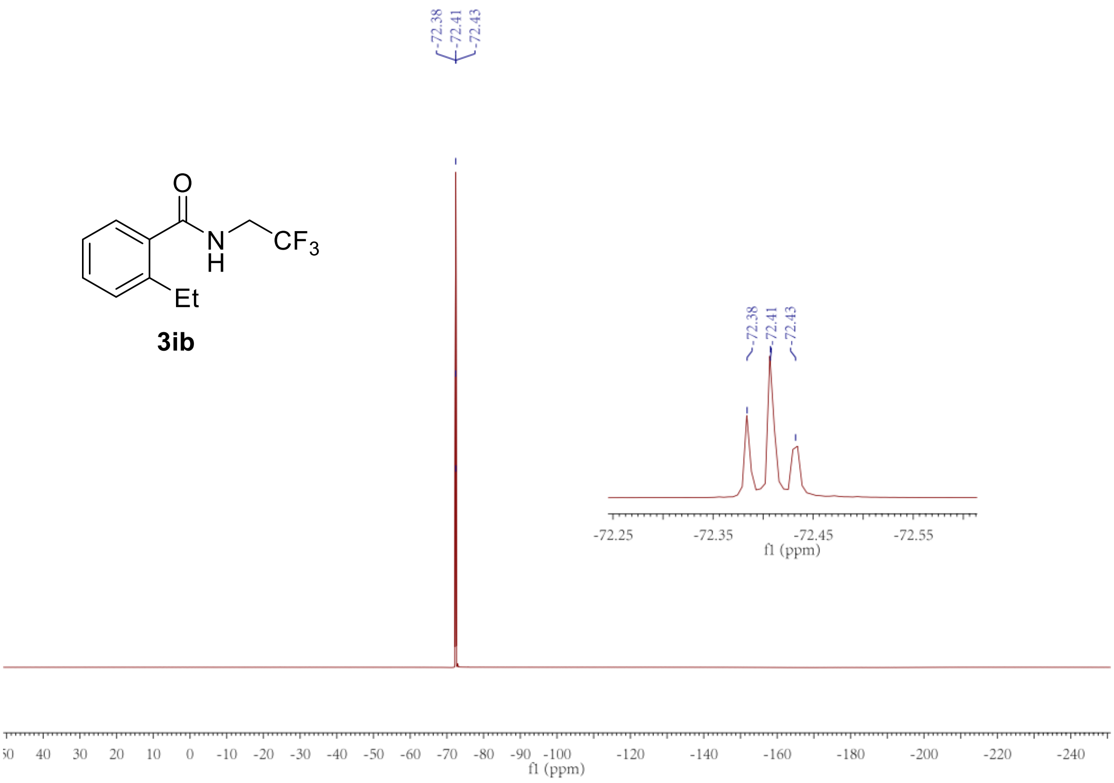

2-ethyl-5-fluoro-N-methylbenzamide (3mb)

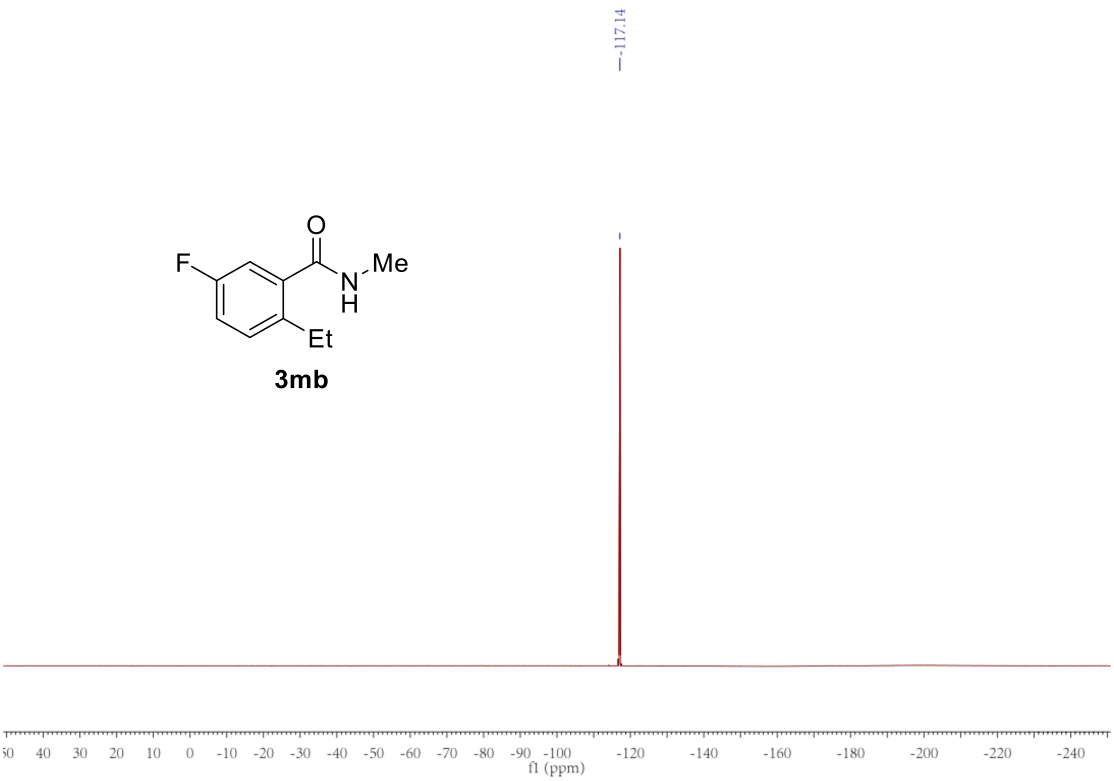

4. ^{1}H NMR and GC-MS of crude product with 2a

1) ^{1}H NMR of crude product with 1.5 equiv. MeOTs using 10 mol%$NiCl_2$(bpy) at 100℃

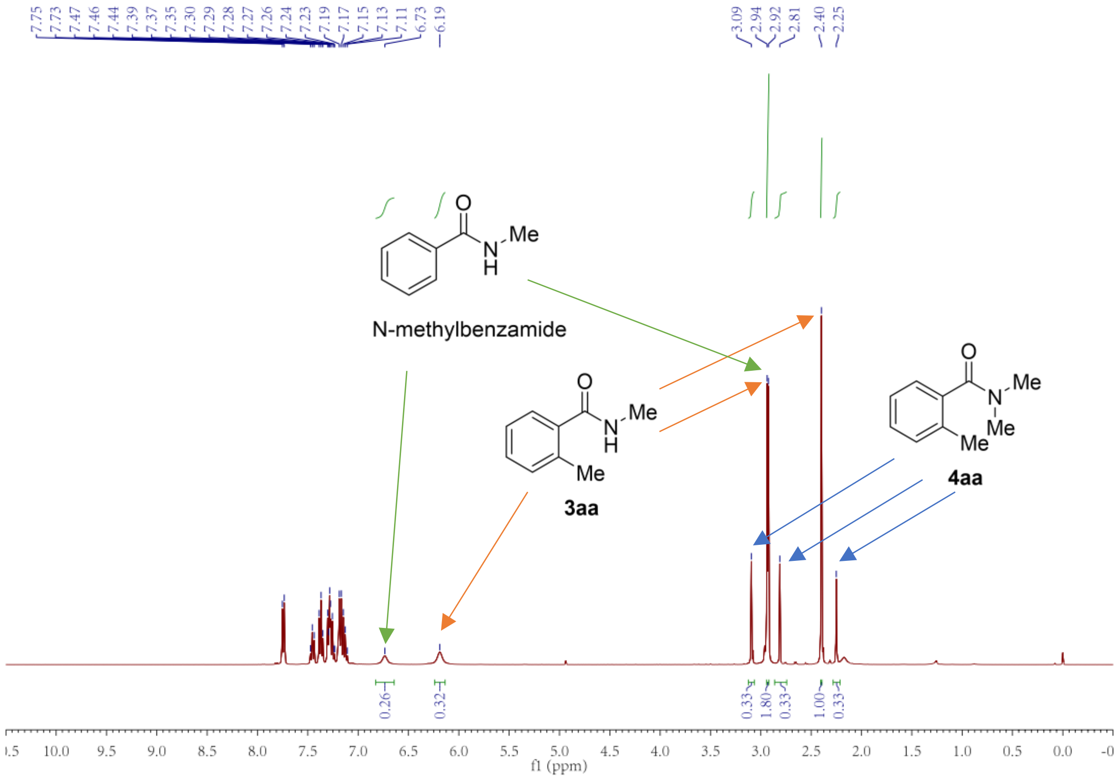

2) ^{1}H NMR of crude product with 1.5 equiv. MeOTs using 10 mol%$NiCl_2$(bpy) at 80℃

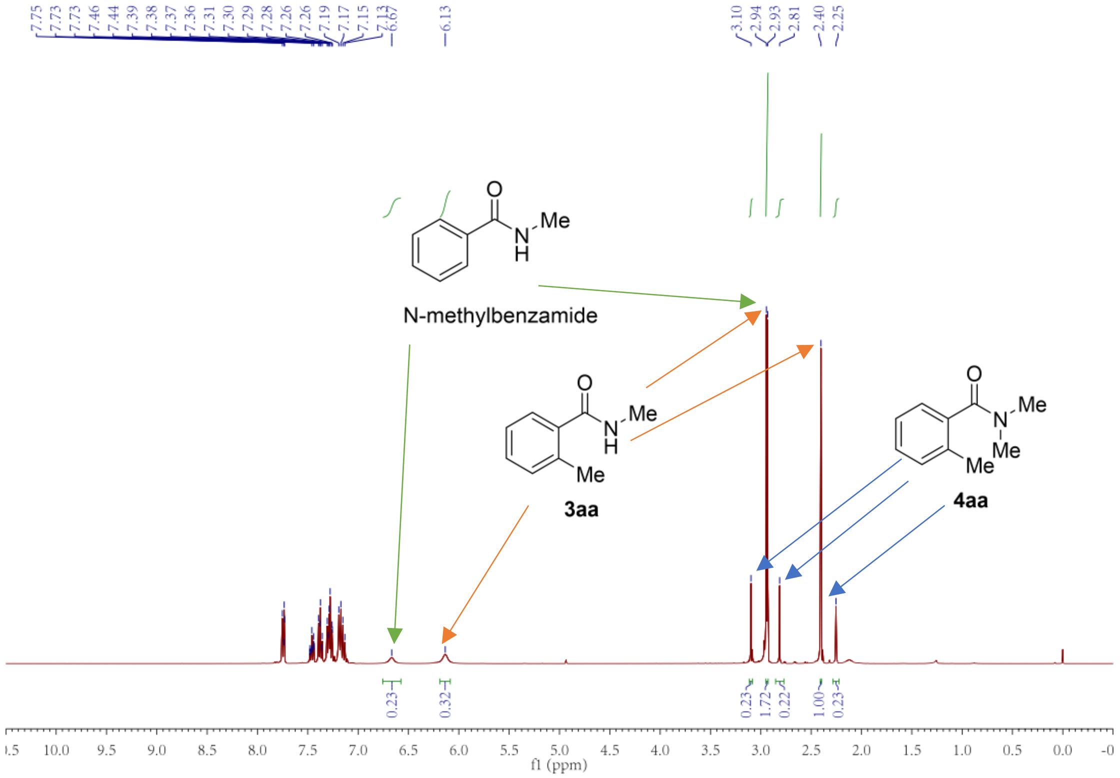

3) GC-MS of crude product with 1.5 equiv. MeOTs using 10 mol%$NiCl_2$(bpy) at 100℃

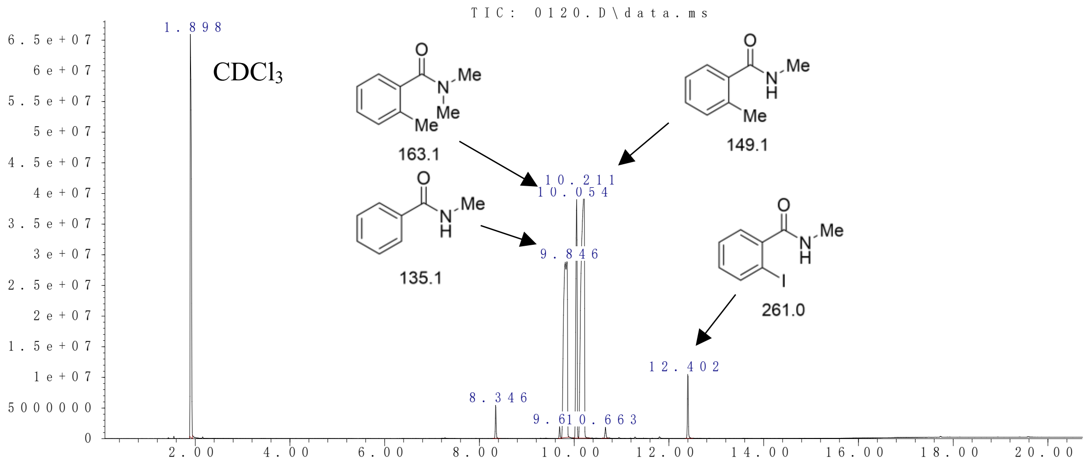

Data Path : D:\2025DATA\

Data File : 0120.D

Acq On : 20 Feb 2025 19:51

Operator : T-all

Sample : HYY

Misc :

ALS Vial: 3 Sample Multiplier: 1

Integration Parameters : autoint1.e

Integrator : ChemStation

Method : D:\GCMS Methods

Title :

Signal：TIC: 0120.D\data.ms

peak #	R.T. min	first scan	max scan	last scan	PK TY	peak height	corr. area	corr. % max	%of total
1	8.346	1379	1389	1402	BB	5333850	67971933	3.38%	1.477%
2	9.695	1610	1616	1622	BV	1831341	24271445	1.21%	0.527%
3	9.846	1623	1641	1650	M2	28661590	1588539139	79.02%	34.511%
4	10.054	1663	1676	1682	BV 2	38263000	744577671	37.04%	16.176%
5	10.211	1682	1702	1723	VB 2	40890476	2010182209	100.00%	43.671%
6	10.663	1764	1778	1788	BV 2	1727437	27920854	1.39%	0.607%
7	12.402	2062	2071	2086	BB	10470817	139516418	6.94%	3.031%

Sum of corrected areas： 4602979668

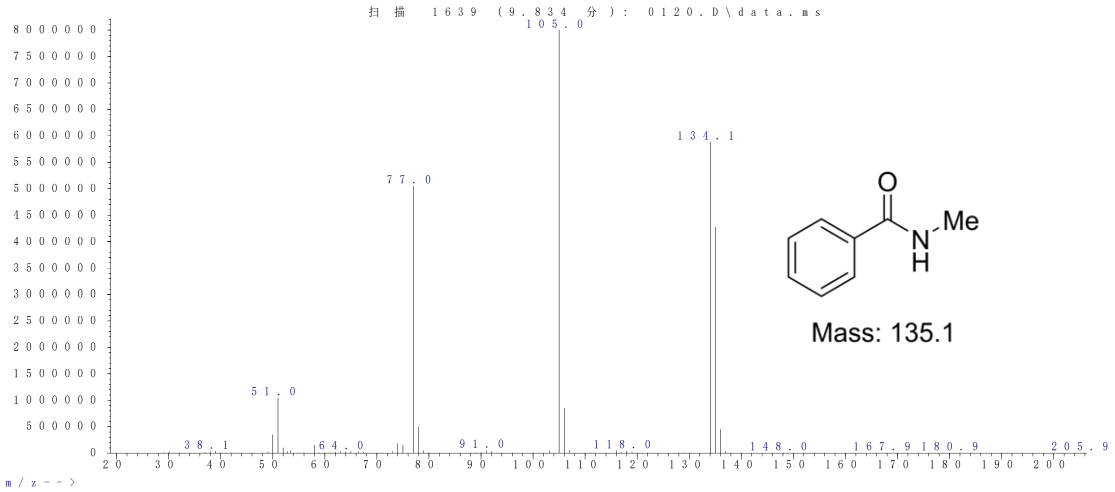

扫描 1639 (9.834 分): 0120.D\data.ms
105.0
134.1
77.0
51.0
38.1
64.0
91.0
118.0
148.0
167.9
180.9
205.9
m/z-->
Me
N
H
O
Mass: 135.1

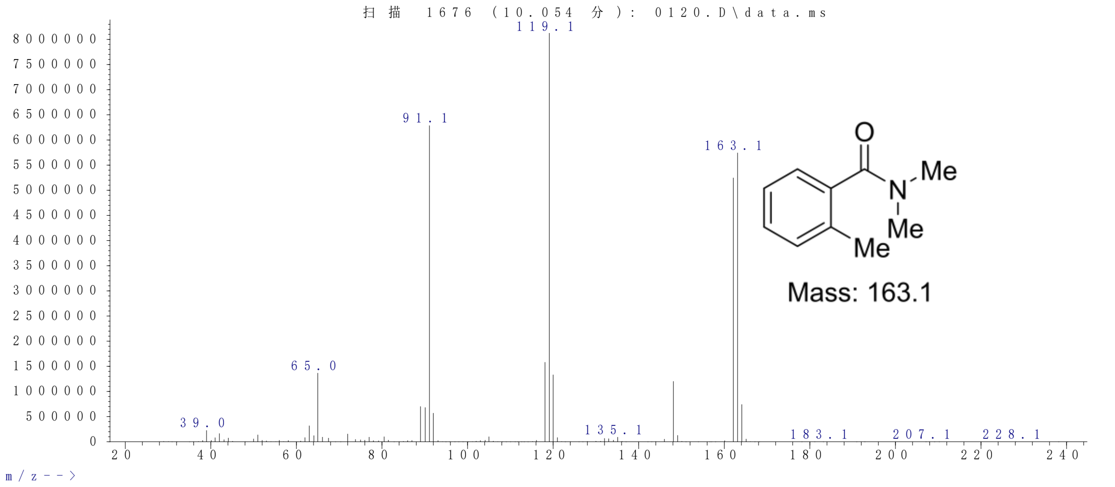

扫描 1676 (10.054 分): 0120.D\data.ms
119.1
91.1
163.1
65.0
39.0
135.1
183.1
207.1
228.1
m/z-->
O
N
Me
Me
Me
Mass: 163.1

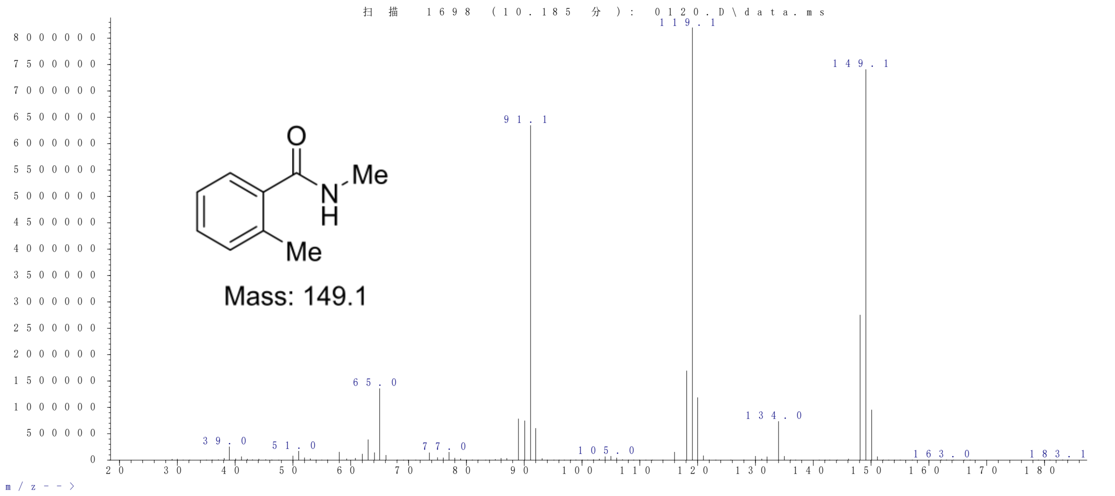

扫描 1698 (10.185 分): 0120.D\data.ms
119.1
149.1
91.1
65.0
134.0
39.0
51.0
77.0
105.0
163.0
183.1
m/z-->
O
N
H
Me
Me
Mass: 149.1

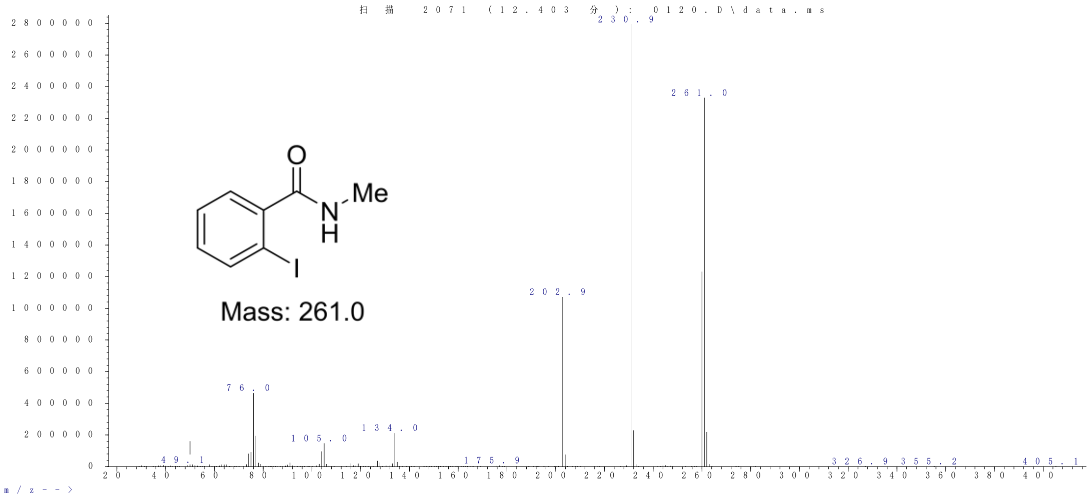
扫描 2071 (12.403 分): 0120.D\data.ms
230.9
261.0
202.9
76.0
134.0
105.0
49.1
175.9
326.9
355.2
405.1
Mass: 261.0
m/z-->

5. ¹H NMR and GC-MS of the XEC product (Cl/Br/I scrambled) with 2e

1) ¹H NMR of the XEC product (Cl/Br/I scrambled) with **2e**

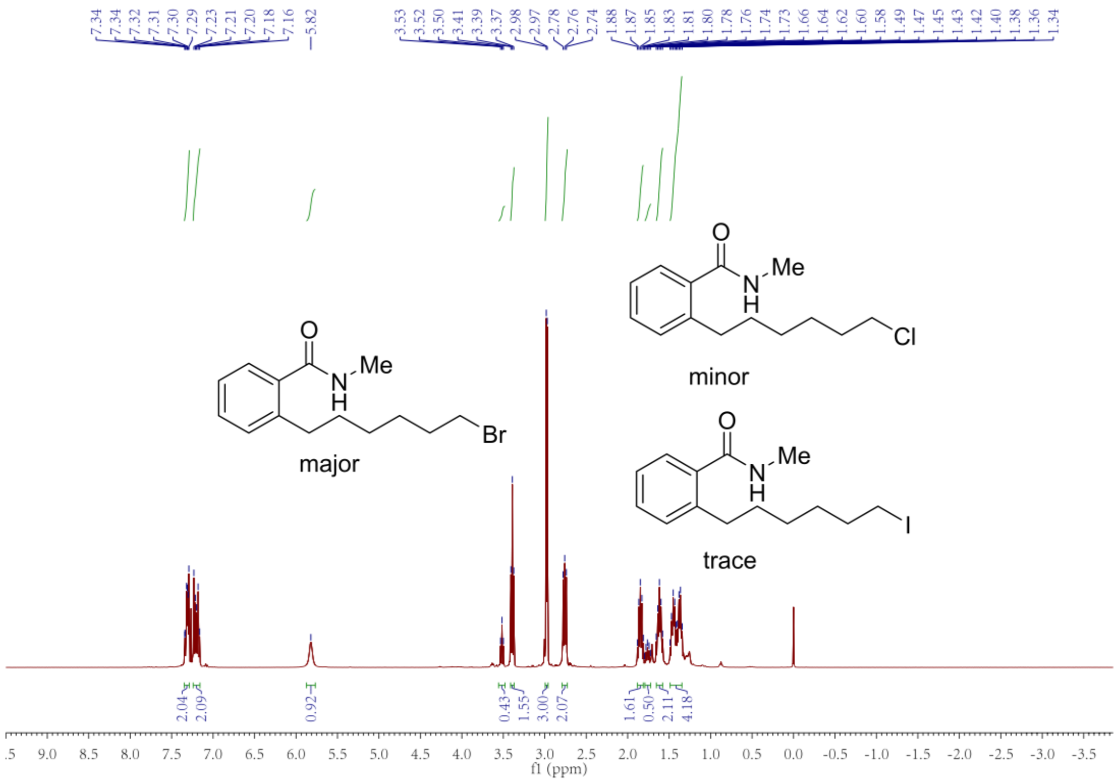

2) GC-MS of the XEC product (Cl/Br/I scrambled) with **2e**

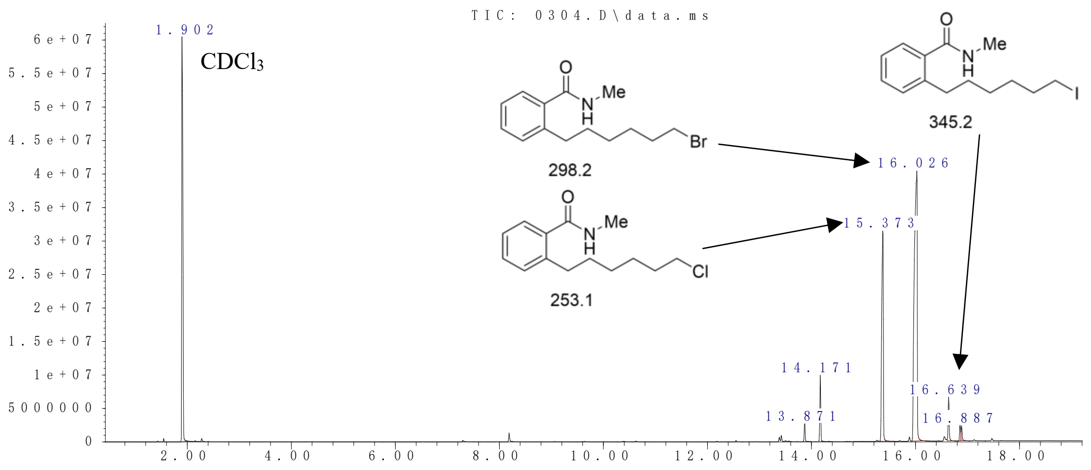

Data Path : D:\2025DATA\

Data File : 0304.D

Acq On : 11 Mar 2025 16:44

Operator :

Sample : HYY-6-Br

Misc :

ALS Vial: 3 Sample Multiplier: 1

Integration Parameters : autoint1.e

Integrator : ChemStation

Method : D:\GCMS Methods- 1.M

Title :

Signal：TIC: 0304.D\data.ms

peak #	R.T. min	first scan	max scan	last scan	PK TY	peak height	corr. area	corr. % max	%of total
---	-----	-----	----	----	---	-------	-------	------	-------
1	13.871	2308	2318	2327	BB	2625220	34118576	2.20%	1.340%
2	14.171	2359	2368	2379	BB	9554905	126751450	8.16%	4.977%
3	15.373	2561	2571	2588	BB	31680191	681471549	43.89%	26.760%
4	16.026	2663	2680	2702	VB	40768135	1552696072	100.00%	60.970%
5	16.639	2778	2783	2795	VB	6255396	87317182	5.62%	3.429%
6	16.858	2814	2820	2823	BV	2267586	31196274	2.01%	1.225%
7	16.887	2823	2825	2835	VB	2167297	33097098	2.13%	1.300%

Sum of corrected areas： 2546648201

1.M Wed Mar 21:34:37 2025

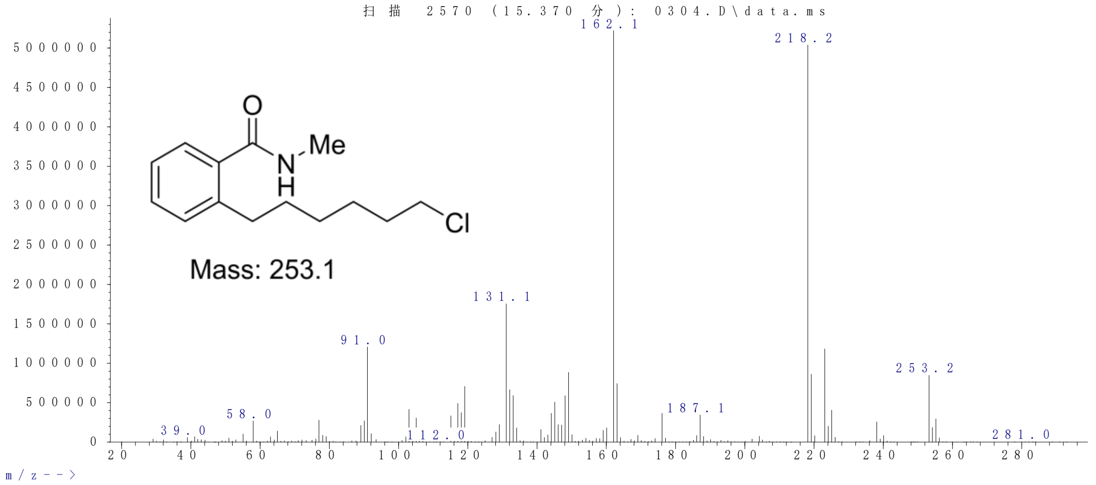

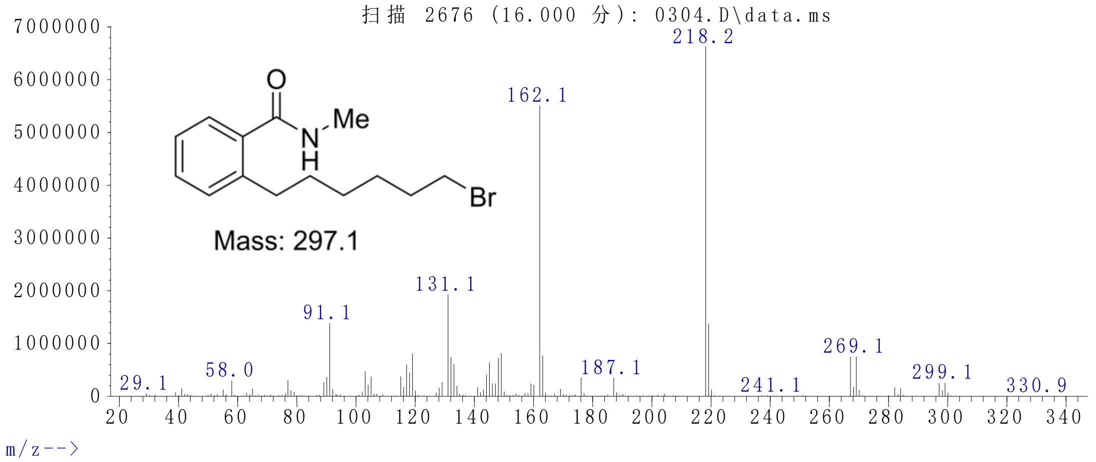

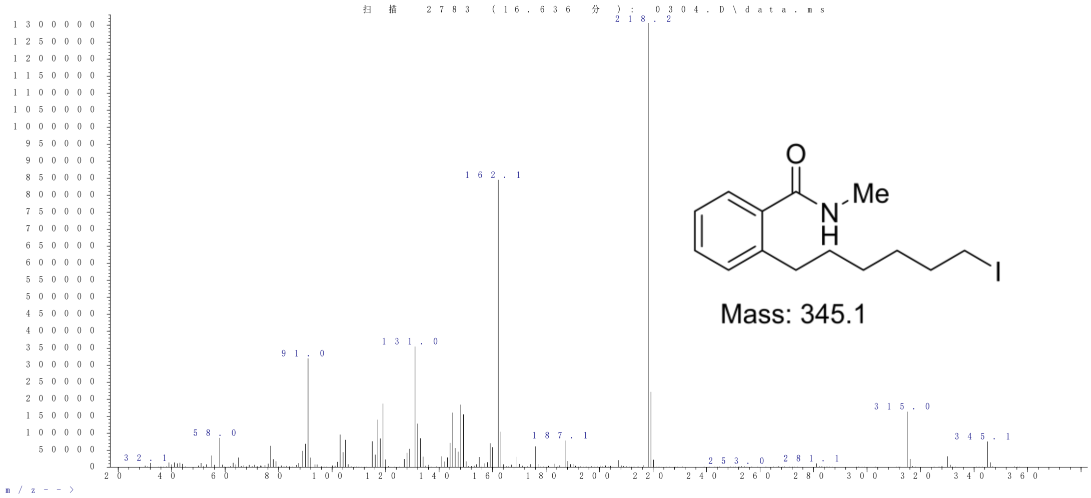

扫描 2783 (16.636 分): 0304.D\data.ms
218.2
162.1
131.0
91.0
58.0
32.1
187.1
253.0
281.1
315.0
345.1
m/z-->
O
N
H
Me
I
Mass: 345.1

6.GC-MS of 3ah

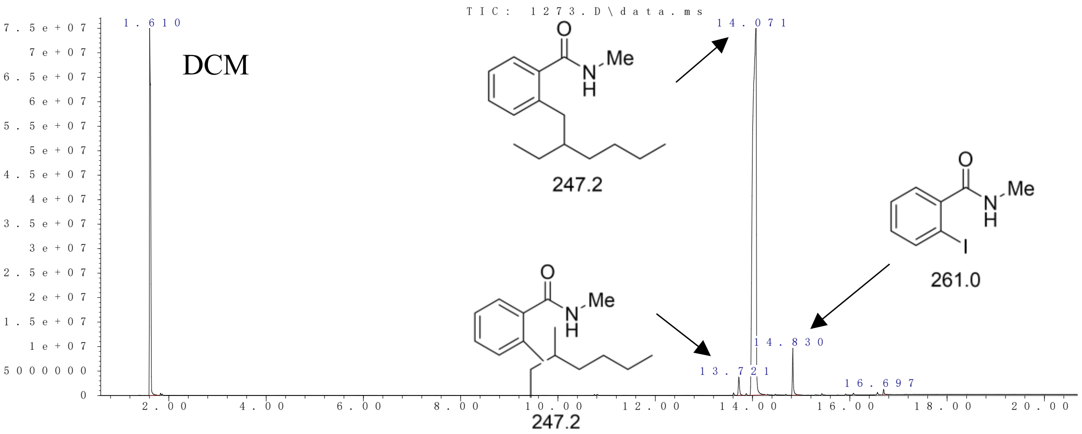

Data Path : D:\2025DATA\

Data File : 1273.D

Acq On : 22 May 2025 00:54

Operator :

Sample : HYY

Misc :

ALS Vial: 1 Sample Multiplier: 1

Integration Parameters : autoint1.e

Integrator : ChemStation

Method : D:\GCMS Methods- 1.M

Title :

Signal：TIC: 1273.D\data.ms

peak #	R.T. min	first scan	max scan	last scan	PK TY	peak height	corr. area	corr. % max	%of total
1	13.718	2289	2292	2299	M	3801173	65082406	1.68%	1.605%
2	14.071	2329	2352	2388	BV	77568000	3864521433	100.00%	95.292%
4	16.695	2789	2793	2796	M	1032304	11151133	0.29%	0.275%

Sum of corrected areas： 4055439152

1.M Tues May 10:00:29 2025

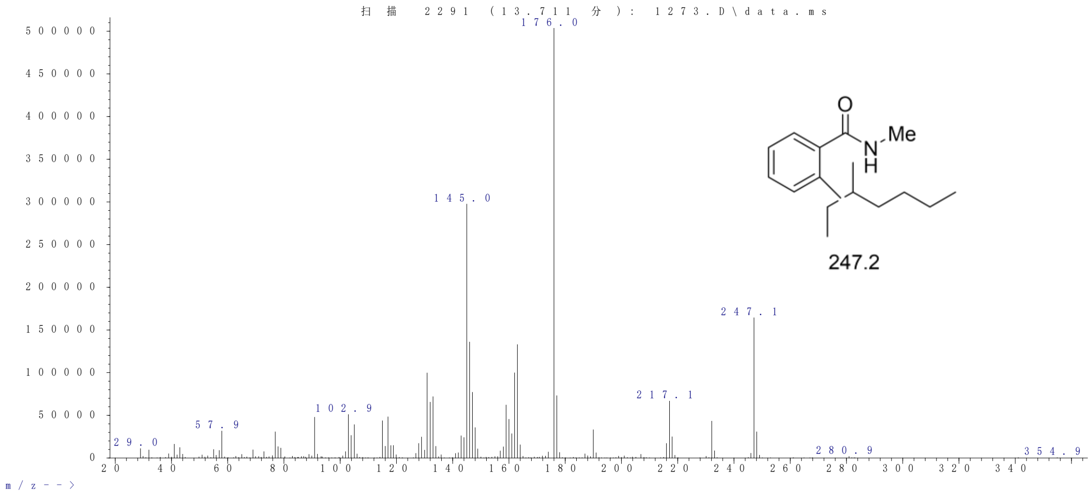
扫描 2291 (13.711 分): 1273.D\data.ms
176.0
145.0
247.1
217.1
102.9
57.9
29.0
280.9
354.9
m/z-->
O
N
Me
H
247.2

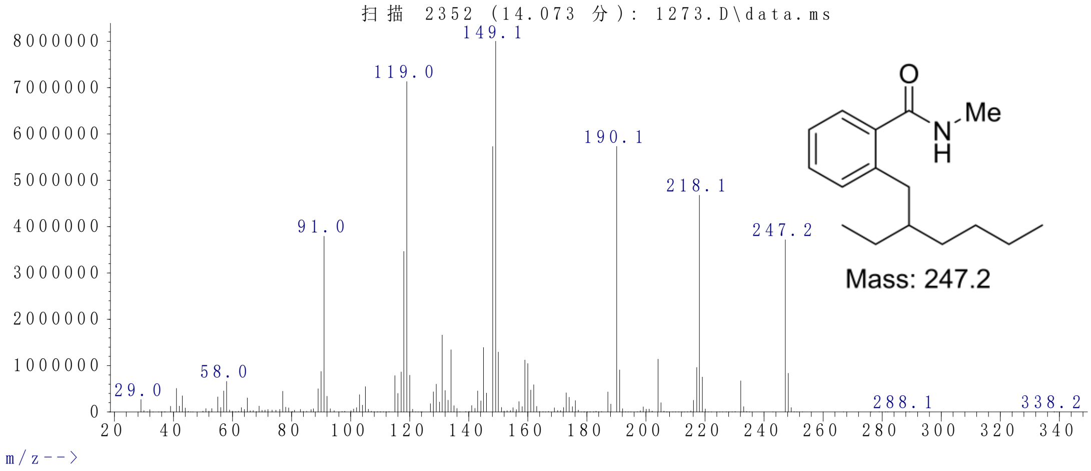
扫描 2352 (14.073 分): 1273.D\data.ms
149.1
119.0
190.1
218.1
91.0
247.2
58.0
29.0
288.1
338.2
m/z-->
O
N
Me
H
Mass: 247.2

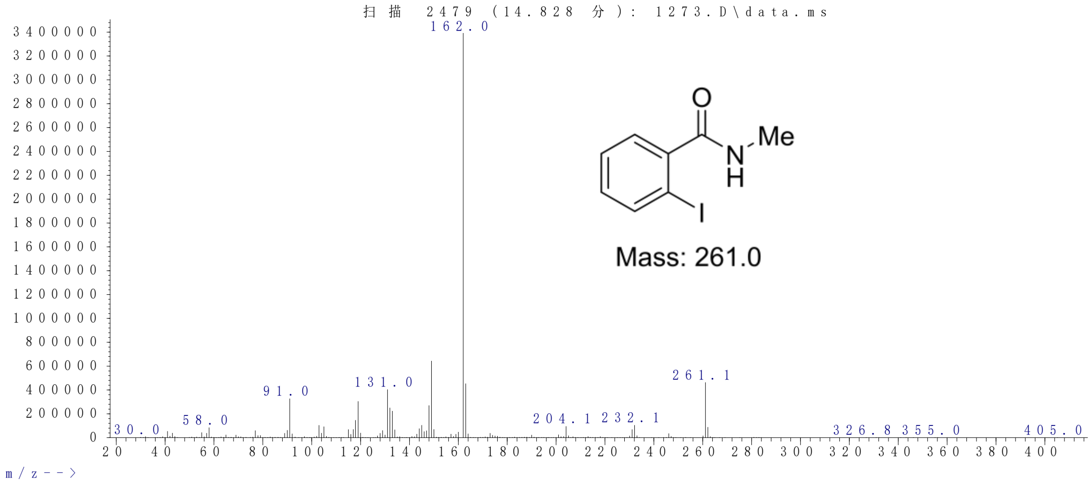
扫描 2479 (14.828 分): 1273.D\data.ms
162.0
261.1
131.0
91.0
58.0
30.0
204.1
232.1
326.8
355.0
405.0
m/z-->
O
N
Me
H
I
Mass: 261.0

7.GC-MS of the reaction mixture from 1a with 2i

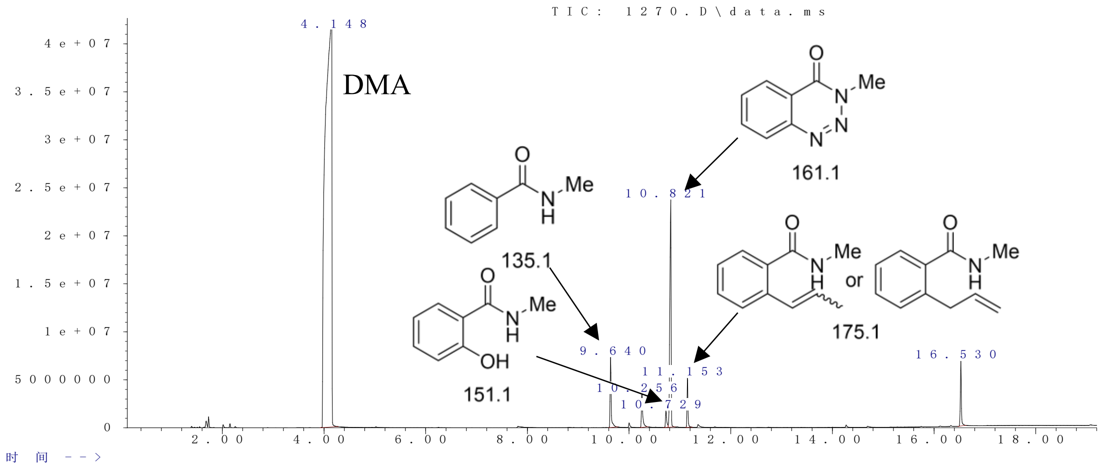

Data Path : D:\2025DATA\

Data File : 1273.D

Acq On : 22 May 2025 00:54

Operator :

Sample : HYY

Misc :

ALS Vial: 1 Sample Multiplier: 1

Integration Parameters : autoint1.e

Integrator : ChemStation

Method : D:\GCMS Methods- 1.M

Title :

Signal：TIC: 1273.D\data.ms

peak #	R.T. min	first scan	max scan	last scan	PK TY	peak height	corr. area	corr. % max	%of total
1	9.640	1600	1606	1629	BB	6853274	106312756	25.53%	13.651%
2	10.256	1703	1710	1729	BB	3426078	64043733	15.38%	8.223%
3	10.729	1782	1790	1797	BV	1632028	28470685	6.84%	3.656%
4	10.821	1797	1805	1818	VB	22878824	416402374	100.00%	53.466%
5	11.153	1854	1861	1875	BB	5105026	67768688	16.27%	8.701%
6	16.530	2756	2765	2781	BB	6411171	95820798	23.01%	12.303%

Sum of corrected areas： 778819033

1.M Tues May 10:12:37 2025

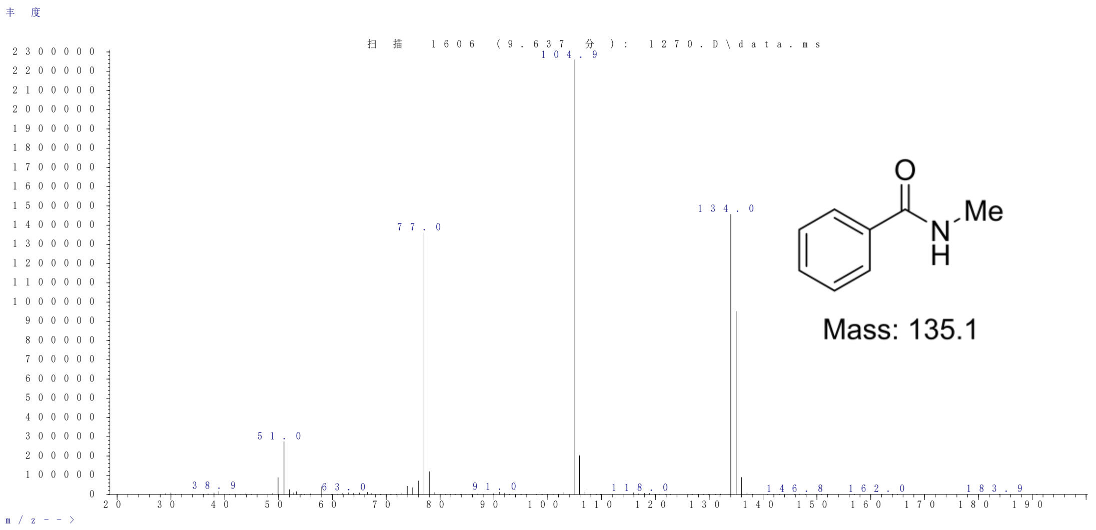
丰 度
扫 描 1606 (9.637 分): 1270.D\data.ms
104.9
134.0
77.0
51.0
38.9
63.0
91.0
118.0
146.8
162.0
183.9
O
N
H
Me
Mass: 135.1
m/z-->

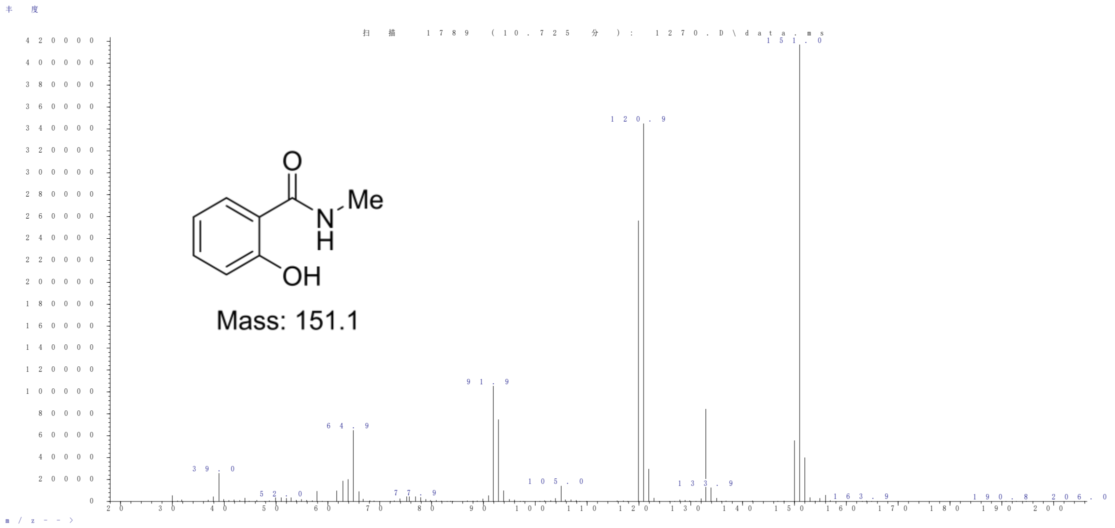
丰 度
扫 描 1789 (10.725 分): 1270.D\data.ms
151.0
120.9
91.9
64.9
39.0
52.0
77.9
105.0
133.9
163.9
190.8
206.0
O
N
H
Me
OH
Mass: 151.1
m/z-->

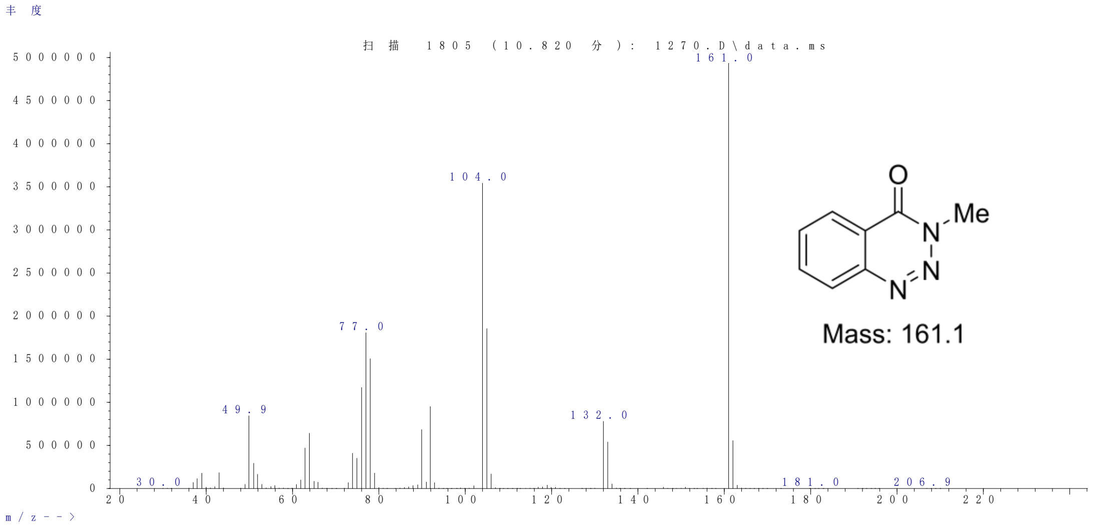
丰 度
扫 描 1805 (10.820 分): 1270.D\data.ms
161.0
104.0
77.0
49.9
132.0
30.0
181.0
206.9
O
N
Me
N
N
Mass: 161.1
m/z-->

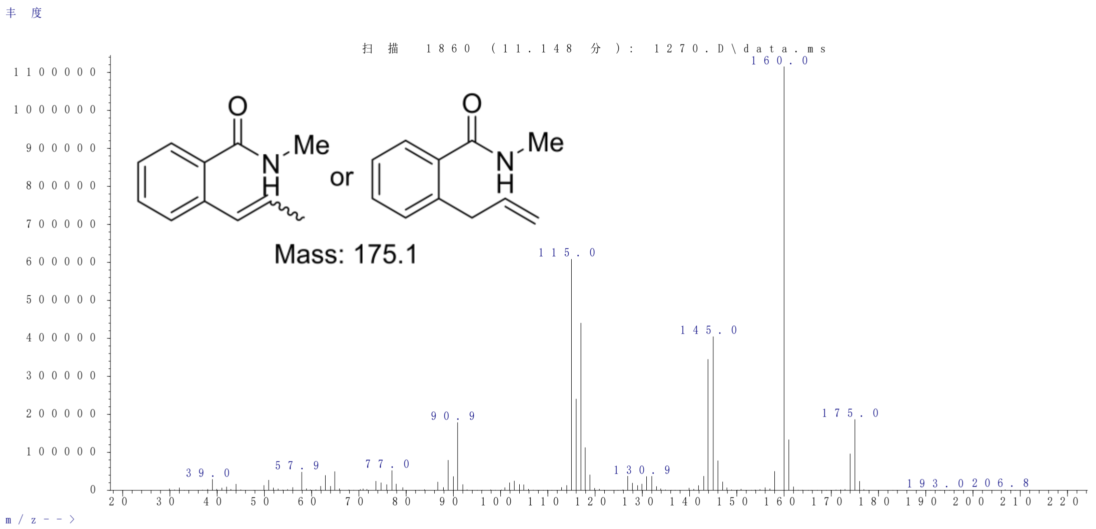

8. GC-MS of the reaction mixture from the competing reaction

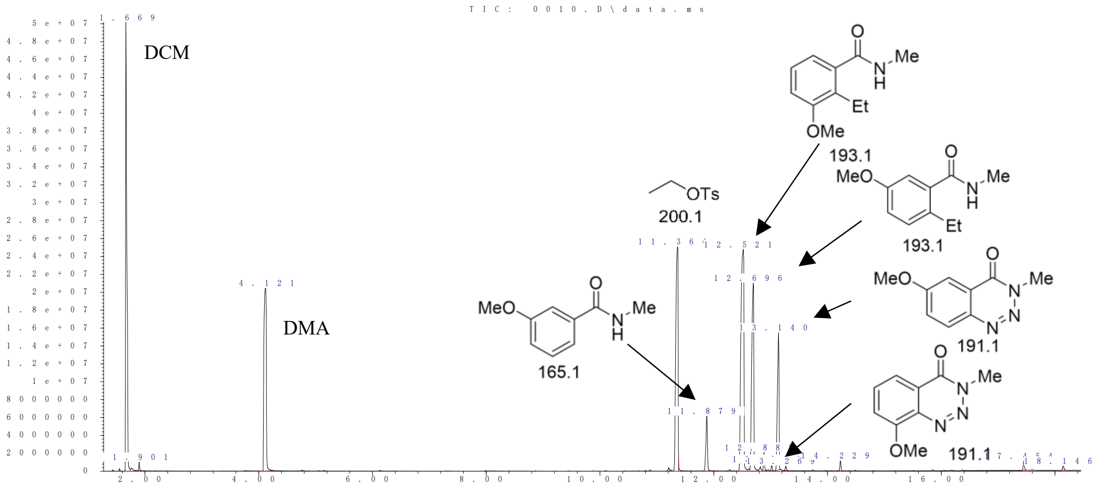

Data Path : D:\2025DATA\

Data File : 0010.D

Acq On : 09 Jan 2025 17:08

Operator :

Sample : HYY-1

Misc :

ALS Vial: 3 Sample Multiplier: 1

Integration Parameters : autoint1.e

Integrator : ChemStation

Method : D:\GCMS Methods- 1.M

Title :

Signal：TIC: 0010.D\data.ms

peak	R.T.	first	max	last	PK	peak	corr.	corr.	%of
#	min	scan	scan	scan	TY	height	area	% max	total

---	-----	-----	----	----	---	-------	-------	------	-------
1	1.901	301	305	315	VB	982440	10725441	1.19%	0.410%
2	11.364	1882	1896	1899	VB 2	25079521	685431365	76.13%	26.196%
3	11.879	1958	1983	2002	BB	6086055	116325119	12.92%	4.446%
4	12.521	2076	2091	2104	BV 2	24842808	900311220	100.00%	34.409%
5	12.696	2109	2120	2135	VV	20934048	516474066	57.37%	19.739%
6	12.885	2147	2152	2161	VV	2005649	27038091	3.00%	1.033%
7	13.020	2170	2175	2185	VV	743687	9578291	1.06%	0.366%
8	13.140	2185	2195	2201	PV	15203372	305321096	33.91%	11.669%
9	13.269	2212	2217	2230	BB 2	493247	7089019	0.79%	0.271%
10	14.229	2373	2378	2391	VB	1099309	14772106	1.64%	0.565%
11	17.454	2906	2921	2933	BB	1019534	15607599	1.73%	0.597%
12	18.146	3025	3037	3048	BB 2	462679	7848028	0.87%	0.300%

Sum of corrected areas： 2616521441， 1.M Tues Mar 14:29:32 2025

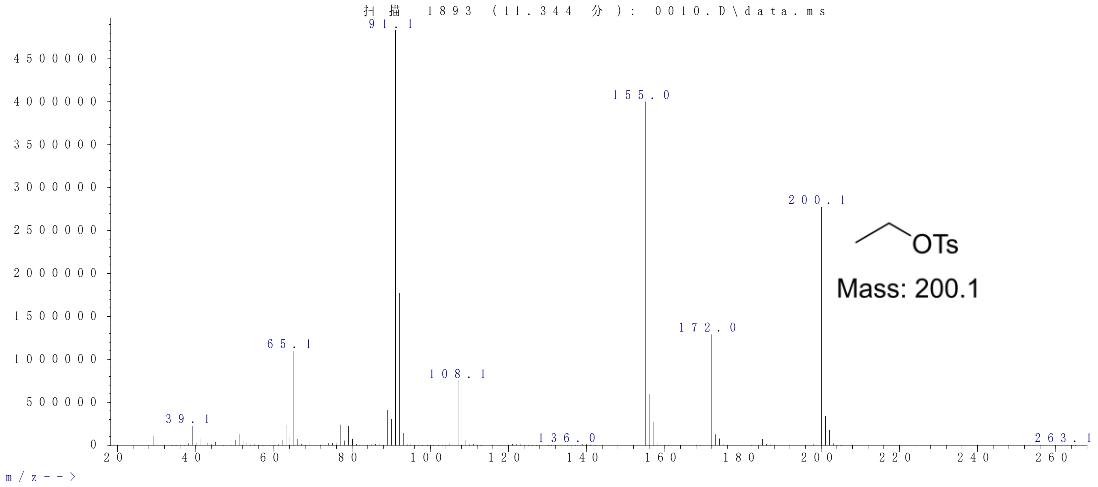

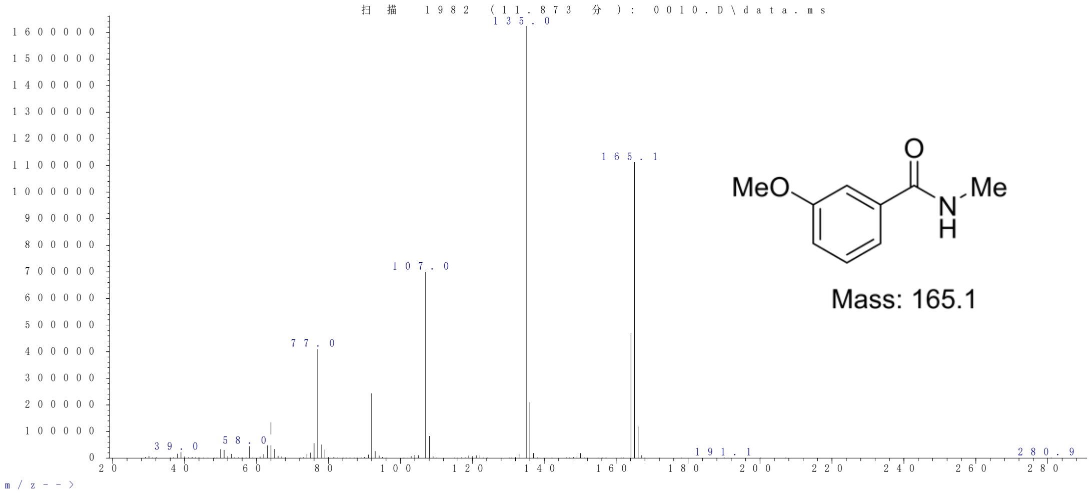

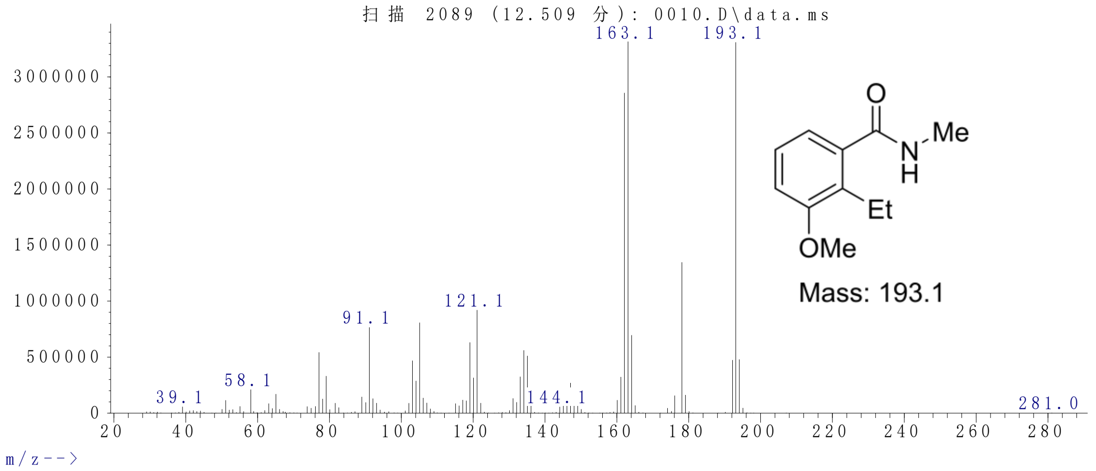
扫描 2089 (12.509 分): 0010.D\data.ms
163.1
193.1
121.1
91.1
58.1
39.1
144.1
281.0
m/z-->
Mass: 193.1

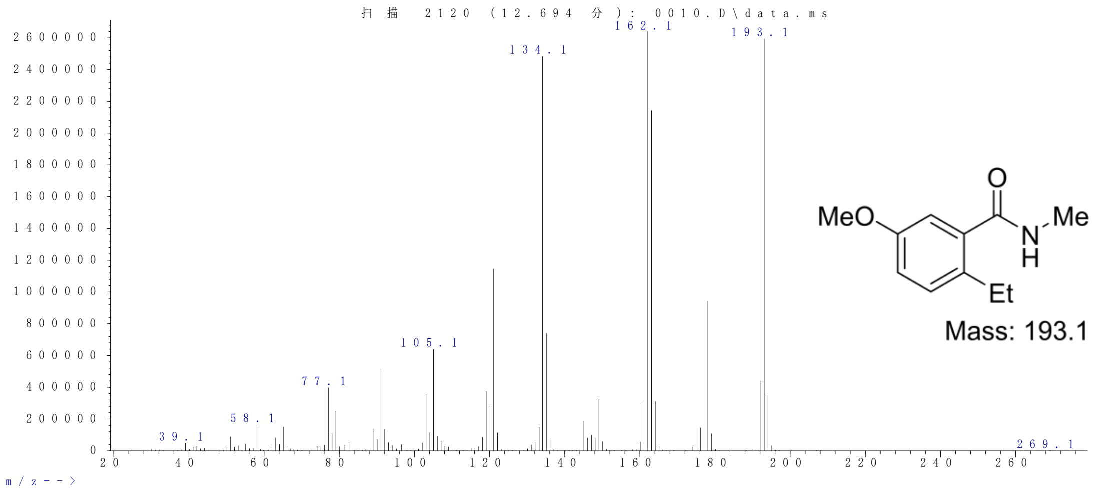
扫描 2120 (12.694 分): 0010.D\data.ms
162.1
193.1
134.1
105.1
77.1
58.1
39.1
269.1
m/z-->
Mass: 193.1

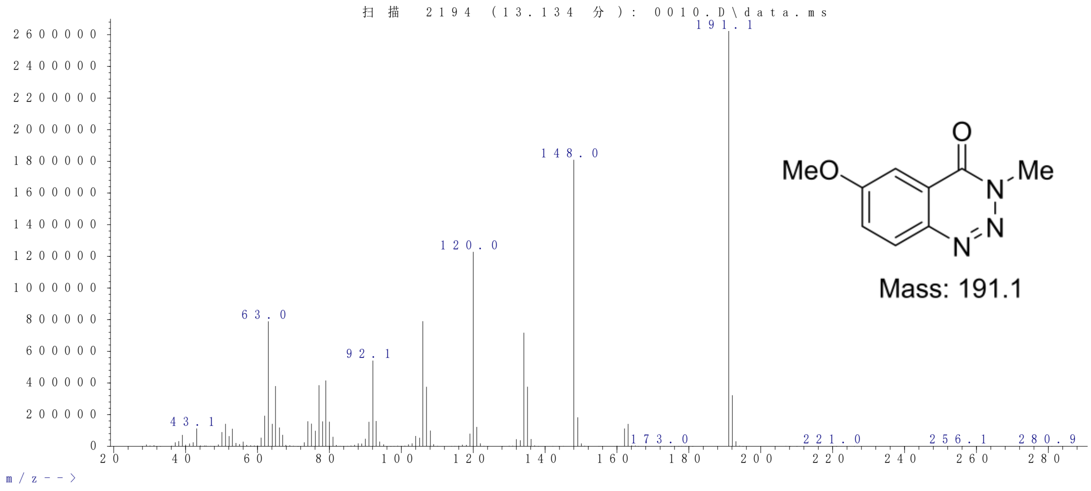
扫描 2194 (13.134 分): 0010.D\data.ms
191.1
148.0
120.0
63.0
92.1
43.1
173.0
221.0
256.1
280.9
m/z-->
Mass: 191.1

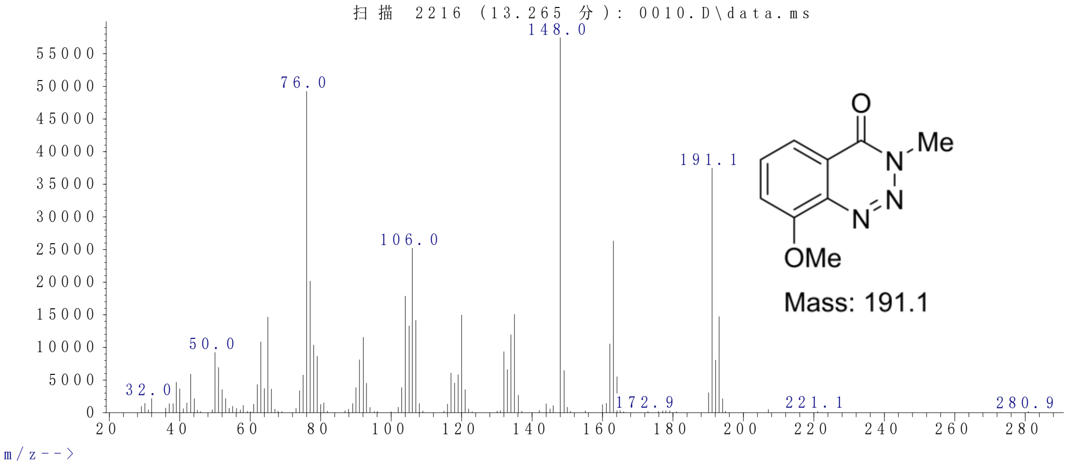
扫描 2216 (13.265 分): 0010.D\data.ms
55000
50000
45000
40000
35000
30000
25000
20000
15000
10000
5000
0
148.0
76.0
106.0
50.0
32.0
172.9
191.1
221.1
280.9
20 40 60 80 100 120 140 160 180 200 220 240 260 280
m/z-->
O
N
Me
N
N
OMe
Mass: 191.1

9. ^{1}H NMR and GC-MS of radical clock experiment

1) ^{1}H NMR of the crude product from the radical clock experiment

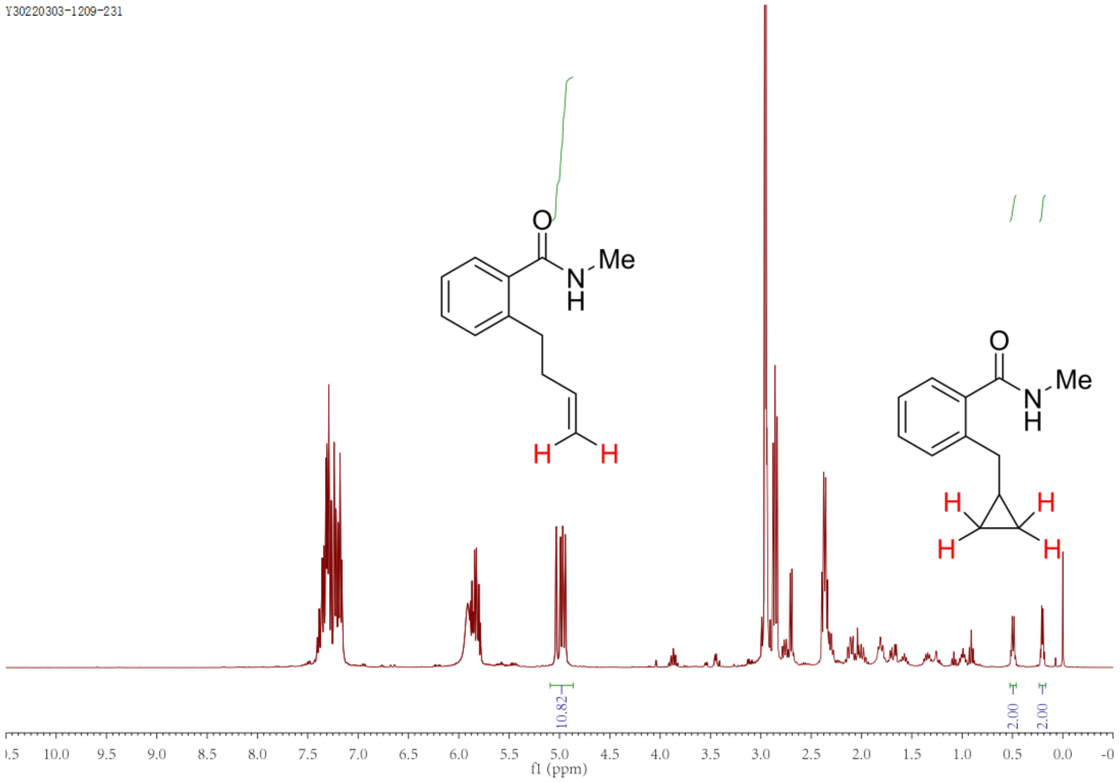

2) GC-MS of the reaction mixture from the radical clock experiment

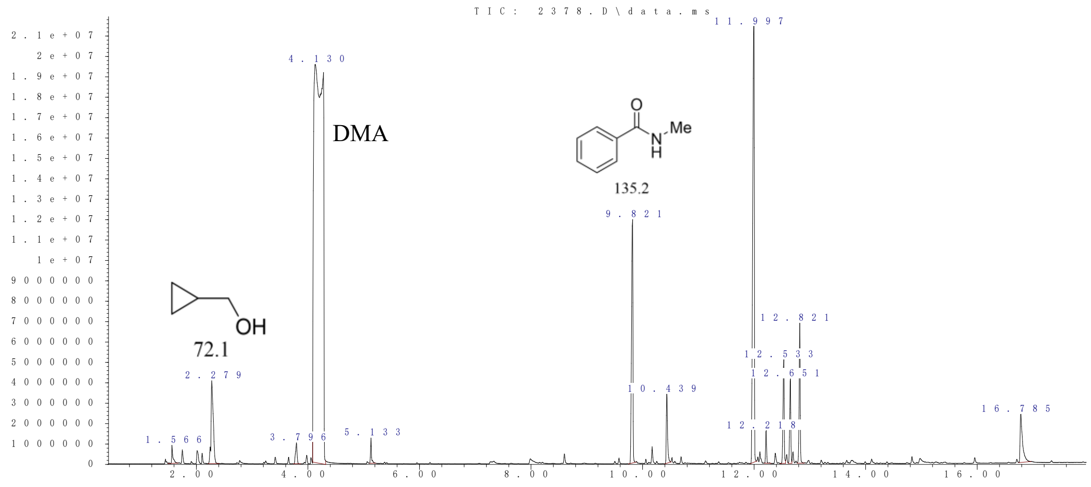

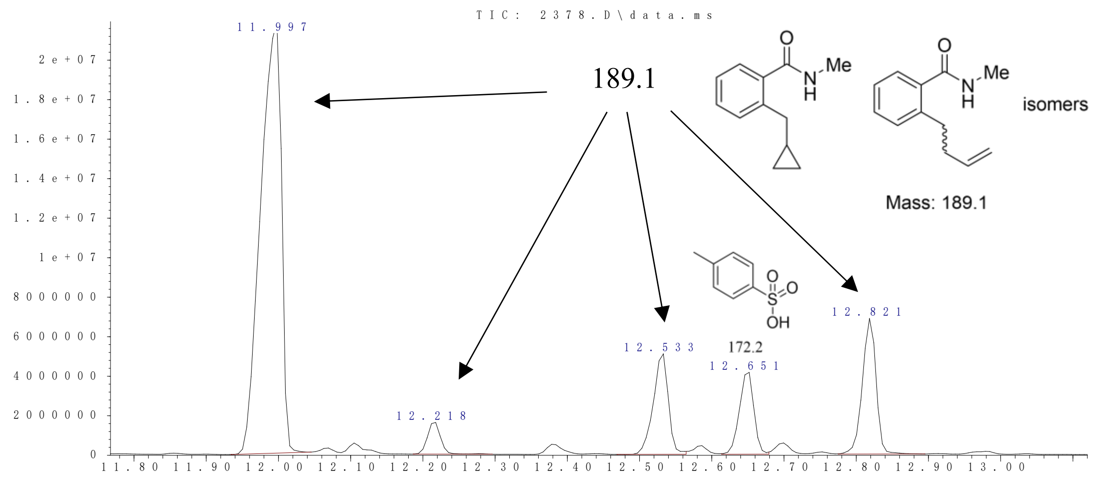

Data Path : D:\2024DATA\

Data File : 2378.D

Acq On : 21 Dec 2024 15:37

Operator :

Sample : HYY

Misc :

ALS Vial: 1 Sample Multiplier: 1

Integration Parameters : autoint1.e

Integrator : ChemStation

Method : D:\GCMS Methods- 1.M

Title :

Signal：TIC: 2378.D\data.ms

peak #	R.T. min	first scan	max scan	last scan	PK TY	peak height	corr. area	corr. % max	%of total
---	-----	-----	----	----	---	-------	-------	------	-------
1	1.566	236	249	265	VB	825511	13746378	3.17%	1.170%
2	2.279	357	368	389	BB 2	4016303	112765185	26.00%	9.599%
3	3.796	615	624	635	BB 3	1017081	19598411	4.52%	1.668%
4	5.133	844	848	861	BB	1174878	14960849	3.45%	1.274%
5	9.821	1625	1637	1647	BV	11820925	213652817	49.27%	18.188%
6	10.439	1734	1741	1752	BV	3321473	52694427	12.15%	4.486%
7	11.997	1992	2003	2011	BV	21857877	433674462	100.00%	36.917%
8	12.218	2035	2040	2053	BB	1597353	20248418	4.67%	1.724%
9	12.533	2082	2093	2098	BV	5006440	73380352	16.92%	6.247%
10	12.651	2107	2113	2118	VV 2	4140923	57897414	13.35%	4.929%
11	12.821	2134	2141	2154	VB	6644247	90427693	20.85%	7.698%
12	16.785	2802	2808	2837	VB	2361586	71668846	16.53%	6.101%

Sum of corrected areas： 1174715251

1.M Tues Mar 14:40:11 2025

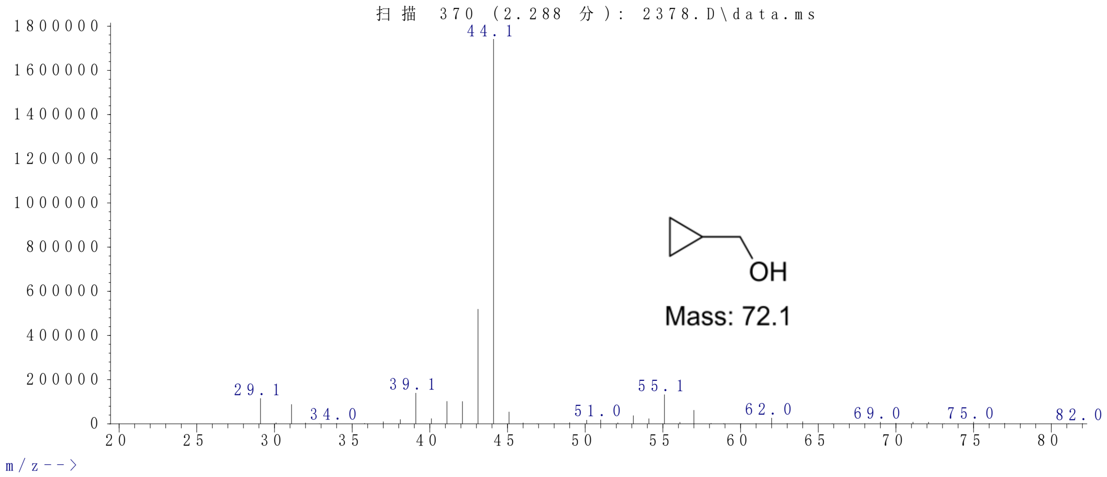
扫描 370 (2.288 分): 2378.D\data.ms
Mass: 72.1
m/z-->

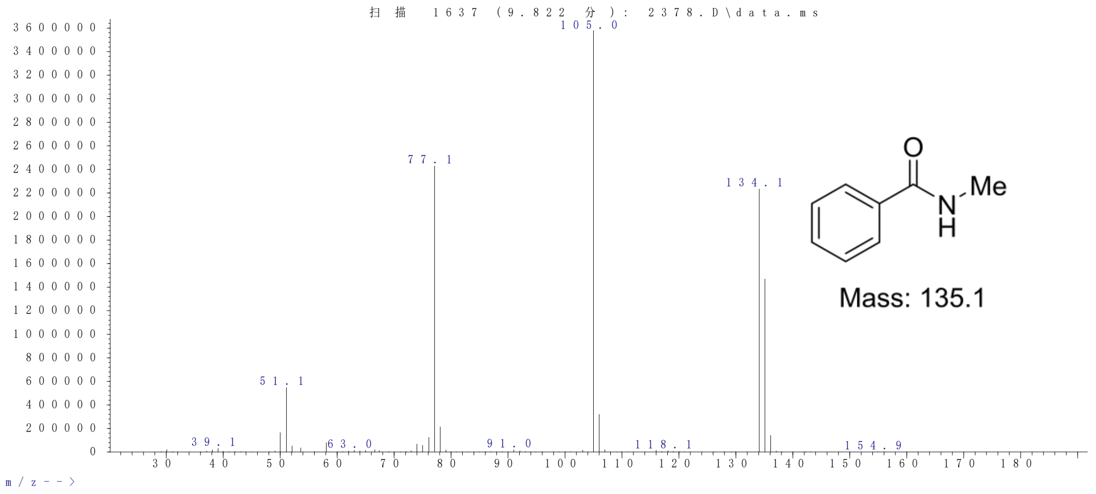
扫描 1637 (9.822 分): 2378.D\data.ms
Mass: 135.1
m/z-->

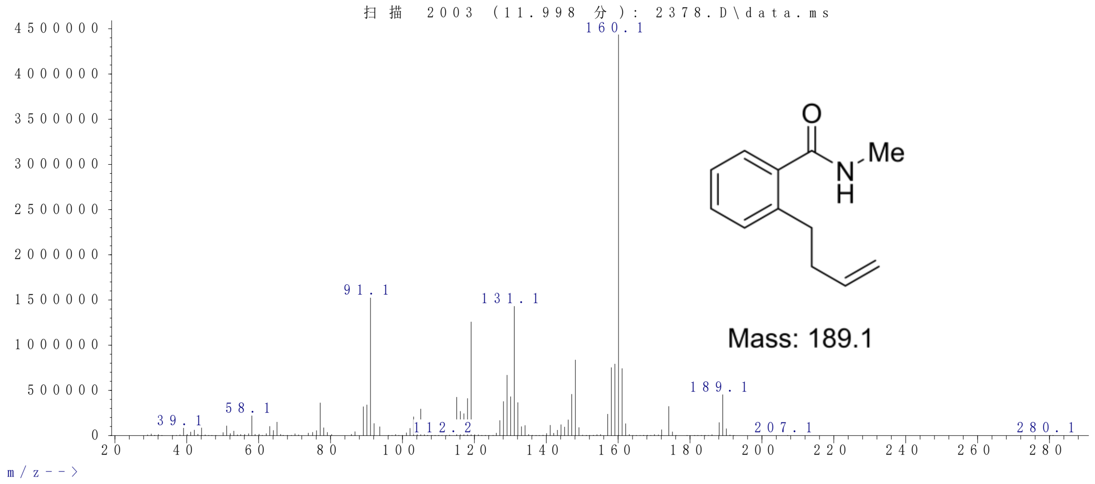
扫描 2003 (11.998 分): 2378.D\data.ms
Mass: 189.1
m/z-->

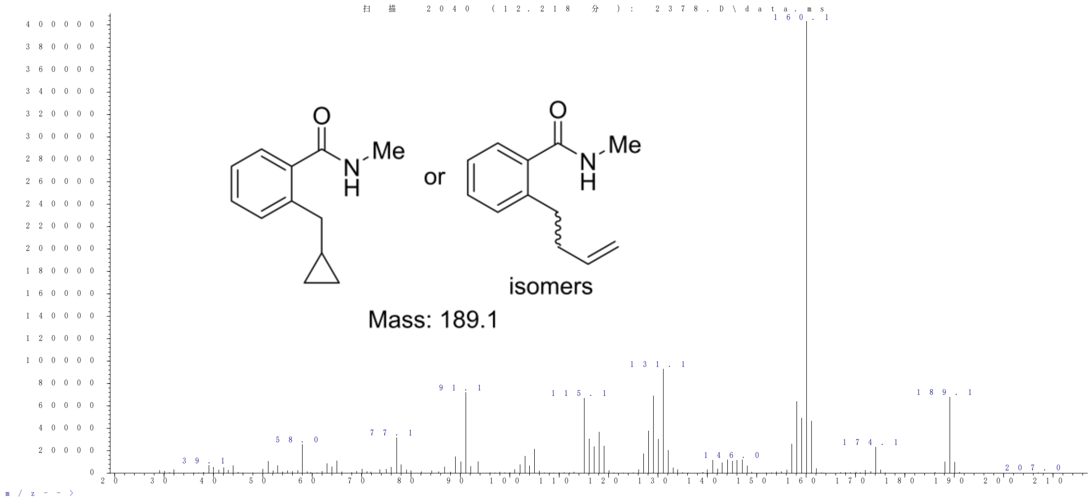
扫描 2040 (12.218 分): 2378.D\data.ms
160.1
131.1
91.1
115.1
189.1
77.1
58.0
39.1
146.0
174.1
207.0
Mass: 189.1
or
isomers
m/z-->

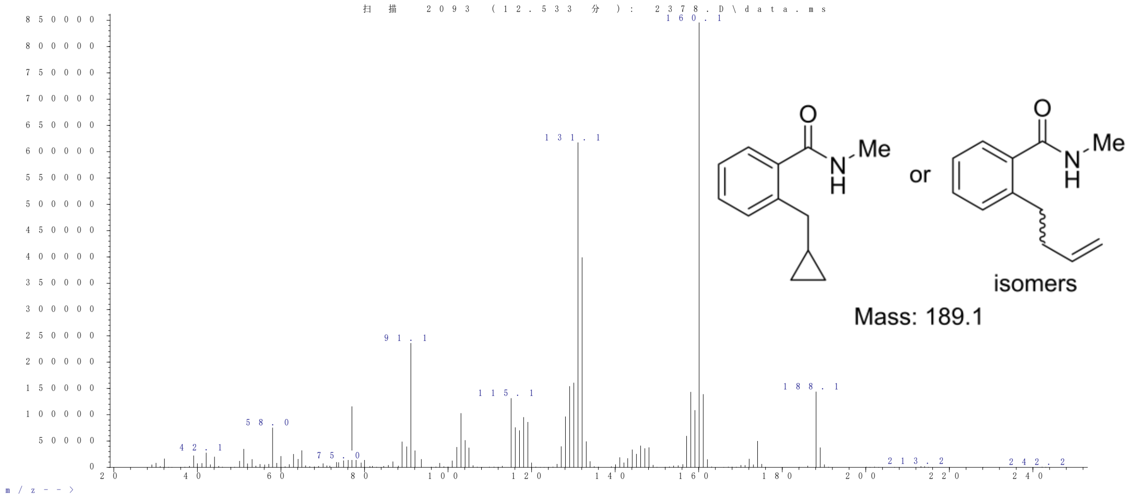
扫描 2093 (12.533 分): 2378.D\data.ms
160.1
131.1
91.1
115.1
188.1
58.0
42.1
75.0
213.2
242.2
Mass: 189.1
or
isomers
m/z-->

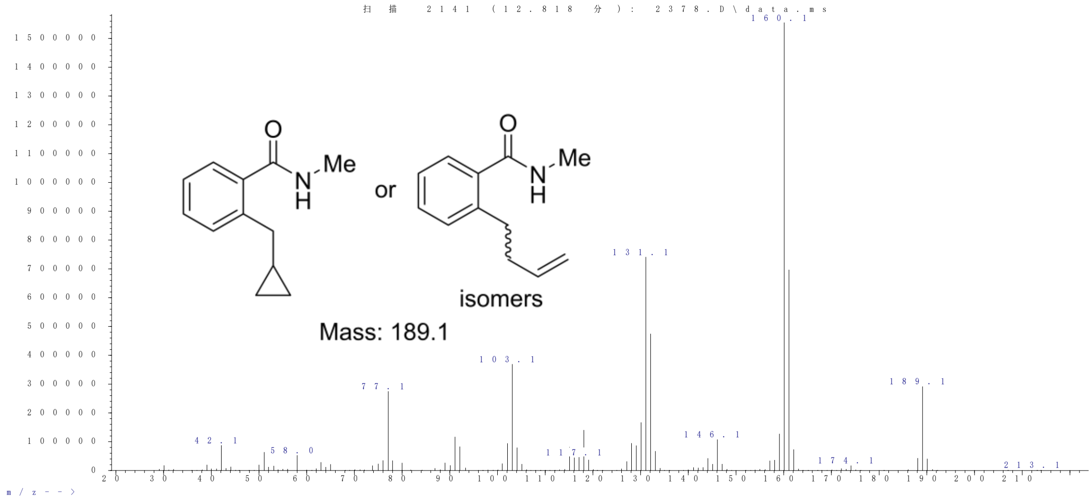
扫描 2141 (12.818 分): 2378.D\data.ms
160.1
131.1
103.1
77.1
189.1
146.1
42.1
58.0
117.1
174.1
213.1
Mass: 189.1
or
isomers
m/z-->

10. HRMS of new compounds

HRMS of 1g

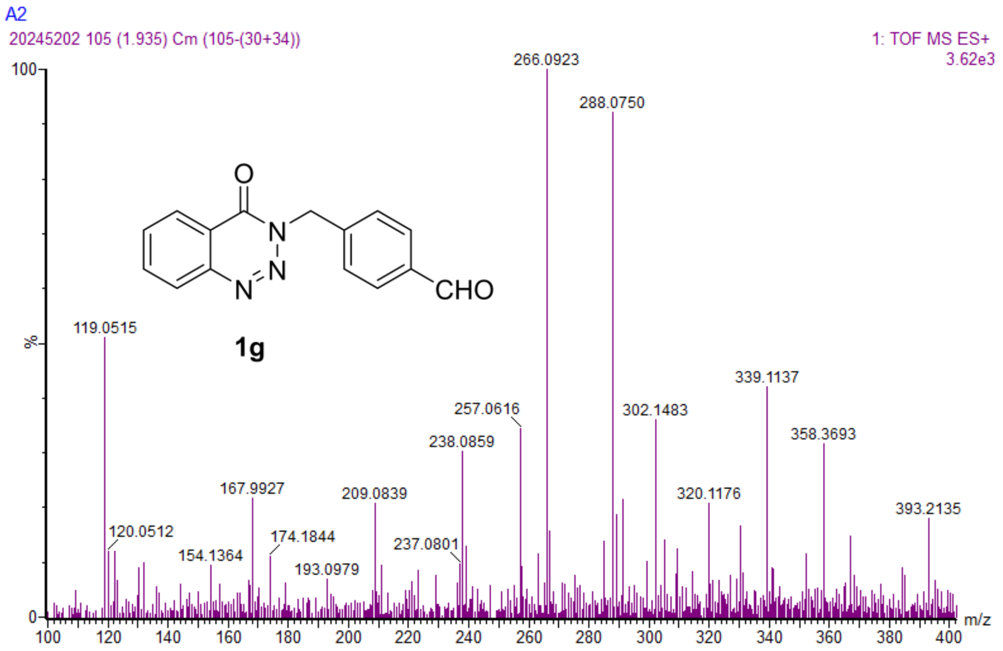

HRMS of 1h

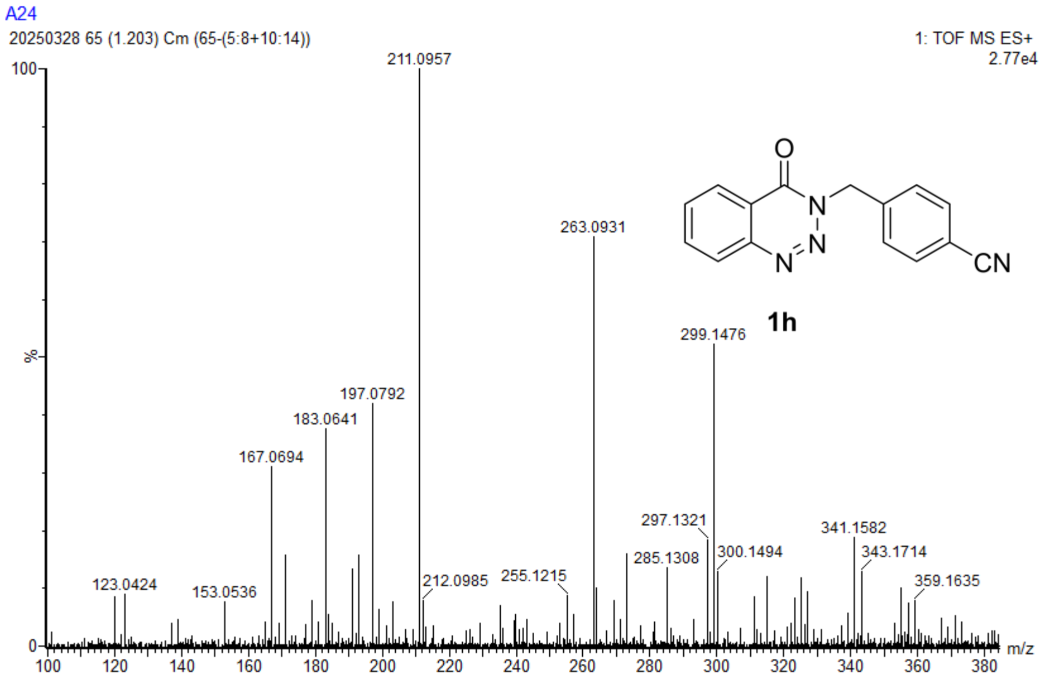

HRMS of 1o

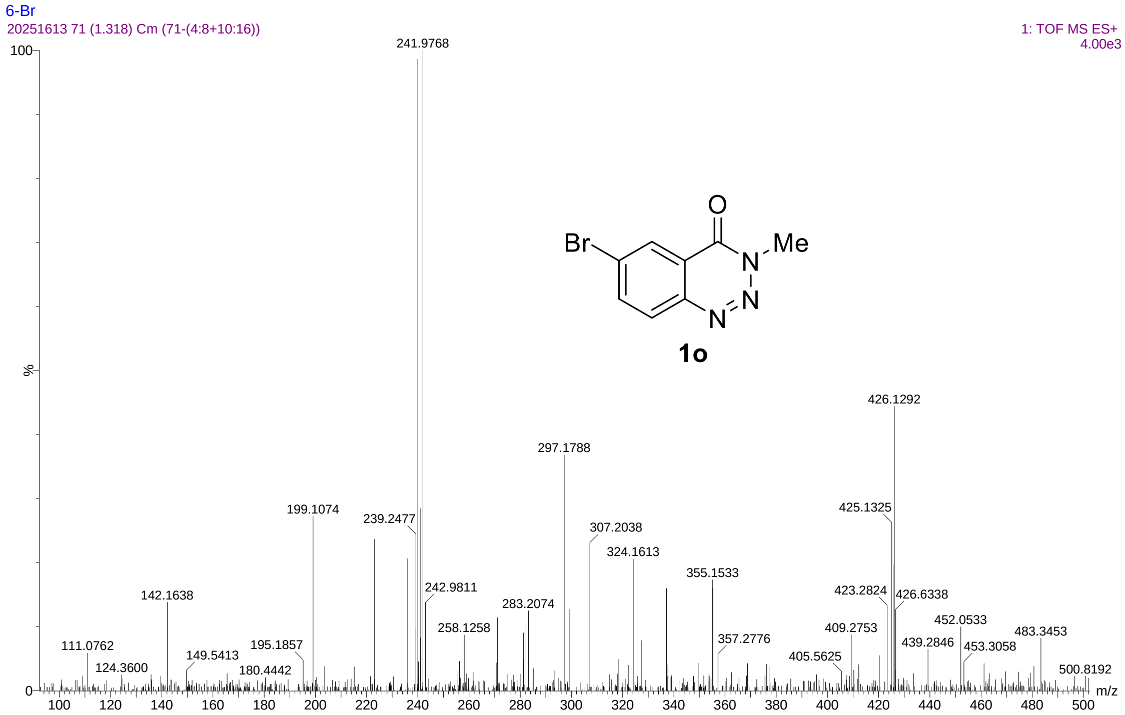

HRMS of 1p

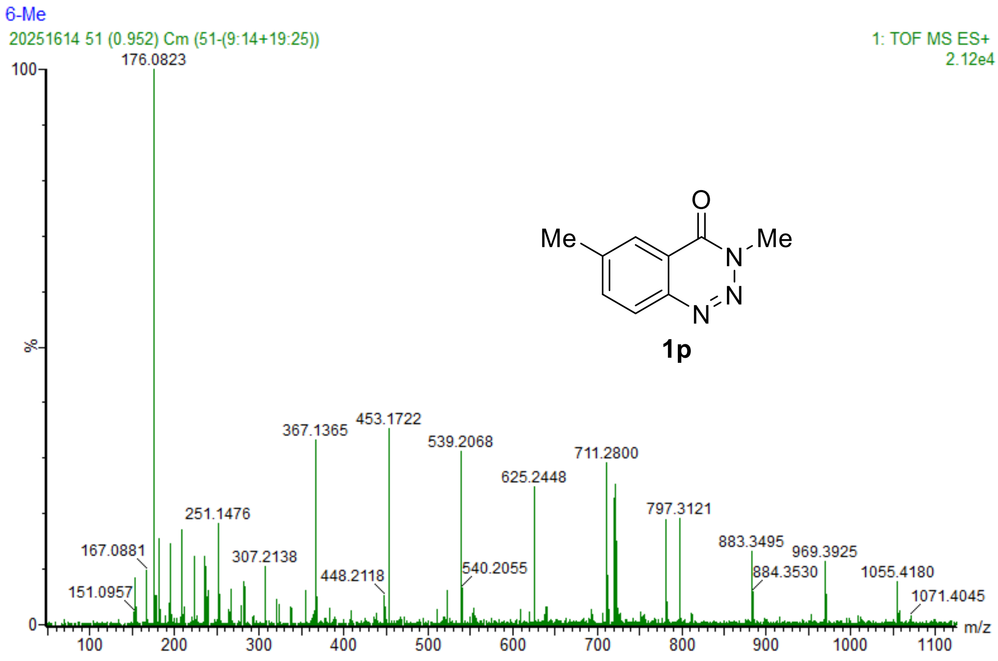

HRMS of 1s

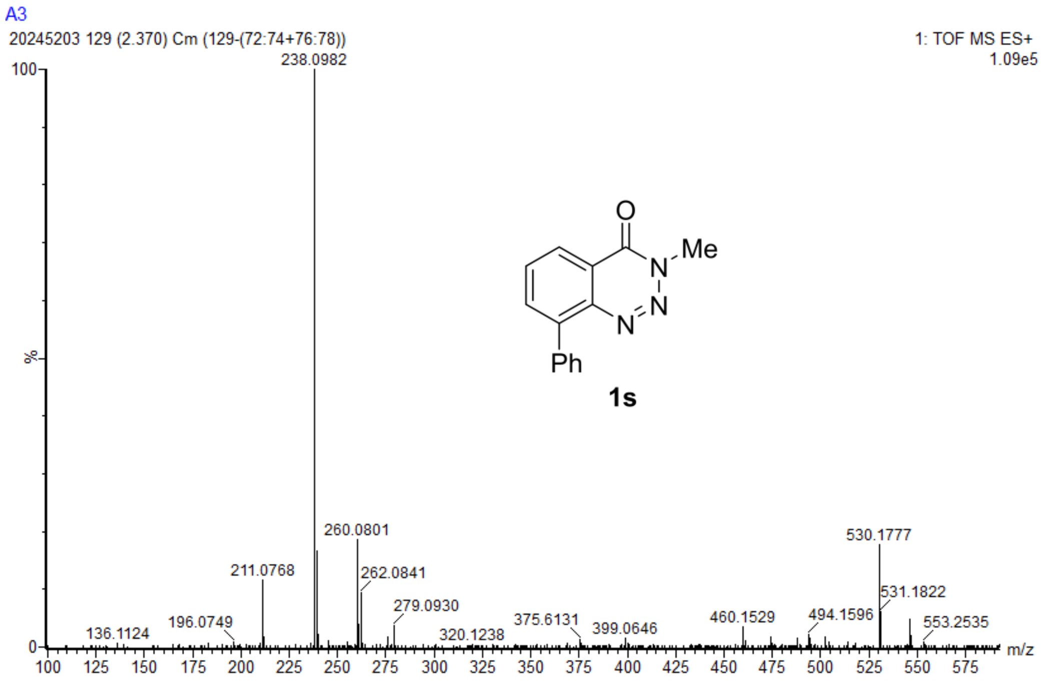

HRMS of 3ad

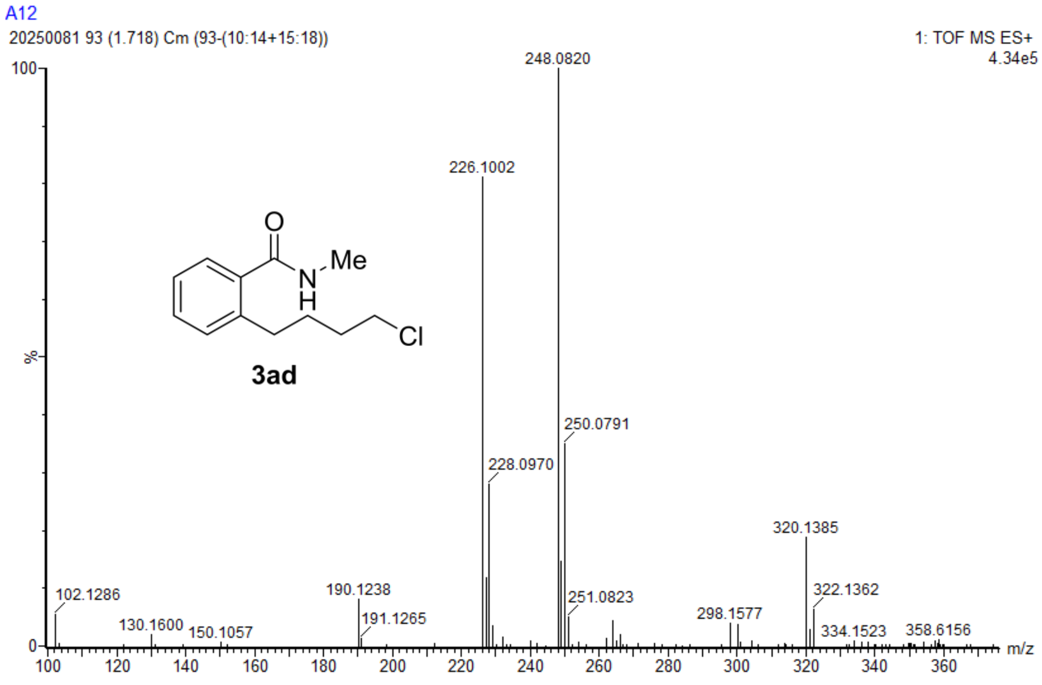

HRMS of 3af

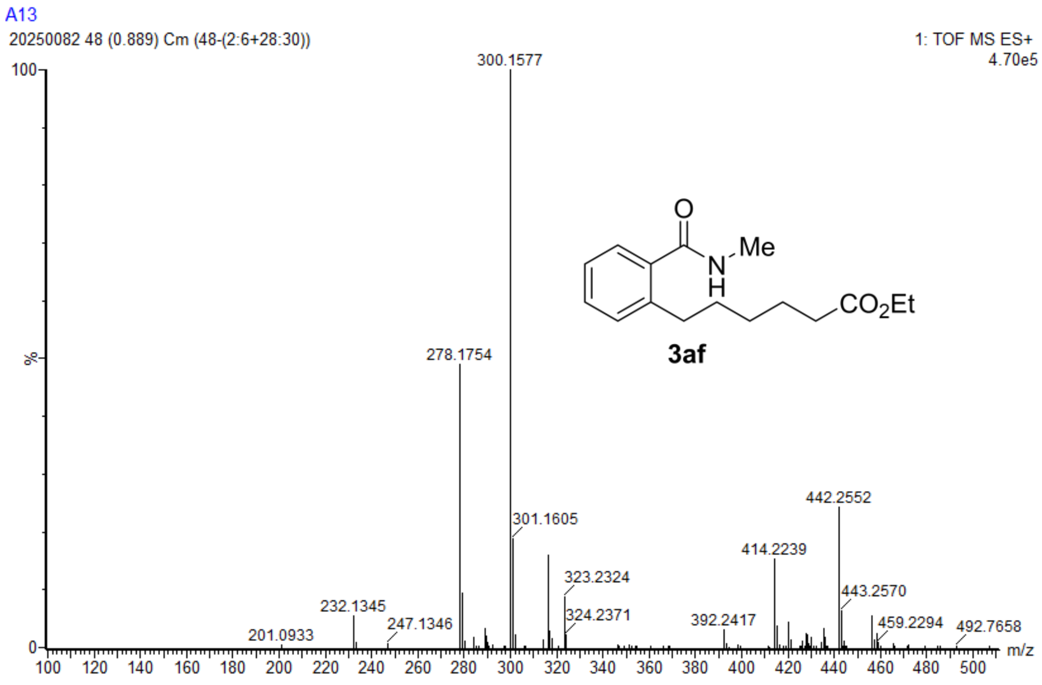

HRMS of 3eb

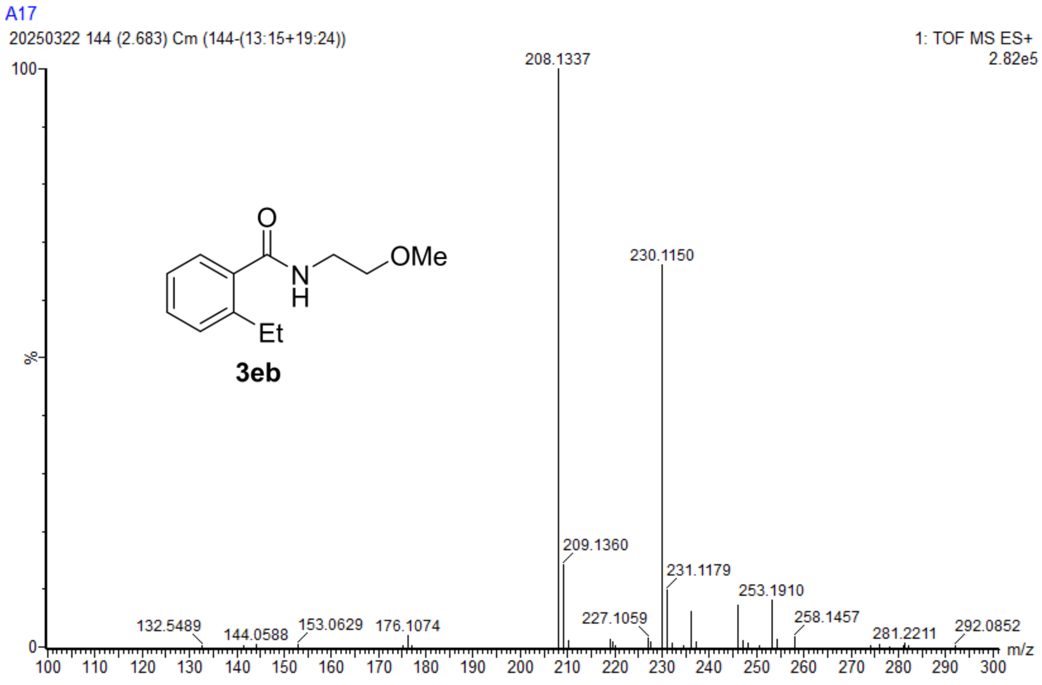

HRMS of 3gb

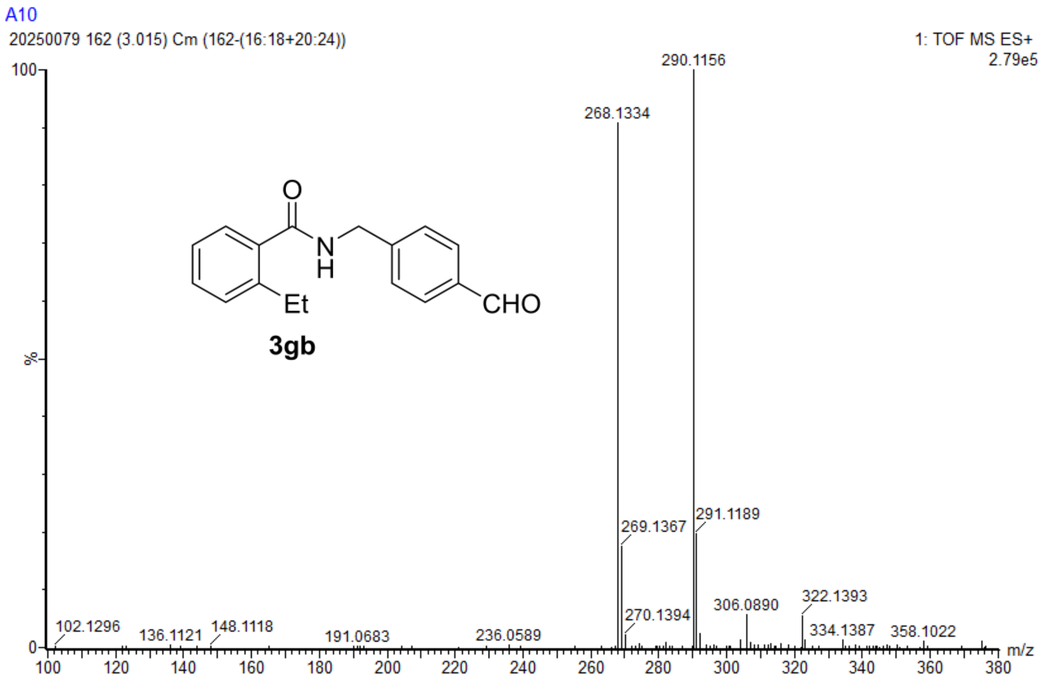

HRMS of 3hb

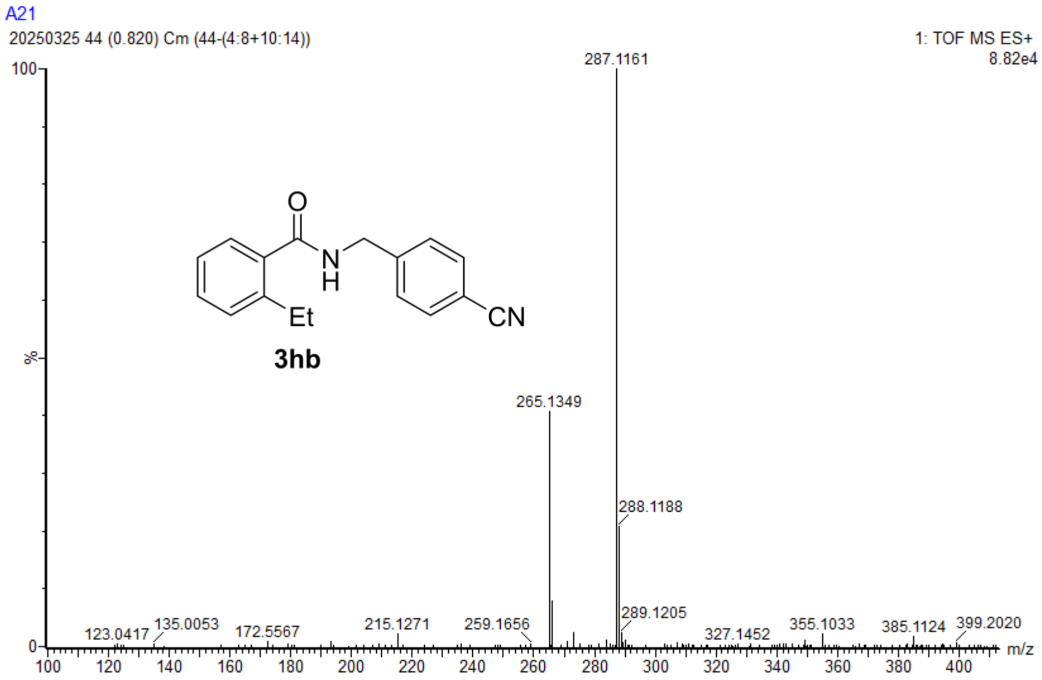

HRMS of 3ib

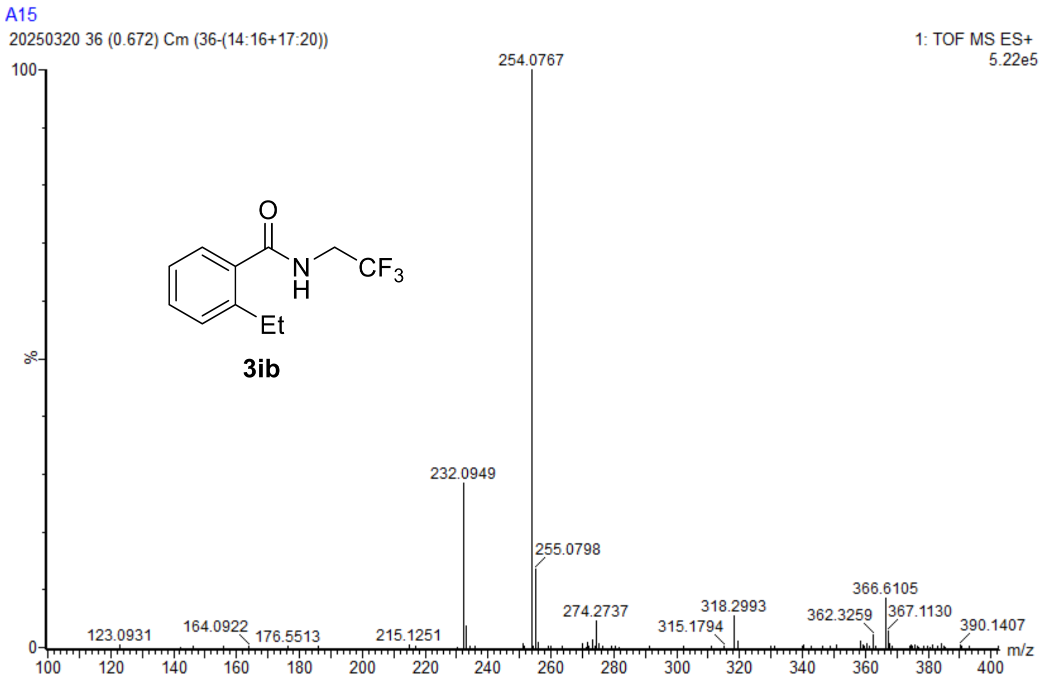

HRMS of 3jb

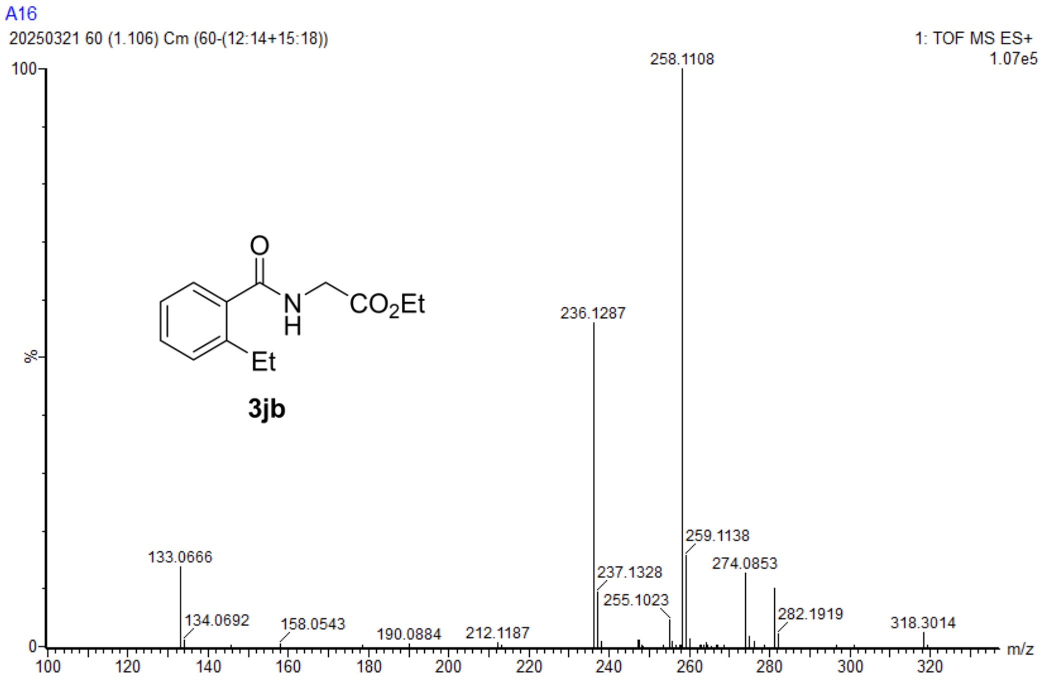

HRMS of 3lb

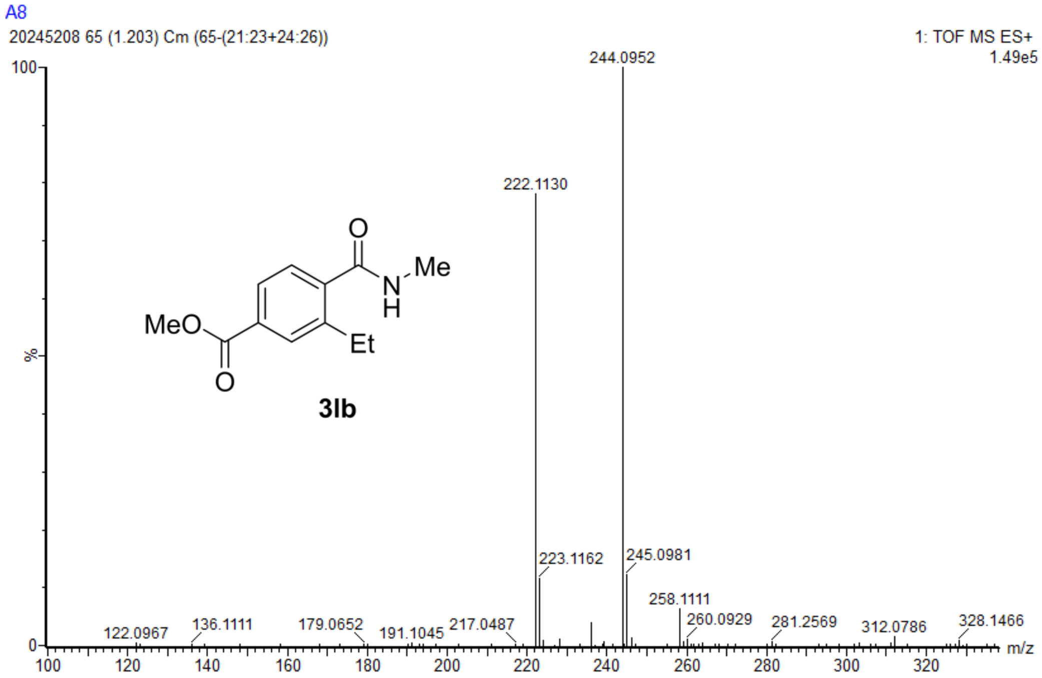

HRMS of 3mb

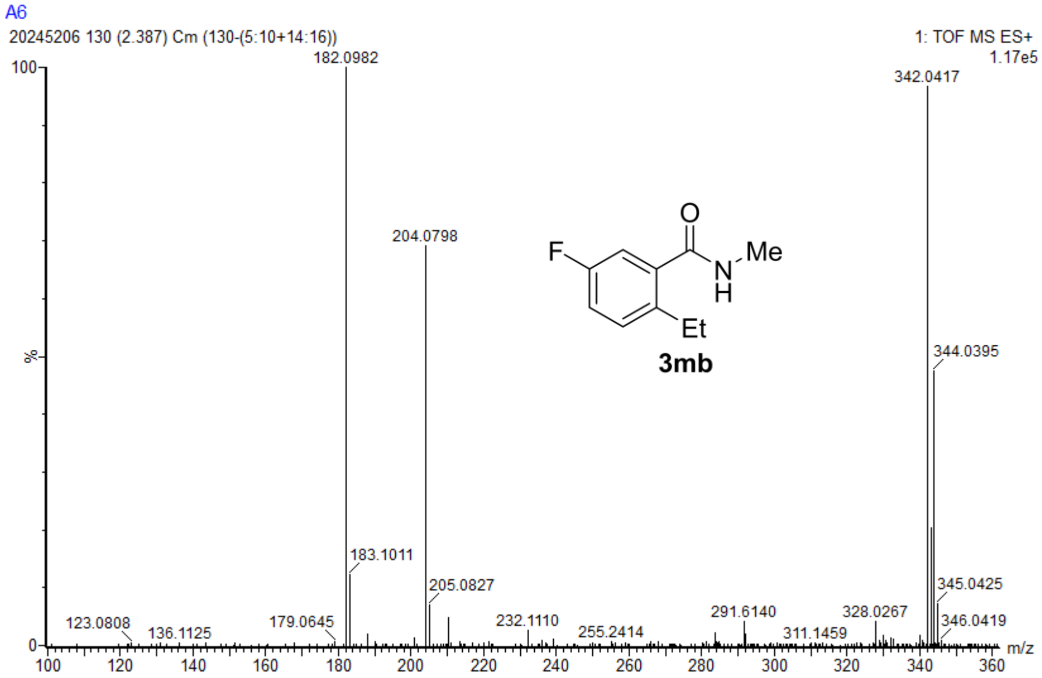

HRMS of 3nb

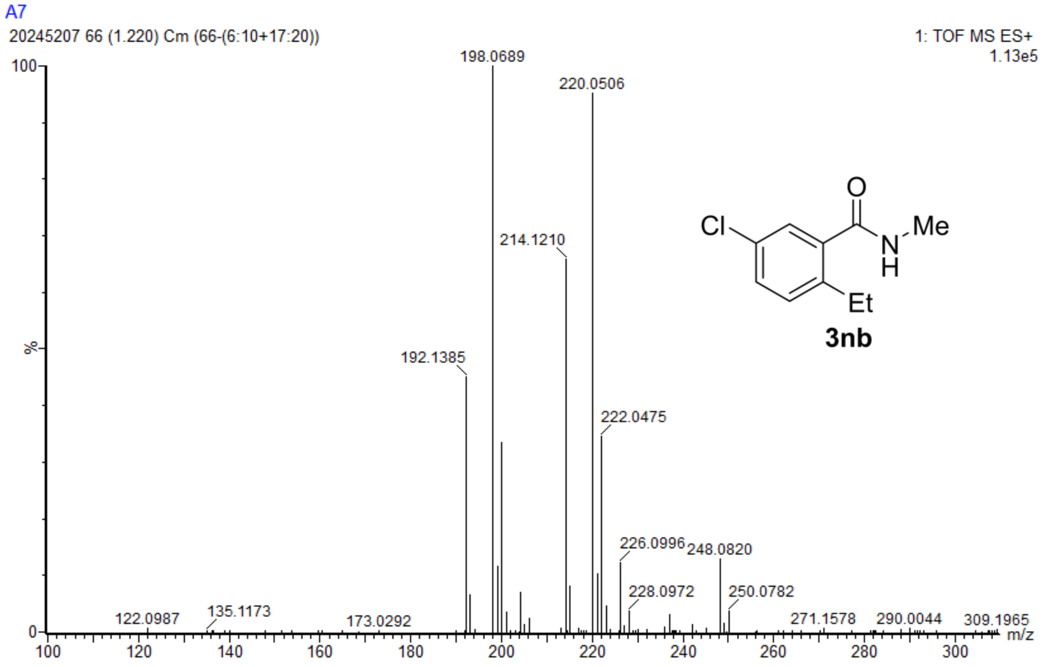

HRMS of 3pb

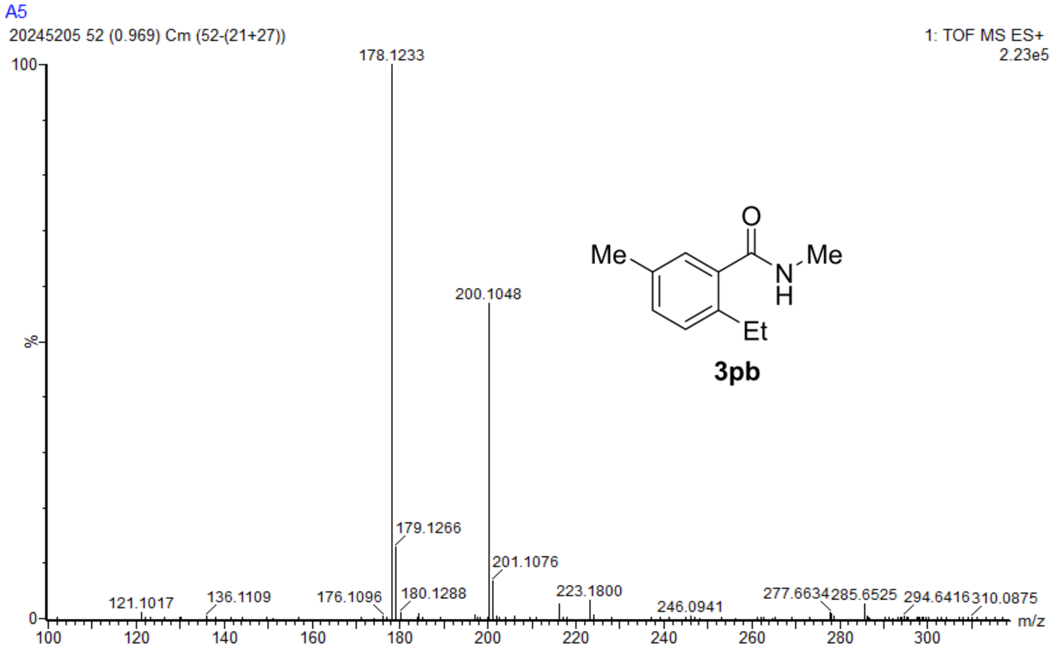

HRMS of 3qb

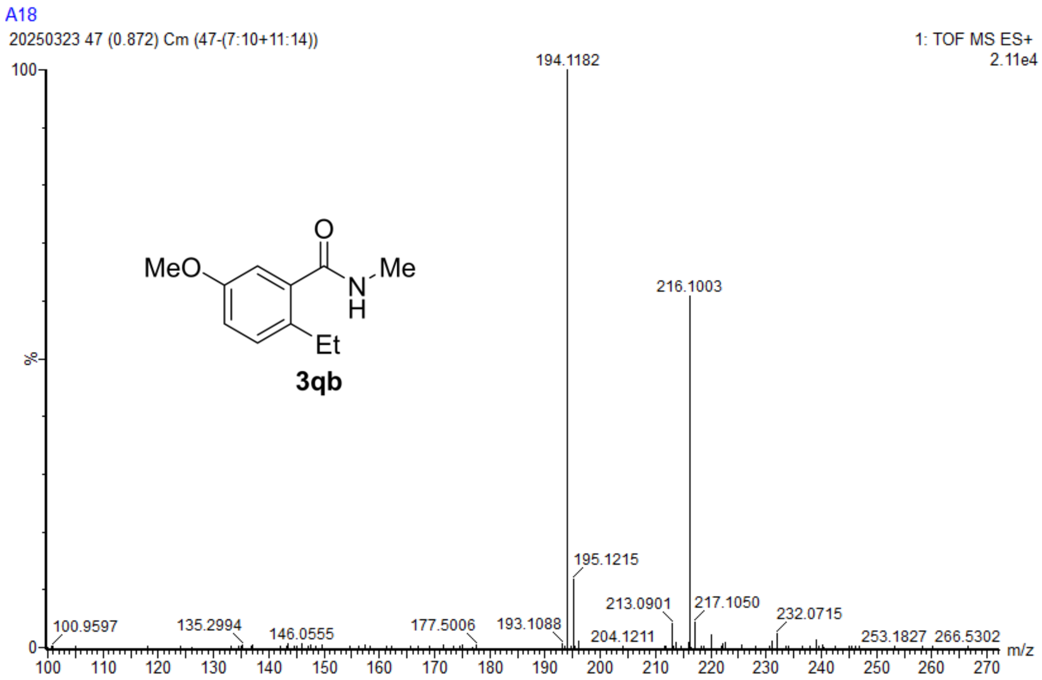

HRMS of 3rb

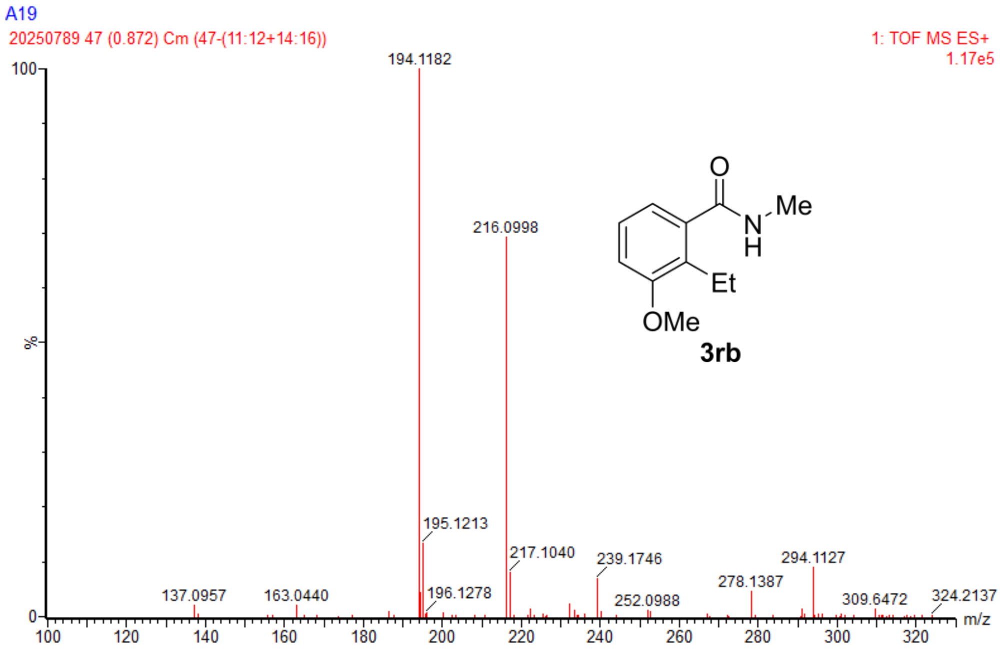

HRMS of 3sb

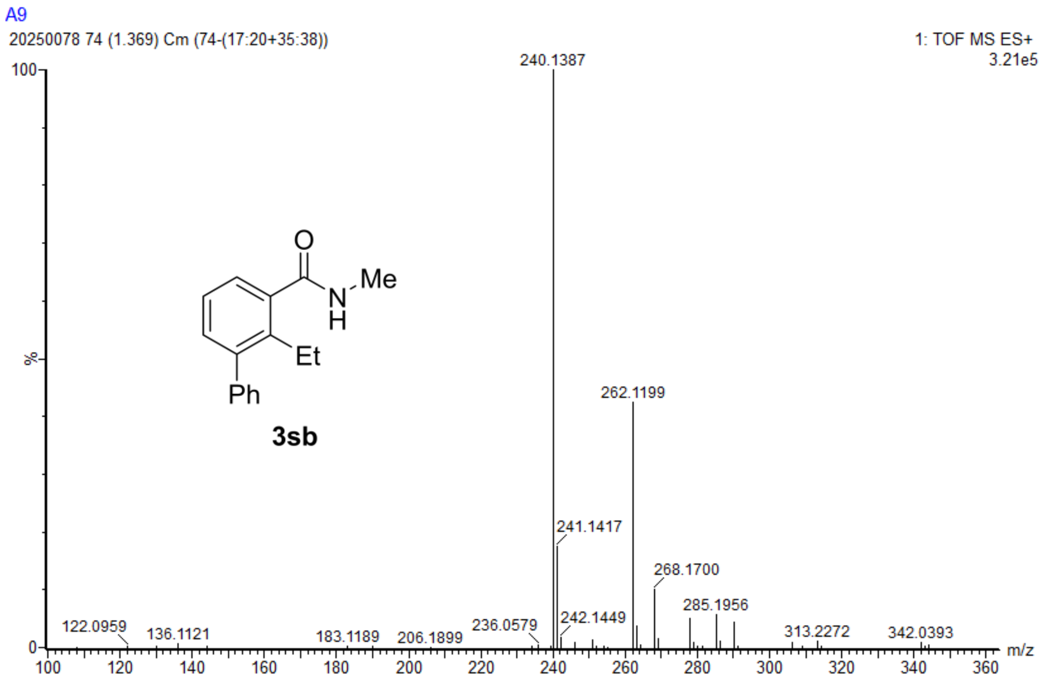

HRMS of 3tb

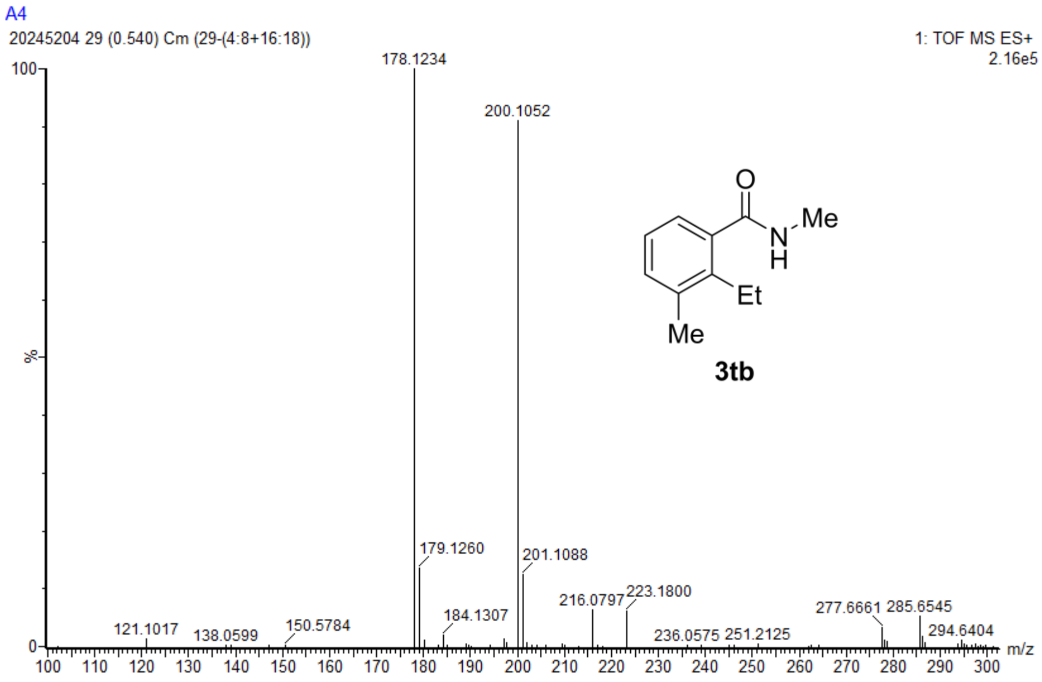

Supplement: Supplementary file 1 [file molecules-30-02397-s001.zip › molecules-3652567-supplementary.pdf]
